# Liquid–liquid phase separation underpins the formation of replication factories in rotaviruses

**DOI:** 10.15252/embj.2021107711

**Published:** 2021-09-15

**Authors:** Florian Geiger, Julia Acker, Guido Papa, Xinyu Wang, William E Arter, Kadi L Saar, Nadia A Erkamp, Runzhang Qi, Jack PK Bravo, Sebastian Strauss, Georg Krainer, Oscar R Burrone, Ralf Jungmann, Tuomas PJ Knowles, Hanna Engelke, Alexander Borodavka

**Affiliations:** ^1^ Department of Chemistry Ludwig‐Maximilians‐Universität München Munich Germany; ^2^ Department of Biochemistry University of Cambridge Cambridge UK; ^3^ International Center for Genetic Engineering and Biotechnology Trieste Italy; ^4^ Department of Chemistry University of Cambridge Cambridge UK; ^5^ Department of Physics and Center for Nanoscience Max Planck Institute of Biochemistry Munich Germany; ^6^ Institute of Pharmaceutical Sciences Karl‐Franzens‐Universität Graz Graz Austria; ^7^ Present address: Medical Research Council Laboratory of Molecular Biology (MRC LMB) Cambridge UK; ^8^ Present address: Department of Molecular Biosciences University of Texas at Austin Austin TX USA

**Keywords:** biomolecular condensates, microfluidics, RNP granules, viral genome assembly, Microbiology, Virology & Host Pathogen Interaction, Structural Biology

## Abstract

RNA viruses induce the formation of subcellular organelles that provide microenvironments conducive to their replication. Here we show that replication factories of rotaviruses represent protein‐RNA condensates that are formed via liquid–liquid phase separation of the viroplasm‐forming proteins NSP5 and rotavirus RNA chaperone NSP2. Upon mixing, these proteins readily form condensates at physiologically relevant low micromolar concentrations achieved in the cytoplasm of virus‐infected cells. Early infection stage condensates could be reversibly dissolved by 1,6‐hexanediol, as well as propylene glycol that released rotavirus transcripts from these condensates. During the early stages of infection, propylene glycol treatments reduced viral replication and phosphorylation of the condensate‐forming protein NSP5. During late infection, these condensates exhibited altered material properties and became resistant to propylene glycol, coinciding with hyperphosphorylation of NSP5. Some aspects of the assembly of cytoplasmic rotavirus replication factories mirror the formation of other ribonucleoprotein granules. Such viral RNA‐rich condensates that support replication of multi‐segmented genomes represent an attractive target for developing novel therapeutic approaches.

## Graphical abstract

Viroplasms represent protein/RNA‐rich condensates formed by phase‐separation of NSP2 and NSP5 proteins, whose disruption by chemical compounds reduces rotavirus replication.

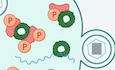

## Introduction

To reproduce successfully, RNA viruses compartmentalise their replicative enzymes within specialised organelles termed viral factories. These structures are viewed as virus assembly lines that support viral replication by sequestering and concentrating cognate nucleic acids and proteins. While most viral RNA replication requires membrane‐enclosed replication compartments, experimental evidence from recent studies (Nikolic *et al*, 2017; Heinrich *et al*, 2018; Alenquer *et al*, 2019; Guseva *et al*, 2020) suggests that liquid–liquid phase separation (LLPS) may provide a simple solution for the dynamic assembly of viral replication factories (Brangwynne *et al*, 2015; Bergeron‐Sandoval *et al*, 2016; Nott *et al*, 2016; Alberti, 2017; Wang *et al*, 2018; Alberti *et al*, 2019).

Liquid‐liquid phase separation occurs when multivalent biopolymers transiently interact to coalesce into a dense membraneless condensate (Langdon & Gladfelter, 2018; Alberti *et al*, 2019; Roden & Gladfelter, 2021). A hallmark of LLPS includes liquid‐like properties of condensate droplets formed, e.g. sphericity, fusion and fission, followed by relaxation into a sphere (Banani *et al*, 2017). This metastable state allows for rapid exchange with the surrounding cellular milieu, and it enables biomolecules within condensates to establish transient interactions (Banani *et al*, 2017). Over time, such liquid‐like condensates may form anisotropic hydrogels, fibrils and non‐fibrillar aggregates (Knowles *et al*, 2014), often associated with post‐translational modifications of the scaffold proteins that drive the LLPS of the system (King *et al*, 2012). Strong expression levels of protein scaffolds, such as those seen during viral infections, as well as multiple post‐translational modifications, e.g. phosphorylation, can drive LLPS of these proteins and their interaction clients as soon as their solubility limit is reached (Banani *et al*, 2017; Alberti *et al*, 2019). At this saturation concentration, the mixture partitions into a highly concentrated, condensed phase, in which macromolecules engage in multivalent homo‐ or heterotypic interactions that include regions of intrinsic disorder/low complexity (Banani *et al*, 2017). Structural heterogeneity of such proteins contributes to dynamic interaction networks that engage multiple partners (Brangwynne *et al*, 2015) through a plethora of diverse and transient interactions that do not confer rigid structural order (Banani *et al*, 2017). These include short‐range dipolar, π‐π or π‐cation and long‐range electrostatic interactions (Banani *et al*, 2017; Wang *et al*, 2018) that can be perturbed, e.g. by treatments with 1,6‐hexanediol, or by changing the ionic strength of the system (Banani *et al*, 2017). Similarly, these protein–protein interactions may promote ribonucleoprotein (RNP) granule formation, e.g. stress granules or P‐bodies, in which RNAs may additionally provide scaffolds for multivalent RNA‐binding proteins, connecting individual RNPs to form higher‐order assemblies (Wheeler *et al*, 2016; Van Treeck & Parker, 2018). Emerging experimental evidence suggests that such biomolecular condensates comprising multiple protein and RNAs may be organised into complex, multilayered structures (Boeynaems *et al*, 2019; Hastings & Boeynaems, 2021; Kaur *et al*, 2021).

Rotaviruses (RVs), a large group of human and animal double‐stranded RNA pathogens, replicate their genomes within cytoplasmic replication factories, termed viroplasms (Altenburg *et al*, 1980; Silvestri *et al*, 2004; Patton *et al*, 2006). These cytoplasmic inclusions can be detected by immunostaining against phosphoprotein NSP5 and/or the RNA‐binding protein NSP2 (Aponte *et al*, 1996; Fabbretti *et al*, 1999; Silvestri *et al*, 2004; Contin *et al*, 2010; Criglar *et al*, 2014; Papa *et al*, 2020a) as early as 2 h post‐infection (Fabbretti *et al*, 1999; Eichwald *et al*, 2004, 2012). Electron microscopy (EM) studies of RV‐infected cells between 8 and 24 h post‐infection revealed electron‐dense, membraneless cytoplasmic inclusions containing NSP5 and NSP2, that are often surrounded by the newly assembled double‐layered particles entering the endoplasmic reticulum (ER) via a poorly understood mechanism (Altenburg *et al*, 1980; Eichwald *et al*, 2018). The formation of early infection viroplasms requires co‐expression of the Ser/Asp/Glu‐rich acidic protein NSP5 and a positively charged RNA chaperone NSP2 (Fabbretti *et al*, 1999; Eichwald *et al*, 2004; Silvestri *et al*, 2004; Papa *et al*, 2020a) that appear to dynamically change their post‐translational modifications, e.g. phosphorylation and hyperphosphorylation of NSP5 (Poncet *et al*, 1997; Sen *et al*, 2006; Campagna *et al*, 2007; Papa *et al*, 2020a), and potentially of NSP2 (Criglar *et al*, 2018) over the course of infection. These events coincide with the viroplasmic accumulation of eleven distinct types of the RV transcripts, RNA polymerase VP1, and the core protein VP2, ultimately resulting in their correct stoichiometric co‐assembly and RNA packaging (Patton & Chen, 1999; Patton & Spencer, 2000; Silvestri *et al*, 2004; Lu *et al*, 2008; Trask *et al*, 2012). Thus, the highly dynamic nature of viroplasms likely reflects their multi‐faceted roles in supporting all stages of RV assembly, from the assortment of eleven distinct RV transcripts facilitated by NSP2 (Borodavka *et al*, 2017; Bravo *et al*, 2018; preprint: Bravo *et al,* 2021), to the final acquisition of additional protein layers to form an infectious triple‐layered particle during later stages of infection. However, the earliest events leading to the formation of NSP5/NSP2‐rich viroplasms in RV‐infected cells have remained elusive due to the lack of understanding of their nature and tools to isolate these highly dynamic cytoplasmic inclusions.

Here, we show that the assembly of rotavirus replication factories occurs via LLPS of the intrinsic disorder region (IDR)‐rich scaffold protein NSP5 and the RNA chaperone NSP2. We characterised the phase behaviour of the NSP5/NSP2 condensates and mapped out the phase boundary, at which they transition from a mixed one‐phase, to a two‐phase state. We show that at later infection (> 8–12 h) stage, viroplasms undergo a liquid‐to‐solid transition, losing their sensitivity to propylene glycol applications, which significantly reduced the virus titre when applied at concentrations sufficient to reversibly dissolve viroplasms during early infection stages.

The emerging properties of these protein–RNA condensates in a large family of dsRNA viruses are remarkably similar to those emerging from multiple studies of membraneless ribonucleoprotein (RNP) organelles, including processing (P) bodies and stress granules. Their capacity to rapidly and reversibly respond to external stimuli amounts to a shift in our understanding of rotavirus replication, providing the basis for viewing these RNA–protein condensates as an attractive target for developing novel antiviral therapeutics.

## Results

### Liquid‐like properties of rotavirus replication factories

The dynamic nature of the RNA‐rich viral cytoplasmic inclusions previously termed “viroplasms” and their tendency to coalesce (Eichwald *et al*, 2004, 2012) during rotavirus (RV) infection are reminiscent of other cytoplasmic liquid‐like ribonucleoprotein cytosolic granules (Shin & Brangwynne, 2017). Such observations have prompted us to further investigate the liquid‐like properties of viroplasms.

**Figure 1:**
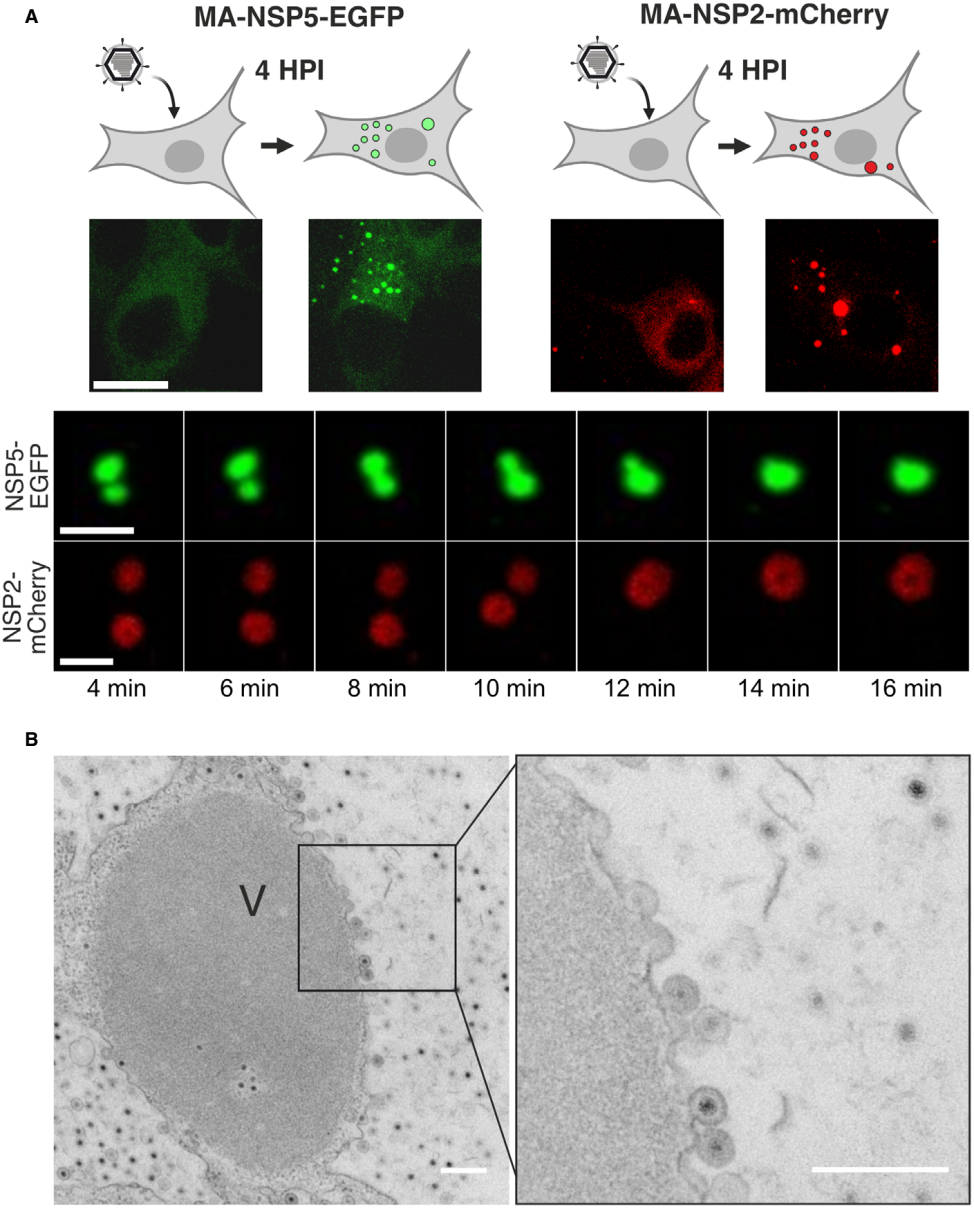
Liquid‐like properties of the rotavirus replication factories Dynamics of replication factories tagged with EGFP (NSP5‐EGFP) and mCherry (NSP2‐mCherry) visualised in MA104‐NSP5‐EGFP and MA104‐NSP2‐mCherry rotavirus‐infected cell lines. Live‐cell confocal images (4–16 min) acquired after 4 h post‐infection (HPI). Scale bars, 5 µm.Representative electron micrograph of a viroplasm (V) formed in NSP5‐EGFP cells infected with rotaviruses 8 h post‐infection. Inset—double‐layered particles emerging from the surface of a viroplasm. Scale bar = 300 nm.

Previous reports demonstrated that the two viral proteins NSP5 and NSP2 constitute the bulk of viroplasms (Berois *et al*, 2003; Eichwald *et al*, 2004; Silvestri *et al*, 2004; Taraporewala *et al*, 2006; Criglar *et al*, 2018). We used MA104 cell lines that fully support RV replication, while expressing low levels of the C‐terminally EGFP‐ and mCherry‐tagged NSP5 and NSP2, respectively (Eichwald *et al*, 2004; Papa *et al*, 2020a). In the absence of RV infection, both protein fusions remain cytoplasmically dispersed. Upon RV infection, both cytosolic NSP2‐mCherry (Papa *et al*, 2020a) and NSP5‐EGFP (Eichwald *et al*, 2012) re‐localise into newly formed replication factories containing large quantities of NSP5 and NSP2, thus making them suitable markers for live‐cell imaging of these virus‐induced organelles (Fig 1A).

At 4 h post‐infection (HPI), more than 90% of virus‐infected NSP5‐EGFP or NSP2‐mCherry‐expressing cells produced NSP5‐EGFP or NSP2‐mCherry‐containing cytoplasmic granules, respectively. We were able to observe fusion events between these granules, irrespective of the fusion fluorescent reporter protein used (Fig 1A), suggesting that these inclusions may have liquid‐like properties. Electron microscopy analysis of the NSP5‐EGFP cells infected with RVs revealed electron‐dense granules (Fig 1B), with double‐layered RNA‐containing particles emerging from their surface, as previously described for viroplasms (Altenburg *et al*, 1980; Petrie *et al*, 1984; Eichwald *et al*, 2018) further confirming that the observed cytoplasmic inclusions represent genuine viral replication factories.

**Figure 2:**
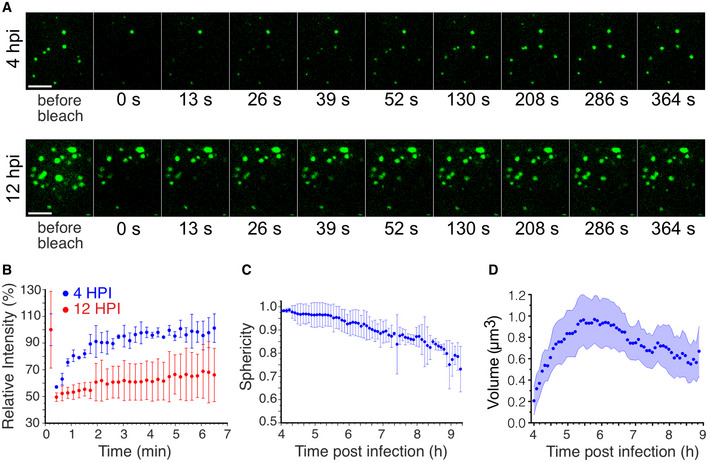
Changes in liquid‐like properties of viroplasms during the course of infection Fluorescence recovery after photobleaching (FRAP) of EGFP‐tagged replication factories after 4 HPI (early infection) and 12 HPI (late infection). Scale bar, 10 μm.Fluorescence Intensities after FRAP of EGFP‐tagged replication factories after 4 HPI (blue) and 12 HPI (red) shown in (A). Each data point represents mean ± SD intensity values calculated for multiple NSP5‐EGFP‐tagged granules in 5 RV‐infected cells.Sphericity of NSP5‐EGFP‐containing granules during RV infection. Each data point represents mean ± SD sphericity values calculated for NSP5‐EGFP‐NSP5‐tagged granules in cells detected in 15 frames. Data were recorded for 320 min immediately after 4 HPI when multiple NSP5‐EGFP granules could be detected in RV‐infected cells.Calculated volumes of NSP5‐EGFP‐tagged granules formed in RV‐infected cells after 4 HPI as shown in (C). The mean values decrease due to *de novo* formation of multiple smaller NSP5‐EGFP granules that continuously assemble in cells between 4 HPI (*t* = 0 min) and 9 HPI (*t* = 300 min). The shaded area represents the 95% confidence interval for each point. Source data are available online for this figure.

To further assess the liquid‐like state of these granules, we examined the dynamics of NSP5‐EGFP in these droplets by photobleaching viroplasms during “early” (4 HPI) and “late” (12 HPI) infection, and measuring fluorescence recovery over time (Fig 2A). Fluorescence recovery after photobleaching (FRAP) studies of the “early” viroplasms revealed a rapid (60–80 s) and complete (95–100%) fluorescence recovery. The kinetics and recovery percentage, however, decreased substantially for larger granules observed during late infection stages (Fig 2B). The reduced FRAP recovery rates of larger, less spherical viroplasms suggest changes in their material state (i.e. characterised by slower exchange rates between the dilute and dense phase of viroplasms) during late stage of infection (Alberti *et al*, 2019).

As liquid‐like properties of droplets are determined by their surface tension (Brangwynne, 2011; Bergeron‐Sandoval & Michnick, 2018), smaller liquid droplets coalesce and attain spherical shapes with the lowest volume‐to‐surface area ratios. To investigate the shape of viroplasms, we observed NSP5‐EGFP‐expressing RV‐infected cells, and we found that at 4 h post‐infection these structures are predominantly spherical (Fig 2C). Time‐resolved confocal microscopy of individual viroplasms (Materials and Methods) revealed that the overall size of droplets per cell initially increased over course of infection (Fig 2D), eventually decreasing due the constant *de novo* formation of additional smaller droplets. In contrast, the calculated sphericities of these inclusions decreased with time (Fig 2A and C), suggesting loss of fluidity, consistent with the observed slower FRAP recovery rates during late infection (Fig 2B).

**Figure 3:**
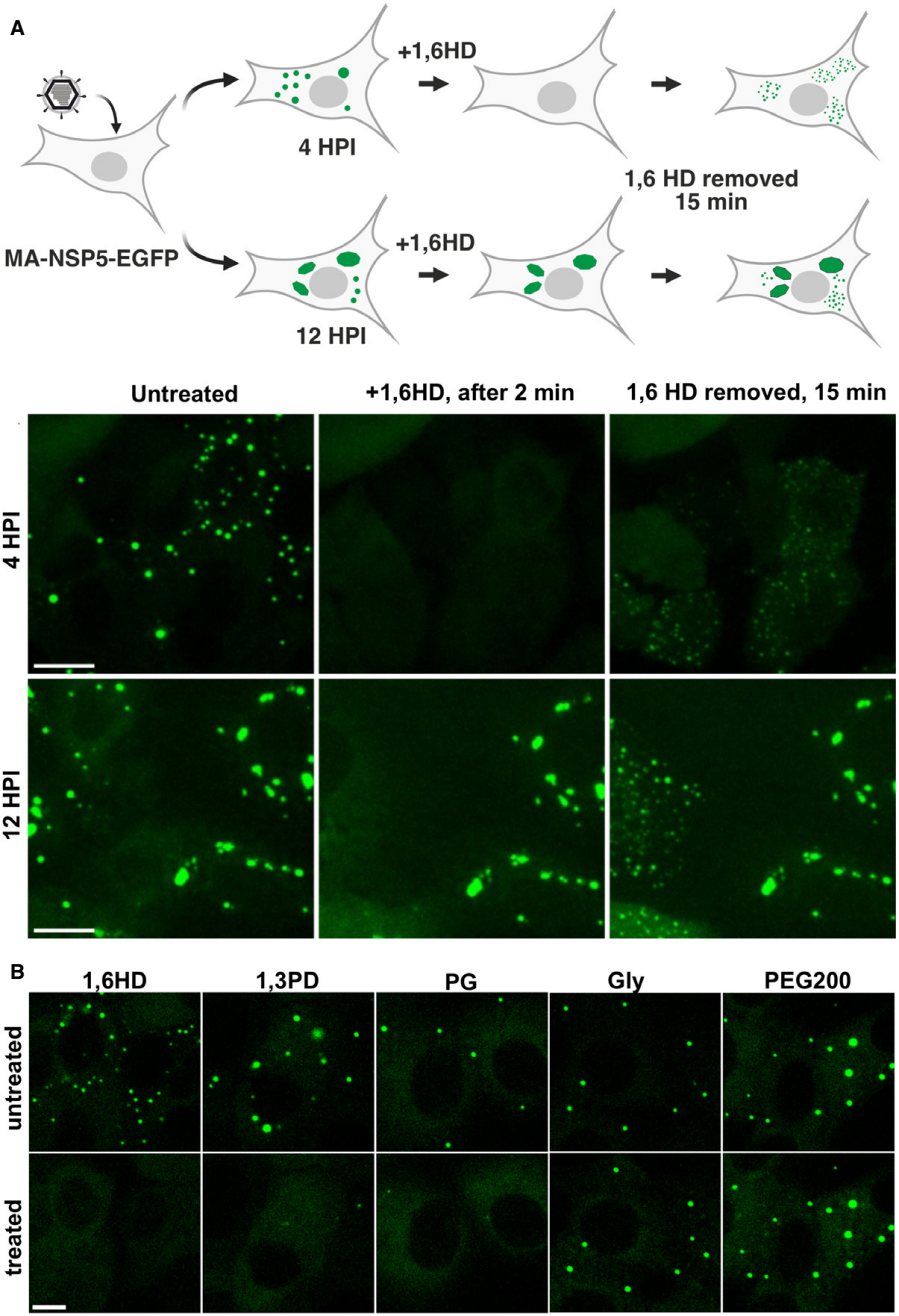
1,6‐hexanediol treatments (1,6HD) differentiate early and late viral replication factories Replication factories in MA104‐NSP5‐EGFP cells infected with RV at 4 HPI dissolve after > 30 s post‐application of 4% (v/v) 1,6HD added to the cell culture medium (middle panel). Removal of 1,6HD results reassembly of multiple EGFP‐NSP5‐containing droplets dispersed in the cytosol (right panel). Bottom, replication factories at 12 HPI: before application of 1,6HD (left), 2 min after application (middle) and 15 min after removal of 1,6HD from cell culture medium (left). Note larger viral factories that remain refractory to 1,6HD treatment. Scale bar, 50 µm.Sensitivity of RV replication factories to aliphatic alcohols at 4 HPI. Left to right—1,6‐hexanediol (1,6HD); 1,3‐propylene diol (1,3PD); 1,2‐propylene diol, or propylene glycol (PG); glycerol (Gly); polyethylene glycol 200 (PEG200). Top panels— before application and bottom panels—1 min after application of these compounds (4.5% v/v). Scale bar, 30 µm.

**Figure EV1:**
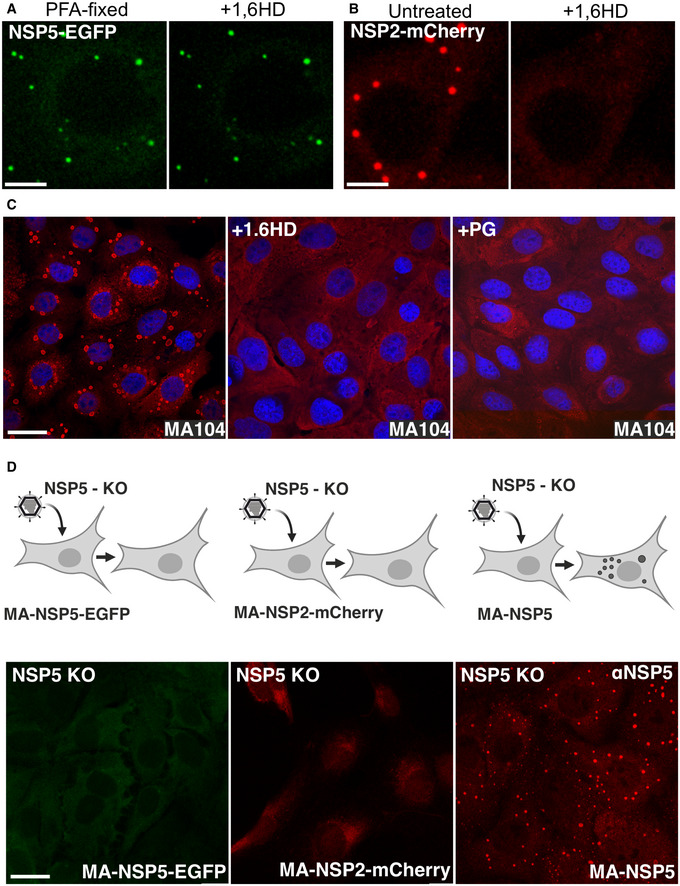
Viroplasms are sensitive to aliphatic diols during RV infection and require expression of NSP5 RV‐infected MA104‐NSP5‐EGFP cells (4 HPI) fixed with 4% (v/v) paraformaldehyde for 5 min (PFA‐fixed, left panel). Application of 1,6HD (5% v/v) does not dissolve NSP5‐EGFP granules after chemical cross‐linking with PFA (right).Live‐cell images of RV‐infected MA104‐NSP2‐mCherry cells at 4 HPI, shown in Fig 1. NSP2‐mCherry‐tagged replication factories dissolve upon application of 4% (v/v) 1,6HD (Movie EV2).Immunofluorescent (IF) staining of viral replication factories in RV‐infected MA104 cells 6 HPI, before (left) and after a brief (5 min) application of 4% 1,6HD or propylene glycol (PG), respectively, prior to PFA fixation and IF detection of NSP5 (red). Nuclei are stained with DAPI (blue).Recombinant rotavirus NSP5‐KO (NSP5 knockout) infection of MA104‐derived stable cell lines producing NSP5‐EGFP (*left*), NSP2‐mCherry (*middle*) and the wild type NSP5 (*right,* NSP5‐rich condensates, IF staining). All cells were fixed and imaged 8 h after infection with NSP5‐KO RV. Data information: Scale bar, 10 µm.

We next examined the sensitivities of both early and late viroplasms towards the aliphatic alcohol 1,6‐hexanediol (1,6HD), which is commonly used as a chemical probe to differentiate between liquid‐like and gel‐like states of membraneless organelles(Lin *et al*, 2016; Kroschwald *et al*, 2017). We exposed cells infected with rotaviruses to 4% (*v*/*v*) 1,6HD added to cell culture medium. Immediately after application of the compound (< 30 s), early infection viroplasms were completely dissolved (Fig 3A). When 1,6HD was removed, NSP5‐EGFP assemblies slowly reappeared, initially forming smaller assemblies that eventually coalesced into larger viroplasms (Fig 3A and Movie EV1). In contrast, when treated with 1,6HD at 12 HPI, only a fraction of smaller viroplasms were dissolved, while larger viroplasms remained unaffected (Fig 3A), suggesting that they have undergone maturation (Patel *et al*, 2015), consistent with the observed loss of their fluidity and slower FRAP recovery rates. A brief (5 min) chemical cross‐linking with 4% (*v*/*v*) paraformaldehyde prior to the application of the aliphatic alcohol also rendered the early infection (4 HPI) structures refractory to 1,6HD treatment (Fig EV1A). Collectively, these results suggest that the assembly of viroplasms is driven by weak hydrophobic interactions that can be stabilised by chemical cross‐linking. Additionally, we verified the 1,6HD sensitivity of viroplasms assembled in the RV‐infected cells producing NSP2‐mCherry in lieu of NSP5‐EGFP (Fig EV1 and Movies EV2 and EV3). Irrespective of the protein tagged (NSP5 or NSP2), or the fluorescent protein chosen, viroplasms responded similarly to the application of 1,6HD.

### Early infection stage replication factories are dissolved by aliphatic diols

We posited that related aliphatic diols with similar physicochemical properties to 1,6HD (e.g. hydrophobicity and molecular weight), but less toxic, might exert similar effects on viroplasms in cells. Using our cell‐based screening approach, we identified two low molecular weight aliphatic diols (1,2‐ and 1,3‐propane diols; denoted as 1,2PD and 1,3PD, respectively) that also dissolved viroplasms in RV‐infected cells at 4 HPI (Fig 3B), albeit at higher concentrations (4.5–4.7% v/v) compared to the longer chain diol 1,6HD. While 1,6‐hexanediol is toxic to cells (Kroschwald *et al*, 2017), 1,2‐propane diol, commonly known as propylene glycol (PG), is a generally recognised safe compound and is well‐tolerated by cells upon application at lower (< 5% v/v) concentrations (Mochida & Gomyoda, 1987).

Since both intracellular protein concentration and protein tagging may significantly affect the properties of the phase‐separating system (Alberti *et al*, 2019), we also carried out immunofluorescent staining of wild type MA104 cells infected with wild type RV before and after application of 1,6HD and a non‐toxic PG (Fig EV1). Both alcohols completely dissolved viroplasms, further corroborating that the observed structures are formed via LLPS of NSP5 that accumulates in the cytoplasm of RV‐infected cells.

As a final test, we used a recombinant NSP5‐deficient (knockout, KO) rotavirus (Papa *et al*, 2020a) to infect three MA104 cell lines that stably produce NSP5, NSP5‐EGFP and NSP2‐mCherry. Viroplasms were only observed in the cells producing untagged NSP5 as soon as 4–8 HPI (Fig EV1). In contrast, no viroplasms were detected in NSP2‐mCherry and NSP5‐EGFP cells, confirming that the untagged NSP5 is the key protein that drives LLPS. Together with our recent studies (Papa *et al*, 2020a), these results also suggest that C‐terminal tagging of NSP5 impairs its function and RV replication, while not precluding NSP5‐EGFP mixing with untagged NSP5/NSP2 condensates that are formed during RV infection.

Taken together, early infection stage viroplasms exhibit all the hallmarks of a liquid state: they are spherical and they coalesce; they exchange cytoplasmically dissolved proteins; they are rapidly and reversibly dissolved by a number of aliphatic alcohols that disrupt weak interactions that drive LLPS. Remarkably, aliphatic diols solubilise smaller liquid‐like viroplasms, while larger and more irregularly shaped viroplasms did not dissolve in the presence of these compounds.

### Viroplasms are formed via LLPS of nonstructural proteins NSP5 and NSP2

**Figure EV2:**
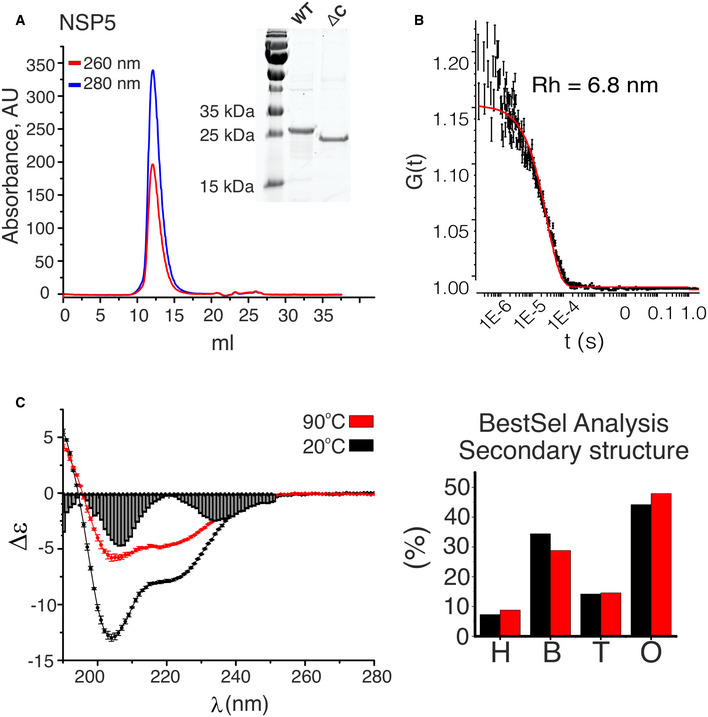
Solution characterisation of NSP5 Size‐exclusion (SEC) analysis (Superdex 200 Increase 10/300 GL) of the purified recombinant protein NSP5. After purification and refolding, the protein was monodisperse and free of nucleic acids, as judged by the A_260_/A_280_ ratio (blue trace—absorbance at 280 nm, red trace—absorbance at 260 nm). Inset—SDS–PAGE analysis of purified NSP5 and its C‐terminal truncation variant (ΔC).Quasi‐elastic scattering analysis of the SEC peak fraction shown in (A). A non‐linear fit of the data (red line) indicates an estimated hydrodynamic radius, R_h_ ˜ 6.8 nm. 5 autocorrelation functions (ACFs) were calculated for 60 s measurement per protein sample, with errors representing SD values from ACFs. Data were recorded to confirm the oligomeric state of NSP5, which is consistent with the SEC trace shown in (A).Circular dichroism (CD) spectra of NSP5 acquired at 20°C (black) and after thermal denaturation at 90°C (red). 3 scans were acquired and averaged per sample for each temperature (each point represents mean ± SD). Secondary structure analysis of NSP5 determined by spectral deconvolution of the CD spectra recorded at 20°C (black) and after the thermal denaturation (red). H—helices, B—β‐sheets, T—turns, O—disordered. BestSel fit residuals are shown for region 190–240 nm along the x‐axis. Source data are available online for this figure.

To move towards a better understanding of phase separation of viroplasm‐forming proteins NSP5 and NSP2, and to directly demonstrate their capacity to drive LLPS, we analysed their propensities to undergo LLPS *in vitro*. Previously, N‐terminal and C‐terminal tagging of NSP5 had been shown to affect formation of viroplasm‐like inclusions (Fabbretti *et al*, 1999), while C‐terminal His‐tagging of NSP2 does not affect their assembly (preprint: Bravo *et al*, 2020). We therefore examined recombinantly expressed untagged NSP5 (Fig EV2) and a C‐terminally His‐tagged NSP2 (cHis‐NSP2, see Materials and Methods). We also expressed an N‐terminally His‐tagged NSP5 (N‐His‐NSP5) for labelling purposes in order to visualise NSP5 by mixing the untagged NSP5 with the fluorescently labelled His‐tagged protein in 1:10 molar ratio (N‐His‐NSP5:NSP5), as such terminal tagging does not affect partitioning of the labelled N‐His‐NSP5 into the condensates containing NSP5/cHis‐NSP2. Circular dichroism analysis of NSP5 suggested that regions of protein disorder contributed to almost 40% of the spectrum (Fig EV2). Such intrinsically disordered regions commonly underpin LLPS of scaffold proteins (Brangwynne, 2011; Wei *et al*, 2017; Wang *et al*, 2018; Choi *et al*, 2020), commonly forming larger oligomers, in agreement with the observed hydrodynamic radius of the oligomeric NSP5 (Fig EV2).

**Figure EV3:**
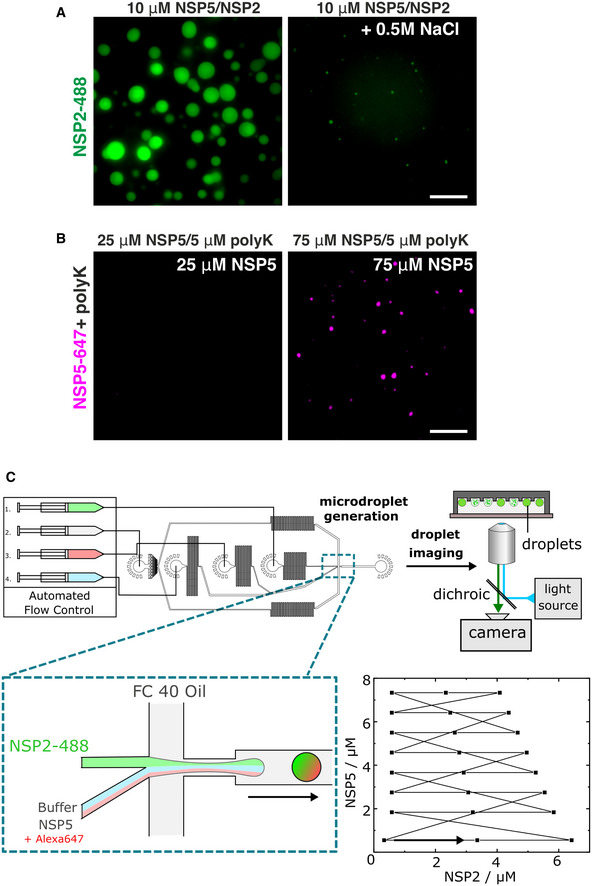
LLPS of NSP5 *in vitro* and quantitative characterisation of LLPS using PhaseScan approach NSP2‐488 + untagged NSP5 (10 μM NSP5 + 5 μM NSP2) at physiological salt concentration (*left*) and in the presence of 0.5 M NaCl (*right*). Scale bar, 10 µm.
*Left*: Untagged NSP5 (35 μM) spiked with NSP5‐647 incubated with 5 μM poly‐lysine (average MW 70 kDa, polyK); *Right*: 75 μM NSP5 + 5 μM poly‐lysine. Scale bar, 10 µm.Schematics of the droplet‐generating device. Droplets were generated using a microfluidic device controlled by automated syringe pumps. Combination of aqueous droplet components prior to the droplet‐generating junction (inset) enables variation in droplet solution composition. Droplets are collected (6 min collection time) off‐chip, before undergoing analysis by epifluorescence microscopy. Inset i—flow profile for NSP2 and NSP5 concentrations as produced by automated flow control in droplet generation. Flow set points (black squares) are maintained for 7 s, with the overall flow programme lasting 168 s. The arrow indicates the beginning of the continuous flow programme loop.

**Figure 4:**
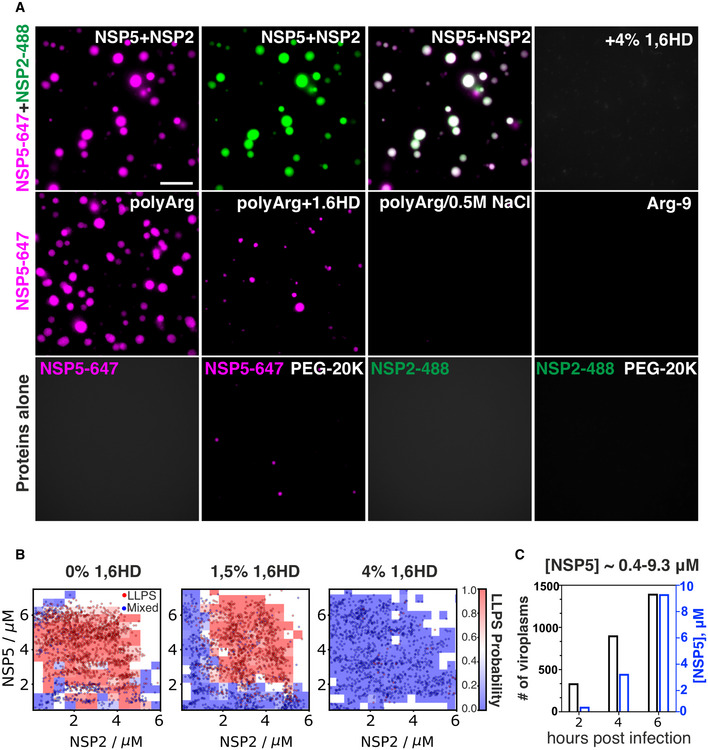
Rotavirus NSP5 and NSP2 undergo phase separation Recombinantly expressed, Atto 647‐dye‐labelled NSP5, Atto488‐dye‐labelled NSP2 and unlabelled NSP5 protein samples (see Materials and Methods) were used for investigating their phase separation properties. All labelled protein samples were mixed with the unlabelled NSP5 (1:10 molar ratio) to minimise the effect of labelling on LLPS. *Top panel*, *left to right:* NSP5‐647/NSP2‐488 droplets formed upon mixing of both proteins (10 μM each). Both channels are shown, along with an image of both channels overlaid. 4% (v/v) 1,6‐hexanediol (1,6HD) dissolves these condensates. Scale bar, 10 µm. *Middle panel, left to right:* NSP5‐647 (50 μM) + poly‐arginine (polyArg, 5 μM); NSP5‐polyArg condensate + 1,6HD; NSP5‐polyArg condensates + 0.5 M NaCl; NSP5 + R9 peptide (Arg‐9, 5 μM). *Bottom row, left to right:* NSP5‐647 sample (35 μM) alone; after addition of 10% v/v PEG‐20K; NSP2‐488 (25 μM) alone; after addition of 10% v/v PEG‐20K.Phase diagrams generated through droplet microfluidics for the coacervation of NSP2 and NSP5, in the presence of 0% v/v (*left*), 1.5% v/v (*middle*) and 4% v/v (*right*) 1,6HD. Phase diagrams were generated from *N* = 2,206, 2,035 and 1,470 data points for each 1,6‐hexanediol concentrations, respectively, and the data were used to construct the LLPS probability plots.The number of viroplasms (*black y axis on the left*) and estimated cytoplasmic NSP5 concentration ([NSP5] µM, *blue y axis on the right)* at 2, 4 and 6 h post‐infection. Viroplasms were counted in *N* = 595 ± 85 cells for each time point, and intracellular NSP5 concentration was determined by quantitative Western blotting, as described in Materials and Methods. Source data are available online for this figure.

At physiological salt concentration (PBS, or ˜ 150 mM NaCl), immediately (< 1 min) upon mixing of the untagged NSP5 protein mixed with NSP5‐647‐labelled protein with Atto488‐dye‐labelled NSP2 (5–10 µM each protein), multiple droplets were formed, containing both labelled proteins. NSP5/NSP2 condensation was salt‐dependent (inhibition at 0.5 M NaCl, Fig EV3), suggesting electrostatic contributions of charged residues in LLPS of these proteins. Remarkably, NSP5/NSP2 droplets were dissolved in the presence of 4% (v/v) 1,6HD (Fig 4A, *top panel*), confirming that these structures represented NSP5/NSP2 condensates, in agreement with dissolution of viroplasms in the presence of 4% 1,6HD *in vivo* (Movies EV1–EV3). Given the observed salt‐dependency of NSP5/NSP2 condensation, and the overall negative charge of NSP5 and the positive charge of NSP2 at physiological pH (pI ˜ 5.1–5.5 and ˜ 9 for each protein, respectively), we also examined several polycations (Boeynaems *et al*, 2019), e.g. poly‐lysine on NSP5 condensation. While 5 µM of poly‐lysine did not trigger formation of droplets even with 25 µM NSP5, multiple smaller droplets formed at 75 µM NSP5 (Fig EV3). Since lysine lacks a π cloud, we also tested poly‐arginine (polyArg) capable of contributing to cation‐π driven condensation by engaging with aromatic residues (Hastings & Boeynaems, 2021) of NSP5. Excitingly, polyArg (5 µM) was highly efficient at triggering NSP5 condensation (10 µM NSP5, Fig 4A, *middle panel*), and NSP5‐polyArg condensates were partially sensitive to 1,6HD *in vitro*. High salt concentration completely inhibited the formation of polyArg/NSP5 droplets, further indicating electrostatic contributions of the charged residues of NSP5 and polyArg in the formation of condensates. As polyvalency plays an important role in driving LLPS (Alberti *et al*, 2019), we also tested the effect of a 9‐residue arginine peptide (Arg‐9). Despite its charge and chemical composition, Arg‐9 peptide failed to promote condensation of NSP5 at similar concentration (0.2 mg/ml of the peptide corresponding to 5 µM polyArg), revealing the critical role of polyvalency in condensation of NSP5. Both proteins were homogeneous in isolation in the low micromolar (10–20 µM) concentration regime (Fig 4A, *bottom panel*). However, addition of a crowding agent (10% v/v PEG‐20K) promoted formation of NSP5 droplets, albeit at higher (> 35 µM) NSP5 concentration, revealing that NSP5 can undergo LLPS *in vitro*. In contrast, RNA chaperone NSP2 remained homogenous in solution even in the presence of PEG‐20K (Fig 4A, *bottom panel*).

**Figure EV4:**
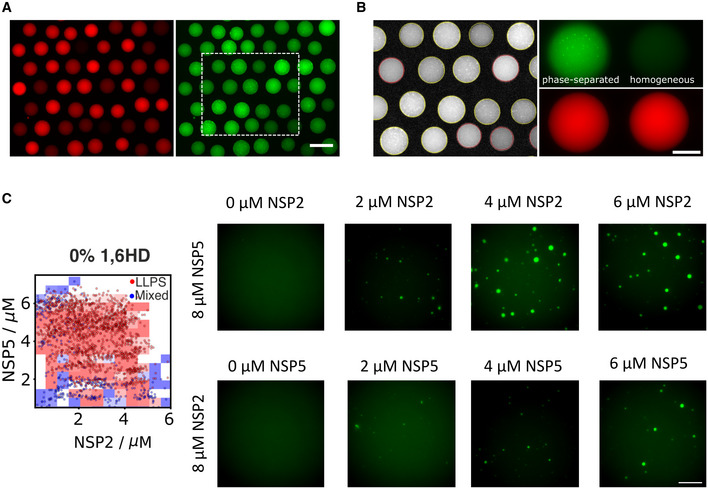
PhaseScan analysis of NSP5/NSP2 coacervates *in vitro* and quantitation of NSP5 expression during RV infection A, BRepresentative epifluorescence data for 0% 1,6‐hexanediol phase diagram of trapped microdroplets and barcode fluorescence imaged in 488 nm (*left*) and 647 nm (*right*) channels. Scale bar, 200 μm. (B) Fit of droplet outlines and phase separation classification output for region enclosed by dashed box in (A), red and yellow outlines denote droplet classification as homogeneous and phase‐separated, respectively. Representative images of microdroplets and 647‐dye‐labelled barcode fluorescence classified as phase‐separated (*left*) and homogenous (*right*) imaged in 488 nm (upper panel) and 647 nm (lower panel) channels. Scale bar, 100 μm.CPhaseScan‐generated phase diagram of the NSP5/NSP2 mix (*left panel*), with representative droplet images of NSP5/NSP2 condensates formed with 2–8 μM protein. Scale bar, 10 μm. Source data are available online for this figure.

**Figure EV5:**
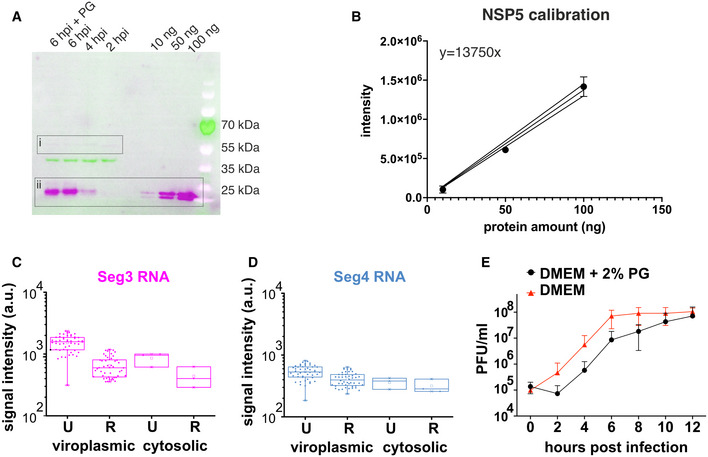
Effects of propylene glycol on rotavirus replication AWestern blot quantification of NSP5 produced in RV‐infected cells (MOI = 10) harvested at 2, 4 and 6 HPI. 1 × 10^6^ RV‐infected MA‐NSP5‐EGFP cells were harvested (see Materials and Methods), and 10% of each total cell lysate sample were loaded on a 12% SDS–PAGE gel, along with recombinant N‐His‐tagged and untagged NSP5 standards of known concentrations (10–100 ng). NSP5 was detected using anti‐NSP5 antibodies, as described in Materials and Methods. NSP5 signal (DyLight800 anti‐guinea pig, magenta, box ii) was detected simultaneously with b‐actin (hFAB Rhodamine Anti‐Actin, green) for sample loading normalisation purposes. Note a very low level of NSP5‐EGFP expression detected by NSP5‐specific antibodies (box i). The low‐level expression of NSP5‐EGFP remains constant across the infection course, while only the virally expressed NSP5 levels increase between 2 and 6 HPI. NSP5 amount in RV‐infected cells treated with 4.7% propylene glycol (“6 hpi + PG,” 15 min treatment) is similar to that produced at 6 HPI in untreated cells.BQuantification of the Western blot data shown in (A). Integrated band intensities for NSP5 samples of known concentrations were determined using Chemidoc MP Imaging system and plotted as mean ± SD values. Linear regression analysis fit (solid black line) shown along with 95% CI (solid lines) was used to determine the amount of NSP5 produced at 2, 4 and 6 HPI. Quantification for each point was carried out three times, each representing technical replicates.C, DChanges in the localisation of Seg3 and Seg4 RNAs and their relative distribution between the viroplasms and the cytosol before (Untreated, U) and 15 min after PG treatment (Recovery, R). Median and quartile values of integrated signal intensities (normalised by area) for each channel for viroplasms 2 (“viroplasmic”), and individual cells (*N* = 9, “cytosolic”) are shown, data represent technical replicates (individual RV‐infected cells). Box plots represent the 25^th^/75^th^ interquartile range, with whiskers representing the 5^th^/95^th^ percentile values. Medians shown as central bands, and means shown as squares. Crosses denote 1% and 99% percentile values, and minimum and maximum values are shown as dashes.ERotavirus replication kinetics (strain SA11) in MA104 cells adapted to grow in the presence of 2% PG. Infection was carried out in the presence of 2% PG (black) and a standard PG‐free medium (red). 2% PG was added 1 h after virus absorption (MOI of 10). Virus titres are expressed in PFU/ml, and each measurement represents a mean ± SD values estimated for three independent repeats. Source data are available online for this figure.

To further characterise the phase behaviour of NSP5/NSP2 condensates, we generated phase diagrams for these protein mixtures alone and in the presence of 1,6HD. Using high‐throughput droplet microfluidics (Fig EV3), we obtained phase diagrams for a range of NSP5 and NSP2 concentrations (Figs 4B, EV3 and EV4), revealing coacervation of the proteins occurred in the low micromolar regime. NSP5/NSP2 protein mixtures remained homogenous in the presence of 4% (*v*/*v*) 1,6HD, with a detectable change in the phase separation behaviour observed even at lower 1,5% (*v*/*v*) 1,6HD concentration, consistent with the observed instant solubilisation of viroplasms in cells the presence 1,6HD. Quantitative NSP5 expression analysis of RV‐infected cells at 2, 4 and 6 HPI reveal that during the RV infection (Materials and Methods), intracellular NSP5 concentration varies between ˜ 0.4–10 µM (Figs 4C and EV5). Thus, the observed LLPS of NSP5 and NSP2 occurring in the low micromolar regime *in vitro* recapitulates low µM concentrations of NSP5 required for viroplasm formation *in vivo,* further providing strong evidence for LLPS‐driven formation of viroplasms (Alberti *et al*, 2019).

**Figure 5:**
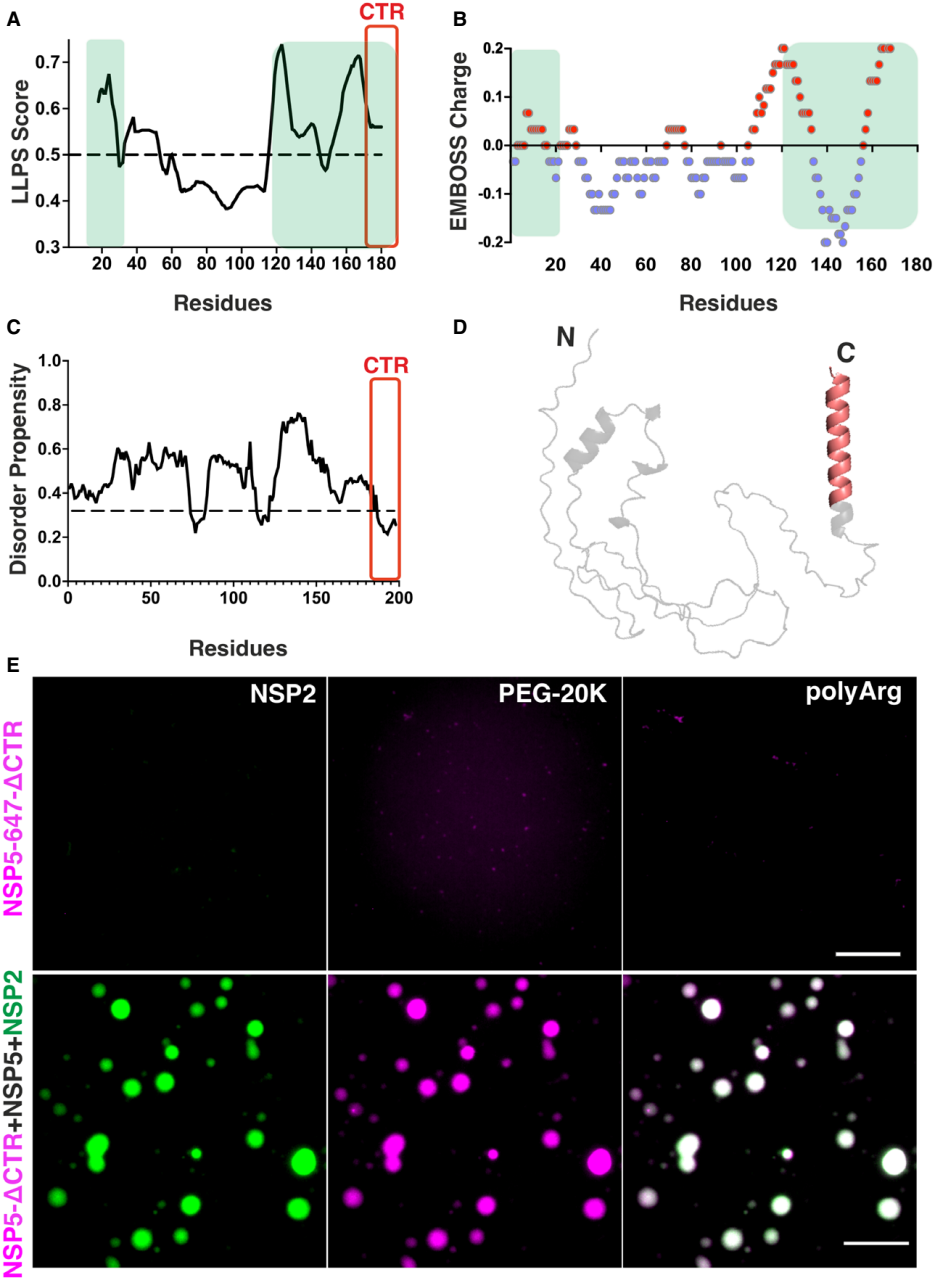
NSP5 drives of liquid–liquid phase separation required for viroplasm formation DeePhase analysis of the phase‐separating properties of NSP5 (regions with a score of > 0.5, i.e. above the dotted line, indicate residues with high LLPS potential). CTR (amino acid residues 179–197) is highlighted in red. Note that the amino acid numbering represents an average score with a 30‐residue sliding window, and not individual residues. Green boxes highlight NSP5 regions previously shown to be essential for viroplasm‐like structure formation when co‐expressed with NSP2 (Eichwald *et al*, 2004).EMBOSS protein charge plot shown as an alternating blue (negatively charged) and red (positively charged) regions with a 30‐residue sliding window.flDPnn Disorder Propensity Plot of NSP5. Regions with a score above the dotted line are predicted to be disordered.NSP5 model predicted using Alphafold2. Region 179–197 of the CTR within the predicted C‐terminal alpha‐helix is highlighted in red. (E) *Top panel, left to right:* NSP5‐ΔCTR labelled with Atto 647 (25 μM) incubated with unlabelled NSP2 (10 μM); NSP5‐ΔCTR in the presence of 10% v/v PEG‐20K; and with 5 μM of poly‐arginine (polyArg). *Bottom panel:* Atto 647‐labelled NSP5‐ΔCTR (5 μM) incubated with unlabelled NSP5 (25 μM) and Atto 488‐labelled NSP2 (10 μM). NSP5/NSP2 droplets containing labelled NSP2 (green) also contain NSP5‐ΔCTR‐Atto 647 (magenta), shown along with an image of both 488/647 channels overlaid. Scale bar, 10 µm. Source data are available online for this figure.

To dissect the sequence features of NSP5 that drive its phase separation, we employed our recently developed machine learning approach termed DeePhase (Saar *et al*, 2021) to identify the LLPS‐prone regions. The overall DeePhase score of 0.61 indicated that NSP5 meets the criteria of a phase‐separating protein, i.e. DeePhase score of > 0.5. In contrast, the global DeePhase score of NSP2 of 0.2 suggested that this RNA chaperone has low propensity to drive phase separation. Further sequence analysis of NSP5 with a moving average of 30 amino acid residues revealed several regions with high propensity to drive phase separation, i.e. LLPS score > 0.5 (Fig 5A). Remarkably, these LLPS‐prone regions overlapped with the two sections of NSP5 previously shown to be crucial for viroplasm‐like structure assembly with NSP2 (Eichwald *et al*, 2004; Fig 5A, regions highlighted in green). One of these regions contained multiple negatively charged residues (Fig 5B, C‐terminal negatively charged residues shown in blue), previously proposed to interact with the surface‐exposed positively charged residues of NSP2 (Jiang *et al*, 2006). Given the opposite charges of these proteins, and the observed ionic strength‐dependent inhibition of their phase separation (Figs 4A and EV3), NSP5/NSP2 coacervation is likely driven by electrostatic, cation‐π, and hydrophobic interactions that are sensitive to aliphatic diols, e.g. 1,6HD.

To further characterise disordered regions of NSP5, we carried out *in silico* analyses using the recently developed predictor of protein disorder flDPnn (Hu *et al*, 2021), whose predictions have recently outperformed most existing tools based on the recent Critical Assessment of Intrinsic Disorder (CAID) prediction. FlDPnn revealed that NSP5 has high propensity for disorder, in agreement with our CD spectral analysis (Fig EV2). While previous attempts to obtain high‐resolution diffraction data for NSP5 were unsuccessful, we also took advantage of the recently developed neural network‐based AlphaFold2 structure modelling approach (Jumper *et al*, 2021). Excitingly, all NSP5 structure models generated by Alphafold2 contained an extended C‐terminal helix located within the C‐terminal region (CTR), previously shown to be responsible for NSP5 oligomerisation (Fig 5D, highlighted in red). Since oligomerisation of scaffold proteins contributes to the multivalency of weak interactions driving LLPS, it is commonly associated with phase separation (Brangwynne *et al*, 2015; Banani *et al*, 2017; Shin & Brangwynne, 2017; Alberti *et al*, 2019). We therefore examined the phase separation behaviour of the C‐terminal truncation mutant of NSP5 (NSP5‐ΔCTR) that lacks the last 18 amino acid residues (Materials and Methods and Fig. EV2), failing to form decamers in solution (Martin *et al*, 2011). This mutant exists as a mixture of dimers and monomers that retain their capacity to interact with NSP2, yet incapable of forming viral inclusions (Martin *et al*, 2011) resulting in abrogation of rotavirus replication (Papa *et al*, 2020a). In contrast to its full‐length counterpart, NSP5‐ΔCTR did not form NSP5/NSP2 droplets *in vitro*. Despite retaining its C‐terminal negatively charged residues, this mutant also failed to form droplets in the presence of poly‐arginine or PEG‐20K (Fig 5E, *upper panel*). Crucially, non‐oligomerising NSP5‐ΔCTR retained its capacity to partition into the preformed full‐length NSP5/NSP2 condensates (Fig 5E, *bottom panel*), revealing the essential role of the CTR for phase separation of NSP5, irrespective of its heterotypic interactions with NSP2.

### Early viroplasms are biomolecular condensates enriched in RNAs

**Figure 6:**
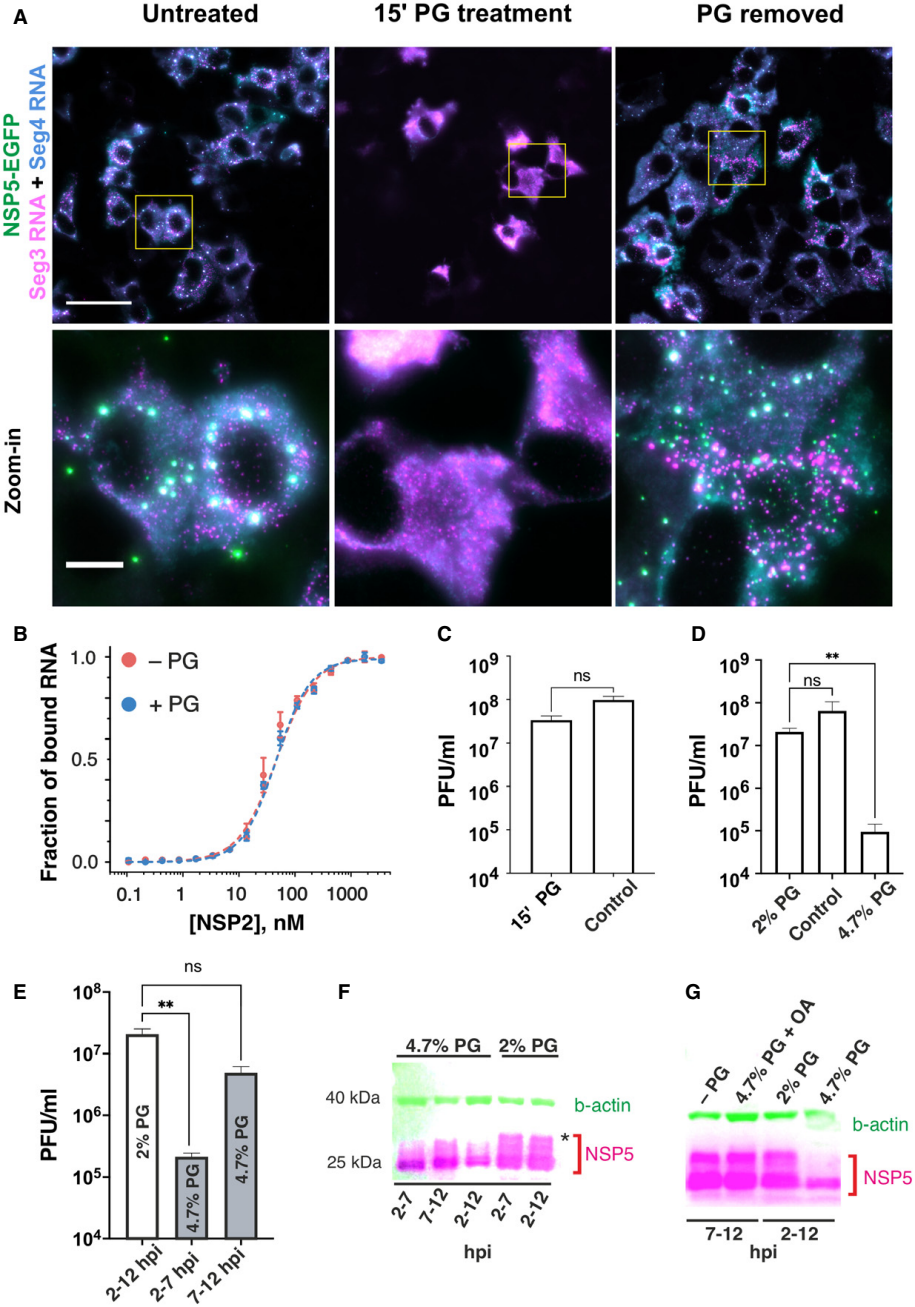
Rotavirus replication factories are RNA‐protein condensates sensitive to propylene glycol ARV‐infected MA‐NSP5‐EGFP cells at 6 HPI. NSP5‐EGFP‐tagged viral factories (green) are dissolved in the presence of 4.7% (v/v) propylene glycol (PG, *middle*). Viral RNA‐protein condensates rapidly reform (< 10 min) after replacing the PG‐containing cell culture medium (PG removed, *right*). Seg3 (magenta) and Seg4 (cyan) transcripts are detected by smFISH, and colocalising Seg3 and Seg4 RNA signals (white). Scale bars: 50 µm, zoomed‐in regions: 10 µm.BBinding of NSP2 to a fluorescently labelled 20‐mer ssRNA in the presence of 4.7% propylene glycol (PG), measured by fluorescence anisotropy. Data points represent mean anisotropy values ± SD from three measurements (technical replicates, values normalised from 0 to 1).C–EEffects of PG treatments on the viral production. PG‐containing medium (2% or 4.7% PG), or standard cell culture medium, were applied to RV‐infected cells (MOI of 10) 2 HPI (1 h after virus absorption). (C) Viral titres (PFU/ml) of samples harvested at 11 HPI that were briefly (15 min) treated with 4.7% PG at 4 HPI (15' PG), and those of untreated RV‐infected cells harvested at 11 HPI (control). Virus titres are expressed as mean ± SD values estimated for four independent repeats and were compared by a two‐tailed Mann‐Whitney test (no significant difference, *P* = 0.0857). (D) Rotavirus titres measured after 10 h of PG treatments. The 2% PG treatment slightly lowered the titre compared to the control group (ns, *P* = 0.2282), while the 4.7% PG treatment significantly inhibited viral replication (*P* = 0.0032). Reported viral titres represent mean ± SD values estimated for four independent biological replicates. (E) Effects of PG treatments on virus production at different infection points. 4.7% (v/v) PG was applied between 2 and 7 HPI (“early infection”) and then diluted to 2% between 7 and 11 HPI, or 4.7% PG was applied between 7 and 11 HPI (“late infection”) and diluted prior to harvesting the virus. Each group was compared to the control group (10 h treatment with 2% PG). Each group was compared to the control group (10 h treatment with 2% PG). Application of 4.7% PG for 5 h significantly reduced virus replication (*P* = 0.0016) between 2 and 7 HPI compared to the control group. PG treatment between 7 and 12 HPI did not significantly reduce the viral titre compared to the control group (*P* = 0.1141). Infectious titres represent mean ± SD values estimated for four independent biological replicates. Statistical analyses were performed using a Kruskal–Wallis test, followed by uncorrected Dunn’s multiple comparisons test (**P < 0.002).F, G4.7% PG treatment of RV‐infected cells results in NSP5 dephosphorylation. (F) Western blot analysis of RV‐infected cells harvested at 7 and 12 HPI, treated with 2% or 4.7% PG at different infection time points indicated in the figure. Multiple phosphorylated forms of NSP5 can be seen as higher MW bands. Treatment with 4.7% PG reduces phosphorylation, which is not perturbed in the presence of 2% PG. Treatments administered between 2 and 7 and 7–12 hpi reduce NSP5 phosphorylation, albeit to a considerably lower degree compared to the 2–12 hpi treatment. (G) Okadaic acid (5 μM) applied to 4.7% PG‐treated infected cells blocks NSP5 dephosphorylation restoring its phosphorylation pattern. Source data are available online for this figure.

Given that viroplasms are viewed as sites of viral replication (Silvestri *et al*, 2004; Patton *et al*, 2006) that accumulate rotavirus transcripts where they may be remodelled by the RNA chaperone NSP2 (Borodavka *et al*, 2017; Bravo *et al*, 2018; preprint: Bravo *et al,* 2020), we examined how solubilisation of NSP5/NSP2 condensates would affect their RNA composition *in vivo*. smFISH analysis of the RV genomic segment 3 (Seg3) and segment 4 (Seg4) transcripts in MA104‐NSP5‐EGFP cells confirmed that viroplasms contained both RNAs at 4 HPI (Fig 6A, *left*). Treatment of RV‐infected cells with 4.7% propylene glycol (PG) resulted in rapid disassembly of the RNA‐rich NSP5/NSP2 granules and re‐localisation of the RV transcripts into the cytoplasm (Fig 6A, *middle*). Removal of PG, followed by 15 min recovery prior to fixing cells and carrying out smFISH, revealed reassembly of smaller NSP5‐EGFP granules containing both Seg3 and Seg4 transcripts. Integrated intensity analysis of the RNA signals before and 15 min post‐recovery after PG treatment suggested that Seg3 and Seg4 RNA transcripts remained intact upon viroplasm dissociation, consistent with rapid (15 min) reformation of multiple RNA‐rich granules when PG was removed from cell culture medium (Fig. EV5). A fraction of viral transcripts formed RNA clusters outside NSP5‐EGFP granules (Fig 6A), also after PG treatment, suggesting that viral transcripts assemble independently of the ability of NSP5 and NSP2 to form condensates. Moreover, after PG recovery, not all NSP5/NSP2 condensates were equally enriched in RNAs, further corroborating viral RNA re‐distribution and exchange between these granules (Fig. EV5). Our recent studies indicate that rotavirus RNA oligomerisation requires NSP2 (Borodavka *et al*, 2017, 2018). The apparent affinity of NSP2 for RNA was identical in the presence of 4.7% PG (Fig 6B), confirming that addition of PG does not perturb the assembly of NSP2–RNA complexes.

To further gain insights into the functional role of LLPS in viroplasm formation, we analysed RV replication in cells treated with 4.7% PG. We noticed that the apparent viability of MA104 cells treated with PG concentrations above 4% (v/v) in cell culture medium was compromised when cells were incubated for > 2 h. We therefore initially examined viral replication in cells only briefly exposed to 4.7% PG at 4 HPI, i.e., when viroplasms are highly abundant and remain sensitive to PG. Surprisingly, despite complete dissolution of viroplasms (Fig 6A), the overall viral titres measured 8 h post‐exposure were not significantly affected (Fig 6C). Since viroplasms rapidly reformed in the absence of PG (Fig 6A), we concluded that the 15 min treatment was not sufficient to cause a significant drop in viral replication. Since propylene glycol is generally recognised as safe, and it was previously reported to be tolerated by various cells at concentrations up to 7% (v/v) in cell culture media (Mochida & Gomyoda, 1987), we attributed the observed effect to the hyperosmolarity of the PG‐containing medium. To be able to carry out longer PG treatments, we subjected MA104 cells to hyperosmotic loading with 2% PG added to cell culture medium (Materials and Methods), in order to improve their passive volumetric recovery exhibited under osmotic stress (Albro *et al*, 2009). After at least three consecutive passages of cells under these hyperosmotic conditions (800 mOsm/kg), PG‐adapted MA104 cells were infected with RVs (MOI = 10) for 1 h, followed by removal of the unabsorbed virus. The infection was continued for 1 h prior to the application of PG for specified periods of time, after which the virus was harvested for quantification at 12 HPI. Remarkably, there was no significant difference in the viral titres at 12 HPI under 2% PG conditions compared to the PG‐free control group (Fig 6D). Replication kinetics data revealed that in the presence of 2% PG, the virus replicated slower, yet reaching similar titres as the control group between 10 and 12 HPI (Fig. EV5). In contrast, application of 4.7% PG resulted in significant > 200‐fold viral titre reduction (Fig 6D) compared to the 2% PG group, and ˜ 800‐fold reduction compared to when PG was omitted. Since only early infection, liquid‐like viroplasms are sensitive to PG, we also investigated whether the timing of PG application is important for reducing the viral replication. Remarkably, early application of PG (2–7 HPI) had significantly more profound impact on RV replication (Fig 6E) compared to PG application during later stage (7–12 HPI).

Since NSP5 phosphorylation is dependent on co‐expression of NSP2 (Fabbretti *et al*, 1999; Sen *et al*, 2006; Papa *et al*, 2020a) and is crucial for RV replication, we then investigated whether disruption of NSP5/NSP2 condensates would have impact on its phosphorylation *in vivo*. Treatments with 4.7% PG resulted in the apparent reduction of NSP5 hyperphosphorylation, notably when cells were treated between 2 and 12 h. We also noted the reduction of NSP5 levels when cells were treated with 4.7% PG between 2 and 12 h, in agreement with the observed reduction of viral titres. In contrast, 2% PG treatments did not reduce NSP5 hyperphosphorylation (Fig 6F, higher MW bands corresponding to multiple phosphorylation forms of NSP5**)**. Remarkably, inhibition of cytosolic phosphatases by okadaic acid restored hyperphosphorylation of NSP5 in the presence of 4.7% PG (Fig 6G) to the levels comparable to the untreated or 2% PG‐treated cells.

**Figure 7:**
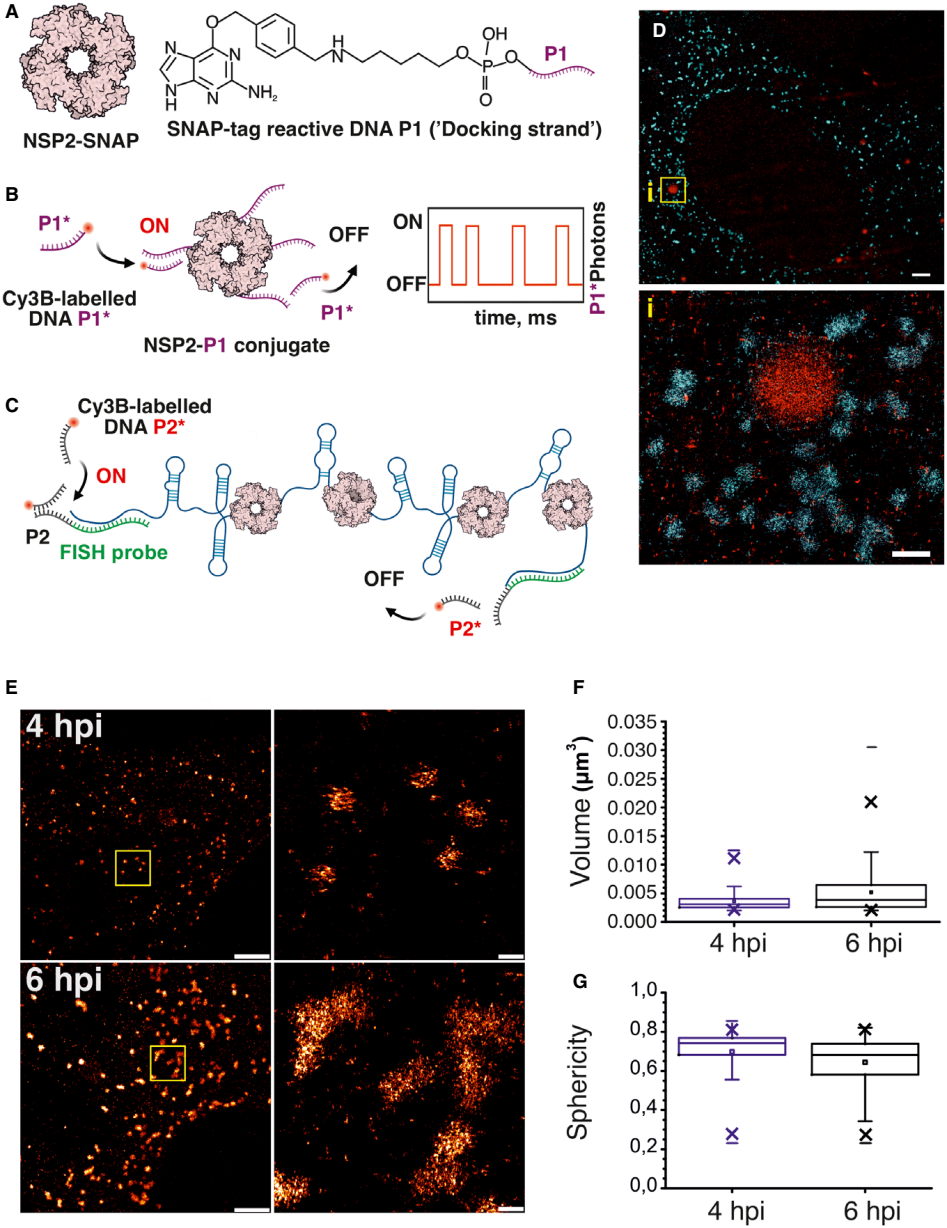
Super‐resolution DNA‐PAINT analysis of RV replication factories ADNA‐labelling scheme of NSP2 for DNA‐PAINT imaging. Low levels of SNAP‐tagged NSP2 (a doughnut‐shaped NSP2 octamer) are produced in a stable MA104 cell line. A SNAP tag‐reactive benzylguanine (BG) DNA derivative (P1 ssDNA docking strand) can form a stable thioether bond with NSP2‐SNAP.BDetection of NSP2‐rich condensates formed in RV‐infected cells using DNA‐PAINT approach. Transient binding and dissociation of a Cy3B‐dye‐labelled ssDNA probe P1* (complementary to ssDNA P1) generates blinking at the target sites (ON/OFF) used for stochastic super‐resolution imaging.CSchematics of smFISH/DNA‐PAINT approach using orthogonal P2 ssDNA docking sites installed into Seg3‐specific RNA FISH probes.DA combined super‐resolved image of NSP2‐rich condensates (NSP2‐SNAP in red) and Seg3 transcripts (cyan) in RV‐infected cells 4 HPI. Scale bars = 2 μm (*top*), 500 nm (*inset i, bottom*).E3D DNA‐PAINT analysis of Seg3 RNA foci in RV‐infected cells at 4 and 6 HPI. Scale bars, 2 µm (left) and 200 nm (zoomed‐in, right).F, GDistribution of calculated volumes and sphericities of the Seg3 RNA‐containing granules in RV‐infected cells at 4 HPI (*N* = 704) and 6 hpi (*N* = 698), shown in (E). Box plots represent the 25^th^/75^th^ interquartile range, with whiskers representing the 5^th^/95^th^ percentile values. Medians shown as central bands, and means shown as squares. Symbol “x” denotes 1% and 99% percentile values, and min and max values are shown as “‐.” At the 0.001 level, the two distributions are significantly different between 4 and 6 hpi, assessed by the two‐sample Kolmogorov‐Smirnov test. Source data are available online for this figure.

Despite the observed perturbation of the NSP5/NSP2 condensates with aliphatic diols, our results suggest that NSP2–RNA complexes did not dissociate under those conditions (Fig 6A and B). This aspect of viroplasm formation remarkably resembles the formation of other complex ribonucleoprotein condensates, e.g. paraspeckles, in which RNA foci did not dissociate in the presence of aliphatic diols, despite the apparent dissolution of paraspeckles (Yamazaki *et al*, 2018). We therefore characterised the RNA foci formed in RV‐infected cells during early infection using super‐resolution DNA‐PAINT approach (Dai *et al*, 2016) combined with smFISH. This super‐resolution technique exploits transient binding of fluorescent DNA probes (“imagers”) to complementary, RNA‐bound “docking” DNA strands (Fig 7A–C). At 4 HPI, Seg3 transcripts could be detected as submicron‐sized RNA clusters (Fig 7D), similar to those seen in diffraction‐limited images (Fig 6A). 3D DNA‐PAINT imaging of NSP2 condensates in RV‐infected cells confirmed that early infection condensates contain only few viral transcripts, suggesting that NSP5/NSP2 coacervation spontaneously occurs during early RV infection, and it is not nucleated by the transcribing viral particles present in cells.

Furthermore, 3D DNA‐PAINT imaging of Seg3 RNA foci revealed that as they increased, they became less spherical by 6 HPI (Fig 7E–G), reflecting the overall decrease in sphericity of viroplasms during late infection. Given the refractivity of the RNA foci to aliphatic diols, and rapid (10–15 min) reformation of smaller condensates upon removal of these compounds, it is possible that such viral RNA aggregates could seed the nucleation of new NSP2/NSP5 condensates in cells (Garcia‐Jove Navarro *et al*, 2019).

Taken together, these results confirm that early infection stage viroplasms should be viewed as specialised liquid‐like RNA‐protein granules (Lin *et al*, 2015; Khong *et al*, 2017; Van Treeck & Parker, 2018; Rhine *et al*, 2020) that support replication of a multi‐segmented RNA genome of rotaviruses.

## Discussion

Biomolecular condensates have been shown to contain hundreds of distinct molecular species (Ditlev *et al*, 2018), acting as membraneless protein‐rich liquid condensates that selectively enrich biomolecules and can promote nucleic acid remodelling within (Nott *et al*, 2016). Despite complex and dynamic composition of condensates, typically, only one or few proteins are required to form them (Ditlev *et al*, 2018; Langdon & Gladfelter, 2018; Wang *et al*, 2018; Alberti *et al*, 2019). Previous studies revealed protein composition of viroplasms, suggesting that these cytoplasmic inclusions are formed when NSP5 is co‐expressed with NSP2 (Fabbretti *et al*, 1999; Eichwald *et al*, 2004; Contin *et al*, 2010; Papa *et al*, 2020a) and/or the viral capsid protein VP2, even in the absence of RV infection (Eichwald *et al*, 2004; Contin *et al*, 2010; Criglar *et al*, 2014, 2018; Buttafuoco *et al*, 2020). Here, we demonstrate that rotavirus viroplasms represent condensates that are formed via phase separation of NSP5 and NSP2. During early infection, NSP5/NSP2‐rich inclusions are spherical, they fuse and relax into a sphere. Using recombinantly expressed proteins NSP5/NSP2, we show that both proteins undergo rapid condensation upon mixing at physiologically relevant, low μM concentrations. These NSP5/NSP2 droplets were sensitive to a range of aliphatic diols known to disrupt interactions that drive LLPS. Our discovery that several aliphatic diols other than 1,6HD can reversibly dissolve viroplasms in RV‐infected cells reinforces the idea that these inclusions represent condensates.

Given multiple lines of evidence demonstrating its indispensable role in the formation of viroplasms (Poncet *et al*, 1997; Fabbretti *et al*, 1999; Mohan *et al*, 2003; Eichwald *et al*, 2004; Papa *et al*, 2020a), we propose that NSP5 acts as a scaffold for LLPS. Our DeePhase analysis of NSP5 and NSP2 reveals that globally, NSP5 has high propensity to form condensates, whereas its client NSP2 does not. Knocking out NSP5 abolishes formation of these structures even when other viral proteins are present during infection (Papa *et al*, 2020a; Fig EV1), while NSP5 co‐expression with RV multivalent RNA‐binding proteins, e.g. NSP2 (Berois *et al*, 2003; Eichwald *et al*, 2004), results in formation of such condensates. Our *in vitro* data fully corroborate this idea, as only NSP5 can form condensates in the presence of charged polymers and crowding agents, whereas NSP2 does not. Moreover, we have shown that the C‐terminal region of NSP5 required for its oligomerisation is indispensable for LLPS of NSP5, irrespective of NSP2. Furthermore, we have shown that NSP5/NSP2 condensate formation does not require NSP5 phosphorylation, consistent with recent observations that NSP5/NSP2 RNA‐containing viroplasms are formed by the phosphorylation‐deficient S67A mutant during infection (Papa *et al*, 2020a). Interestingly, during late infection stages, S67A mutant produced large aberrant aggregates, suggesting that although NSP5 phosphorylation is not required for its condensation and viroplasm formation, it must play role in regulating their maturation as their protein and RNA composition dynamically change during infection. Indeed, phosphorylation of condensate scaffolds that result in changes of their material properties, or assembly pathways, is a common theme for many membraneless organelles formed *in cellulo* and *in vitro* (Bah *et al*, 2015; Aumiller & Keating, 2016).

Remarkably, NSP5 is also capable of efficient condensation in the presence of poly‐arginine. Given multiple surface‐exposed arginine residues of NSP2, and N‐terminal Arg‐rich motifs of VP2, our observations offer interesting insights into why NSP5 may readily form droplets with both NSP2 and VP2. Both NSP2 and VP2 are multivalent, arginine‐rich proteins. Condensation of NSP5 occurs in the presence of low μM concentrations of poly‐arginine, whereas shorter arginine peptides were insufficient to produce droplets, suggesting multivalency is a pre‐requisite for condensation of NSP5. However, since VP2 is notoriously difficult to maintain homogeneous in solution, in this study we only focussed on investigating the physicochemical properties of condensates formed by NSP5 and its major binding client NSP2.

Given the multivalent RNA‐ and NSP5‐binding nature of NSP2, the observed phase separation of these proteins at low micromolar concentration is consistent with previous reports of their aggregation‐prone behaviour at higher micromolar concentrations (Jiang *et al*, 2006; Borodavka *et al*, 2017). By exploring the phase boundary using PhaseScan, we have shown that the degree of NSP5/NSP2 coacervation is determined by the concentrations of both interacting partners. Thus, our model predicts that the formation of discrete NSP5/NSP2 droplets should depend on intracellular concentration of both proteins, the expression of which directly correlates with the multiplicity of infection. Indeed, previous observations (Carrẽo‐Torres *et al*, 2010) are entirely consistent with our model, and we have shown that the number of viroplasms increases in accord with NSP5 concentration during RV infection (Fig 4C).

**Figure 8:**
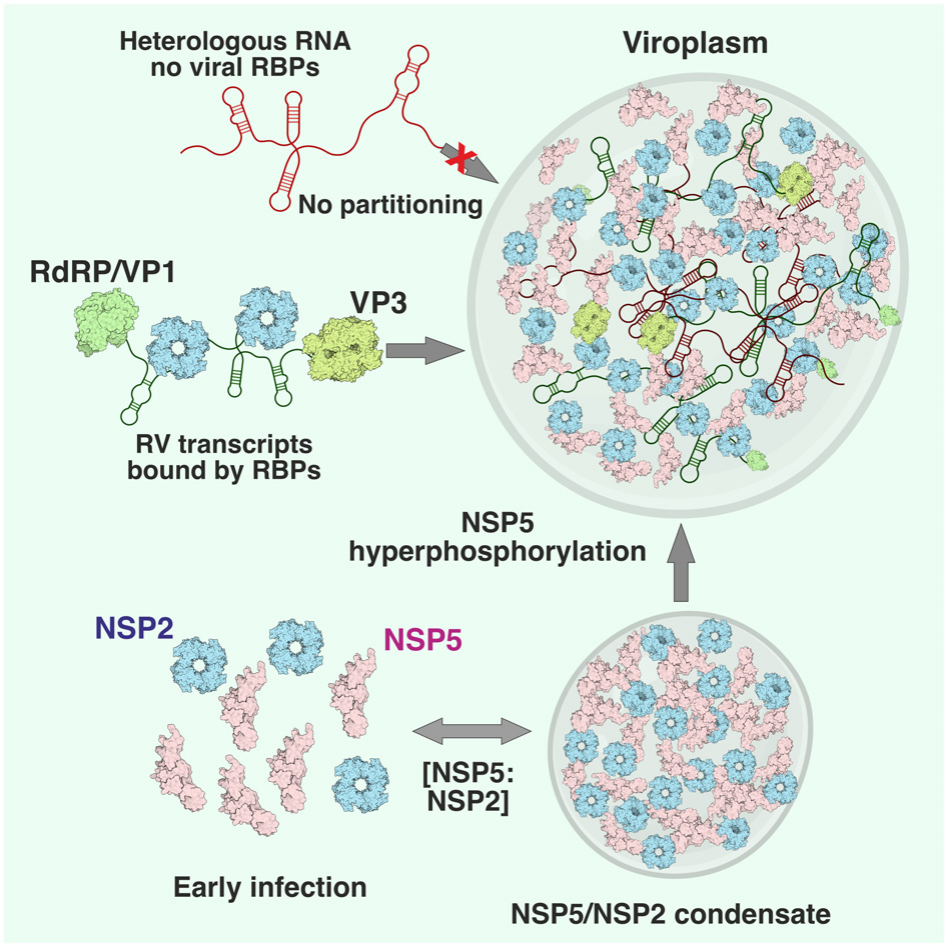
Proposed model of LLPS‐driven formation of viral replication factories in rotaviruses Multivalent Asp/Glu‐ and Ser‐rich protein NSP5 (pink) is a scaffold that recruits RNA chaperone NSP2 (cyan doughnut‐shaped octamers), and other RNA‐binding clients. NSP5 and NSP2 undergo coacervation at low micromolar concentrations, forming protein droplets, also known as “viroplasm‐like structures.” RV transcripts undergo enrichment in these condensates via a mechanism distinct from other better characterised RNP granules. Mechanistically, this could be achieved via a specific protein‐RNA recognition, e.g. binding of the RNA‐dependent RNA Polymerase (RdRP) VP1 that recognises a conserved sequence present in all RV transcripts. Such RNP complexes are then absorbed into the NSP5/NSP2 condensates consistent with low nM affinity of VP1 for both NSP5 and NSP2 (Arnoldi *et al*, 2007; Viskovska *et al*, 2014). Other similarly sized, non‐viral transcripts (red), devoid of these proteins do not partition into viroplasms. Other multivalent RNA‐binding proteins (RBPs), i.e. viral capping enzyme (Pizarro *et al*, 1991), and NSP2 can also assist in partitioning of the RNP complexes into the NSP2/NSP5 condensates, which can be dissolved with aliphatic diols. Upon NSP5/NSP2 condensation, NSP5 undergoes excessive phosphorylation (“hyperphosphorylation”), which can be also reversed by dissolving these condensates. Such RNP condensates may promote RNA‐RNA interactions by increasing cognate RNA concentration bound by the RNA chaperone NSP2, thus being conducive to the assembly of eleven distinct transcripts required for packaging of a multi‐segmented viral genome.

Current views of viroplasm formation are dominated by the idea of multiple viral proteins being recruited into these inclusions in a specific order (Eichwald *et al*, 2004; Arnoldi *et al*, 2007; Criglar *et al*, 2014, 2018; Buttafuoco *et al*, 2020) resulting in a particular organisation (Garcés Suárez *et al*, 2019). Here, we propose a unifying model for viroplasm assembly (Fig 8) that takes into account multiple pieces of data gathered over decades and amounts to a step change in our understanding of the replication factories in these viruses. We propose that viroplasms represent condensates formed by NSP5/NSP2. Initially, NSP5/NSP2 condensates exhibit liquid‐like behaviour; however, their material properties (e.g., fluidity) dynamically change during infection, concomitant with changes in NSP5 phosphorylation (Poncet *et al*, 1997; Mohan *et al*, 2003; Sen *et al*, 2006; Sotelo *et al*, 2010; Criglar *et al*, 2014, 2018; Papa *et al*, 2020a). Other factors, e.g. RNA:protein ratio, likely contribute to condensate maturation (Garcia‐Jove Navarro *et al*, 2019; Choi *et al*, 2020).

Viral RNA‐binding proteins (e.g. viral RNA‐dependent RNA polymerase, RdRP and a capping enzyme VP3, Fig 8) bind NSP5 (Arnoldi *et al*, 2007; Viskovska *et al*, 2014) and partition into these condensates, yet not sufficient to form viroplasm‐like structures on their own, thus fulfilling the criteria of condensate clients (Ditlev *et al*, 2018). Remarkably, the proposed mechanism of viroplasm formation via LLPS is further corroborated by our recent observations that RV transcripts within these condensates can be targeted by catalytically active Cas6 endonucleases produced as NSP5 fusions in rotavirus‐infected cells (Papa *et al*, 2020b). Understanding selectivity of biomolecular condensates will offer exciting opportunities for improved targeting of viral RNAs prior to their assembly and packaging within these condensates. Furthermore, host components, including lipid bilayers (Feng *et al*, 2019), microtubules (Maucuer *et al*, 2018) and tubulin (King & Petry, 2020) can promote nucleation of biomolecular condensates and spatially regulate the kinetics of their formation in cells.

Thus, association of lipid droplets (Cheung *et al*, 2010; Crawford & Desselberger, 2016), tubulin (Carrẽo‐Torres *et al*, 2010; Criglar *et al*, 2014) and potentially other cellular components with viroplasms at later stages of infection does not contradict our model of LLPS‐driven formation of viroplasms. The observed differences between the early and late infection stage viroplasms (loss of fluidity/refractivity to aliphatic diol treatments) are also consistent with changes in material properties of condensates over time (Patel *et al*, 2015; Conicella *et al*, 2020; Ray *et al*, 2020). Given that multiple droplets are formed within the same RV‐infected cell (Fig 3A, 12 HPI), caution should be taken when interpreting their biochemical and physical properties, as they may significantly differ between individual condensates, whose molecular composition and organisation can dynamically change throughout the infection. Recent super‐resolution imaging studies of these organelles in RV‐infected cells proposed that distinct viral proteins are organised into multiple concentric layers (Garcés Suárez *et al*, 2019). The proposed model explains the relevance of these findings, as even very simple condensates show characteristics of multilayered behaviour (Choi *et al*, 2020). Distinct layers are likely to form via different molecular interaction networks that lead to different viscoelastic properties, such as those observed in nucleoli (Feric *et al*, 2016), P‐granules (Wei *et al*, 2017) and nuclear speckles (Fei *et al*, 2017).

### Implications for selective RNA recruitment and RNA–RNA interactions required for segmented genome assembly

Coacervation of viral RNA chaperone NSP2 (Borodavka *et al*, 2017, 2018; Bravo *et al*, 2018) associated with RV transcripts may accelerate formation of inter‐molecular interactions between the RNAs. Molecular crowding, depletion attraction and a highly polar environment of the interior of membraneless organelles(Nott *et al*, 2016) have all been shown to contribute to stabilisation of inter‐molecular RNA‐RNA contacts (Marenduzzo *et al*, 2006; Nott *et al*, 2016; Van Treeck & Parker, 2018), while promoting intra‐molecular duplex melting via interactions with multiple arginine side chains of NSP2 (Hu *et al*, 2012) that concentrate in the viroplasmic liquid phase. Recent evidence argues that inter‐molecular RNA ***–*** RNA interactions play a role in forming and determining the composition of distinct cytoplasmic, RNA‐rich ribonucleoprotein granules (Lin *et al*, 2015; Wheeler *et al*, 2016; Khong *et al*, 2017; Van Treeck & Parker, 2018; Tauber *et al*, 2020). In addition, the interior of such membraneless organelles could act as passive ATP‐independent helicases that can remodel nucleic acids and modulate RNA‐templated virus particle assembly within this environment (Nott *et al*, 2016).

Coalescence of multiple RNA‐binding proteins and non‐translating mRNAs lacking fixed stoichiometry occurs during cellular stress, giving rise to stress granules (Wheeler *et al*, 2016). Similarly, viroplasms accumulate non‐polyadenylated, untranslated viral transcripts and viral RNA‐binding proteins. While stress granules are highly enriched in poly(A)‐binding proteins associated with mRNAs, non‐polyadenylated rotaviral transcripts are likely to be bound by the viral RNA‐dependent RNA polymerase (RdRP), previously reported to have nM affinity for both NSP2 and NSP5 (Viskovska *et al*, 2014; Fig 8). Similar condensates enriched in RdRPs have been recently described in SARS‐CoV2‐infected cells (Savastano *et al*, 2020). Interestingly, most viral condensates reported to date have described phase separation of viral structural proteins, i.e. those present in mature virus particles. These include SARS‐CoV2 N‐protein (Iserman *et al*, 2020; Savastano *et al*, 2020), measles virus (Milles *et al*, 2018; Guseva *et al*, 2020) and rabies virus (Nikolic *et al*, 2017) N and P proteins, as well as N/P/L proteins of a vesicular stomatitis virus (Heinrich *et al*, 2018), and retroviral nucleocapsid proteins (Monette *et al*, 2020). In contrast, non‐structural proteins NSP5 and NSP2 are absent in rotavirions, yet both proteins play essential roles during RV replication and viral factory formation.

Recognition of early viroplasms as condensates nucleated by NSP5/NSP2 interactions opens several interesting avenues for investigating their molecular selectivity and dynamic interactions with other cellular organelles. The proposed model also poses many outstanding questions regarding how condensate maturation regulates viral replication, potentially in other segmented dsRNA viruses, whose replication factories also exhibit liquid‐like behaviour (Desmet *et al*, 2014; Campbell *et al*, 2020). LLPS‐driven mechanism of viroplasm formation offers a unified model that explains results from previous efforts (Papa *et al*, 2021), and demonstrates the feasibility of modulating LLPS for future antiviral strategies (Risso‐Ballester *et al*, 2021).

## Materials and Methods

### Cells and viruses

Rotavirus A strains (Bovine rotavirus strain RF and simian rotavirus SA11) were propagated as previously described (Arnold *et al*, 2012; Desselberger *et al*, 2013). All cell lines were maintained in Dulbecco’s Modified Eagle Medium (DMEM, 4.5 g/l glucose, supplemented with *L*‐glutamine and sodium pyruvate, Sigma) supplemented with 10% heat‐inactivated foetal calf serum (Sigma) at 37°C in the presence of 5% CO_2_. MA104) and its derivatives MA‐NSP2‐mCherry and MA‐NSP5‐EGFP stable cell lines were generated and maintained as described in refs. Eichwald *et al* (2004), Papa *et al* (2020a). Lentiviral vector pAIP‐NSP2‐SNAP was generated using a synthetic SNAP tag‐coding DNA (GenPart, Genscript) inserted into a double digested with *Mlu*I/*EcoR*I pAIP‐NSP2‐mCherry vector (Papa *et al*, 2020a). MA104‐NSP2‐SNAP cell line was then generated as previously described (Papa *et al*, 2020a). Briefly, 7 × 10^6^ HEK293T cells were seeded in 10‐cm^2^ tissue culture dishes 24 h before transfection. For each well, 2.4 μg of pMD2‐VSV‐G, 4 μg of pMDLg pRRE, 1.8 μg of pRSV‐Rev, and 1.5 μg of pAIP‐NSP2‐SNAP DNA constructs were co‐transfected using Lipofectamine 3000 (Sigma‐Aldrich) following the manufacturer’s instructions. After 48 h, the virus was harvested, filtered through a 0.45 mm polyvinylidene fluoride filter and immediately stored at −80°C. For lentiviral transduction, MA104 cells were transduced in six‐well plates with 1.2 ml of the lentivirus‐containing supernatant for 2 days. Cells were then selected by growing cells in DMEM supplemented with 10% FBS and puromycin (5 μg/ml) for 4 days. NSP5 immunostaining with polyclonal anti‐NSP5 sera (Papa *et al*, 2020a) (1:1,000 dilution) was carried out as described in (Papa *et al*, 2020a).

### Hyperosmotic adaptation and propylene glycol treatment of cells

#### Hyperosmotic adaptation

MA104 (ATCC CRL‐2378.1) cells were seeded into a T25 cm flask (10^6^ cells) in DMEM supplemented with 10% heat‐inactivated FCS (isosmotic medium). 14 h later, isosmotic medium was replaced with hyperosmotic PG‐containing medium (PGM: high glucose DMEM, 10% FCS, supplemented with 2% (v/v) PG). Cells were expanded under PG conditions for 72 h, after which they were harvested and seeded at 1:5 of their density to allow them to grow in the PGM medium. After 3–5 consecutive passages, PG‐adapted cells were seeded into 6‐well plates. PG‐adapted cells were washed twice with FCS‐free medium prior to infection with trypsin‐activated SA11 rotavirus.

#### PG treatment of RV‐infected cells

PG‐adapted MA104 cells (0.5 × 10^6^ cells) were seeded into 6‐well plates (Nunc 6‐well plates, Thermo Fisher) 48 h prior to infection with trypsin‐activated RVA strain SA11 (MOI of 10). Seeding was carried out in isosmotic medium, which was replaced with 2% (v/v) PG‐containing medium 24 h after seeding cells. Virus absorption was carried out at 37°C for 1 h, after which RV‐infected cells were washed twice with FCS‐free DMEM, followed by the application of 1 ml of fresh FCS‐free pre‐warmed DMEM medium. After 1 h of incubation at 37°C (1 HPI), isosmotic medium was replaced with either 2% PG‐containing medium, or 4.7% propylene glycol diluted in isosmotic FCS‐free DMEM. Cells were returned into the CO_2_ incubator for additional incubation periods as outlined in the Results section prior to harvesting the virus samples for subsequent quantitation. Virus‐infected cells were frozen and thawed twice, and RV‐containing cell lysates were clarified by centrifugation (8,000 g for 10 min) and frozen for further analysis.

#### Virus titration

RV‐containing clarified cell lysates were treated with 1 μg/ml of porcine trypsin (Sigma) at 37°C for 30 min. Viral titres were assayed using end‐point dilution method. Briefly, lysate samples were serially diluted with serum‐free DMEM culture medium supplemented with 0.5 μg/ml of porcine trypsin, and serial dilutions of inoculum were applied to confluent monolayers of MA104 cells seeded into wells of 24‐well plates. Wells were observed for signs of virus‐induced cytopathic effect (CPE) for 5 days after infection. Virus titres were calculated following the Reed and Muench method with calculated tissue culture infectious dose 50% (TCID50) converted to plaque‐forming units (PFU) using a conversion factor of 0.70 PFU/TCID50 (Distefano *et al*, 1995).

#### Virus replication kinetics

Confluent PG‐adapted MA104 cells grown on 9.6 cm^2^ dishes were infected with RV at a MOI of 10. After 60 min adsorption at 37°C, non‐internalised virus particles were removed with a brief rinse with 2 mM EGTA in PBS. RV‐infected cells were incubated and periodically harvested at 2, 4, 6, 8, 10 and 12 HPI. Virus‐containing cell lysates were prepared and harvested, as described above and stored at −20°C.

### Western blot analysis and NSP5 quantification

Protein samples and cell pellets were solubilised in Laemmli buffer, heat‐denatured at 98°C, and samples were resolved on 15% Tris‐glycine gels prior to transferring onto nitrocellulose membranes (Millipore, Bedford, MA). Membranes were blocked with PBS + 0.1% Tween‐20 (TBS) supplemented with 5% (v/v) skimmed milk (1 h), followed by 1 h incubation with guinea pig NSP5‐specific antibodies (Papa *et al*, 2020a) diluted 1:2,500 in PBS containing 5% milk. Membranes were then washed with TBS (three times, 5 min each) and incubated with anti‐guinea pig IgG (H + L) cross‐adsorbed secondary antibody (1:10,000; Invitrogen, #SA5‐10100) and anti‐actin hFAB rhodamine antibody (1:2,500; Bio‐Rad, #12004164) for 1 h prior to additional 3 washes with TBS. Fluorescent signals were detected using the Bio‐Rad ChemiDoc MP Imaging System (DyLight800/Rhodamine filters chosen). Images were further analysed using Image J software and quantified in Image Lab 6.1 (Bio‐Rad). Cytoplasmic NSP5 concentration was estimated from the quantitative Western blotting, by comparing integrated signal densities for NSP5 to those of known NSP5 standards (low loading concentration of 25–100 ng/band to ensure a linear response), estimated through the linear regression analysis of the intensities measured for known NSP5 standards. Estimated NSP5 concentrations (ng/band) were then converted into molar concentrations assuming the protein concentration of 21.5 kDa and the loading volume of 10 μl of lysate per lane. Given that 10^6^ infected cells were lysed in 100 μl of Laemmli buffer, and assuming the cytoplasmic volume of an epithelial cell (*Cercopithecus sp*.) of ˜ 2 pL (Fujioka *et al*, 2006).

### Transmission electron microscopy (TEM)

MA‐NSP5‐EGFP cells were seeded at 1 × 10^6^ cells in 6 cm^2^ wells and infected at MOI of 10. 12 hpi, cells were fixed with 0.5% glutaraldehyde in 200 mM sodium cacodylate buffer, pH 7.4, first on ice for 5 min, then at room temperature for 25 min, and washed 3 times with 200 mM sodium cacodylate buffer. Samples then were postfixed with 1% osmium tetroxide and 1.5% potassium ferrocyanide for 1 h at room temperature, washed with distilled water and stained in 0.5% magnesium‐uranyl acetate in water at 4°C overnight. Cells were washed with distilled water and dehydrated in a graded ethanol series starting at 70%, followed by two changes into absolute alcohol, and embedded in Epon resin. Ultrathin sections (70 nm) were cut parallel to the surface of the dish using a Leica ultramicrotome. The sections were collected onto 50 mesh formvar grids and stained with Reynold’s lead citrate for 30 s, washed with water and air‐dried. Samples were viewed with a FEI Tecnai G^2^ electron microscope with a Soft Imaging System Megaview III CCD camera. Images were collected at 1,376 × 1,032 × 16 pixels using AnalySIS version Docu software (Olympus Soft Imaging Solutions).

### Image data acquisition

Confocal imaging was conducted on a Zeiss Cell Observer SD inverted confocal microscope with a Yokogawa CSU‐X1 spinning disc unit from Zeiss (Jena, Germany), equipped with a 1.40 NA 63× Plan apochromat oil immersion objective (Zeiss). Cell imaging was carried out at 37°C. Fluorescence Recovery After Photobleaching (FRAP) experiments were carried out with a 488 nm laser at 100% intensity (148.3 μW) with 3,000 ms exposure time, and the recovery was observed for 60 frames every 30 s. EGFP was imaged using a 488 nm laser at 20% intensity (36.9 μW) and 200 ms exposure time, and mCherry was imaged with a 561 nm laser at 20% intensity (29.2 μW) and 200 ms exposure time. Images recorded as z‐stacks consisted of either 10 or 50 frames, with a 0.5 µm distance between them, depending on the sample. In the excitation path a quad‐edge dichroic beam splitter (FF410/504/582/669‐Di01‐25 × 36, Semrock) was used. For two colour detection of EGFP and mCherry, a dichroic mirror (660 nm, Semrock) and band‐pass filters 525/50 and 690/60 (both Semrock) were used in the detection path. Separate images for each fluorescence channel were acquired using two separate electron multiplier charge‐coupled devices (EMCCD) cameras (Photometrics Evolve^TM^). Image acquisition was controlled using the Zeiss Zen (blue edition) 2011 Software (Zeiss). Widefield imaging for smFISH was carried out using the Eclipse Ti‐E inverted microscope from Nikon (Tokyo, Japan). Images were acquired with a 0.7 NA 60 × S Plan Fluor ELWD oil immersion objective from Nikon. Measurements were performed at room temperature. A pE‐4000 illumination system (CoolLED) was used as light source. DAPI was imaged using a 385 nm LED at 33% intensity and 55 ms exposure time. EGFP and ATTO 488 were imaged using a 470 nm LED at 41% intensity with 300 ms exposure time and 7% intensity with 55 ms exposure time respectively. mCherry was imaged with a 550 nm LED at 36% intensity and 300 ms exposure time. The light path was regulated with a Dapi/FITC/Cy3/Cy5 Quad HC Filter Set (Semrock). The images were acquired using a scientific complementary metal oxide semiconductor (sCMOS) camera (Andor Technology). Image acquisition was controlled using the NIS‐Elements AR V.4.50 Software (Nikon). Imaging of the *in vitro* condensates was carried out using an ONI Nanoimager S microscope equipped with an Olympus 100× super apochromatic oil immersion objective (NA1.4). ONI laser illumination system was used for dye excitation at 488 nm (Atto488) and 641 nm (Atto 647N) with laser intensities set to 2% (488 nm) and 7% (641 nm). Fluorescent signals were recorded with a sCMOS camera with a pixel size of 0.117 µm. Images were acquired over a field of view of the camera chip resulting in a total imaging region of 50 µm × 80 µm. Exposure times were adjusted accordingly to the signal intensity to avoid pixel saturation. Typical exposure times were 33 ms for all channels. Images were recorded consecutively for each channel, from the lowest to the highest energy excitation wavelength.

### Image data processing

The recorded images were processed in ImageJ (v.1.52p; Schindelin *et al*, 2012). Data of the FRAP experiments were also analysed with ImageJ. Distinct visible granules were selected manually as ROIs before bleaching. The recovery curve over a time span of 13 min (corresponding to 60 frames) was calculated for each ROI. The displayed values are median intensities of five ROIs. Other parameters, including fusion events, velocity or sphericity, were analysed with Imaris (v 8.2.0, Bitplane, AG Zurich, Switzerland). Viroplasms were marked as ROIs based on their size and high fluorescence intensity. The centre of image mass R of the detected fluorescence volume in each ROI was calculated with the voxel intensity m_i_, the centre of a voxel r_i_ and the sum of voxel intensities, M.

$$R=\frac{1}{M}\sum_{i\in IsoSurface}m_{i}r_{i}$$

The coordinates of the centre of image mass of the ROIs were tracked for the duration of the experiment. These values were used to calculate velocity as change of the centre of image mass coordinates between two frames divided by the frame time (4.9 min). 65 ROIs were observed over a time span of 5.2 h.

Sphericity Ψ was calculated as the ratio of the surface area of a sphere with the same volume, as a given particle V_p_, to the surface area of the particle A_p_.

$$\Psi =\frac{\pi^{\frac{1}{3}}{\left(6V_{p}\right)}^{\frac{2}{3}}}{A_{P}}$$

Mean sphericity values were calculated for 65 ROIs monitored for EGFP‐marked granules observed for over 6 h. Fusion events were counted when two separate ROIs overlap and their volumes were treated as a single volume. 1,037 ROIs were detected over 8 h. If not stated otherwise, data points shown in figures represent mean values averaged over all measured ROIs. Measurements were all performed in biological triplicates. Further data plotting was carried out with OriginPro (Version 8.0891, OriginLab Corporation, Northampton, MA, USA), or GraphPad Prism (ver.9 for MacOS). Where appropriate, schematics of figures were prepared using BioRender.com.

### NSP5 and NSP2 expression and purification

Recombinant NSP2 (strain RF) was expressed and purified, as previously described (Borodavka *et al*, 2017). NTA‐affinity purified NSP2 fractions were further purified over a HiTrap SP cation‐exchange column. The concentrated peak fractions were resolved on a Superdex 200 10 × 300 GL column and pre‐equilibrated with RNAse‐free SEC buffer (25 mM HEPES‐Na, pH 7.5, 150 mM NaCl) to ensure high purity and homogeneity of the preparation. While a functional form of the RNA chaperone NSP2 can be produced and purified under native conditions (Jayaram *et al*, 2002; Borodavka *et al*, 2017), and its C‐terminally His‐tagged version supports viral replication (preprint: Bravo *et al*, 2020), previous attempts to natively purify a full‐length untagged NSP5 were not successful (Jiang *et al*, 2006; Martin *et al*, 2011). We therefore expressed and purified NSP5 under denaturing conditions, followed by its refolding. Full‐length recombinant NSP5 (strain RF) was expressed and isolated from bacterial pellets as inclusion bodies as previously described (Borodavka *et al*, 2017). Washed inclusion bodies were solubilised in 6 M guanidinium hydrochloride and the protein‐containing fraction was then subjected to a refolding protocol following step‐wise dialysis (Martin *et al*, 2011). After refolding, NSP5‐containing fractions were further purified over an ImpRes Q column (GE). The concentrated peak fractions were further resolved on a Superdex 200 10 × 300 column pre‐equilibrated with SEC buffer (25 mM HEPES‐Na, pH 7.5, 150 mM NaCl) to ensure homogeneity of the preparation. Quasi‐elastic scattering analysis of a monodisperse NSP5 sample revealed a hydrodynamic radius ˜6.8 nm, consistent with the previously proposed decameric organisation(Martin *et al*, 2011). ΔC‐NSP5‐expressing plasmid was generated by removing the C‐terminal residues 189–198 using the Q5 site‐directed mutagenesis kit and NEBaseChanger (NEB), and the original pET28‐NSP5 vector (Borodavka *et al*, 2017) as a template. Further expression and purification of the ΔC‐NSP5 mutant were carried out under the conditions identical to those used for full‐length NSP5. We noted that the yields of ΔC‐NSP5 were consistently higher compared to those seen for NSP5.

### DeePhase predictions

The propensity of the protein sequences to form condensates was estimated using the DeePhase model. Briefly, individual predictions relied on featuring the protein sequences by estimating a number of explicit sequence‐specific parameters (sequence length, hydrophobicity, Shannon entropy, the fraction of polar, aromatic and positively charged residues and the fraction of sequence estimated to be part of the low complexity region and intrinsically disordered region) as well as implicit word2vec algorithm‐based embeddings. The used model had been trained on previously constructed datasets including sequences with varying propensity to undergo LLPS as has been described in (Saar *et al*, 2021). In order to evaluate how the LLPS‐propensity of each protein sequence varied along its length, the full sequences were divided into 20–30 amino acid long fragments and the propensity of each fragment to undergo LLPS was evaluated. For the final result, individual predictions from 10 consecutive fragments were averaged.

### Circular dichroism spectroscopy and dynamic light scattering

Samples were prepared by dialysing NSP5 against 10 mM phosphate buffer pH 7.4, 150 mM sodium fluoride. Sodium fluoride was chosen to minimise strong absorbance of chloride ions at lower wavelengths. Spectra were acquired in a 1 mm path length quartz cuvette (Hellma) using a Chirascan plus spectrometer (Applied Photophysics) with a 1 nm bandwidth and a step size of 1 nm. An average of 3 scans (190–280 nm) were used for the final spectra, measured at 20°C and 90°C. Data were fitted to determine the secondary structure content using BeStSel (Micsonai *et al*, 2018).

NSP5 samples (1 mg ml^−1^) were injected on a TSKgel G6000PWxl SEC column (Tosoh) pre‐equilibrated with the SEC buffer (see above) at 21°C and a flow rate set to 0.4 ml min^−1^. Dynamic (Quasi‐Elastic) light scattering (QELS) measurements were carried out using an AKTA pure system (GE Healthcare) connected to a DAWN HELEOS and Optilab TrEX for QELS (Wyatt). On‐line QELS was carried out using WyattQELS DLS Module to measure the translational diffusion and corresponding hydrodynamic radius of the eluting fraction. Autocorrelation functions (ACFs) were fitted to a single exponential to determine diffusion coefficients and corresponding hydrodynamic radii (Rh) of the oligomeric NSP5 species using ASTRA software (Wyatt).

### High‐throughput generation of NSP5/NSP2 Phase Diagrams via PhaseScan

#### Device fabrication

Polydimethylsiloxane (PDMS, Corning) devices for droplet generation and multilayer well‐devices for droplet collection and imaging were produced on SU‐8 (Microchem) moulds fabricated via photolithographic processes as described previously (McDonald *et al*, 2000; Mazutis *et al*, 2013; Arter *et al*, 2020b).

#### Phase diagram generation

Phase diagrams were produced using droplet microfluidics in a similar manner to that described previously (Arter *et al*, 2020a). Syringe pumps (neMESYS modules, Cetoni) were used to control flows of protein solutions, consisting of 22 μM NSP5 supplemented with 6.4 μM Alexa 647 dye (carboxylic acid, Thermo Fisher) or 8 μM His‐tagged NSP2 labelled with 8 μM Atto488‐nitrilotriacetic acid (NTA, Sigma), and buffer (0.5 × phosphate saline buffer, PBS, pH 7.4). Appropriate quantities of 1,6‐hexanediol were pre‐mixed into all solutions before droplet generation. The aqueous flow rates were configured to vary automatically according to pre‐set gradients, with constant total flow rate of 60 μl/h, to scan phase space between nominal concentrations of 0.9–7.3 μM and 0.30–6.5 μM for NSP5 and NSP2, respectively FC‐40 oil (containing 1.5% (*v*/*v*) fluorosurfactant, RAN biotechnologies) was introduced to the device at a constant flow rate of 50 μl/h for microdroplet generation.

#### Imaging

Directly after generation, microdroplets were transferred into a droplet‐trapping device (Labanieh *et al*, 2015) to ensure droplets were maintained in a well‐spaced, stationary configuration for imaging. Microscopy data was acquired with a AxioObserver D1 microscope (Zeiss) equipped with a 5× air objective and a high‐sensitivity camera (Evolve 512, Photometrics). Appropriate filter sets were used for EFGP (49002, Chroma Technology) and Alexa Fluor 647 detection (49009, Chroma Technology). Representative data are presented in Fig EV5.

#### Droplet detection and data analysis

Acquired images were analysed using a custom‐written Python script. Droplets were fitted as circles in the images. Non‐circular droplets or erroneous detections were filtered and removed. From the fitted circular areas, the total intensity was calculated and normalised to obtain the intensity per unit volume (calculated using the fitted diameter) and converted to concentrations by comparison to calibration images acquired with known concentrations of NSP2/Atto488 and NSP5/Alexa 647 mixtures. Droplets were classified as phase‐separated or homogeneous according to the presence or absence of at least two connected pixels > 5 standard deviations from the mean pixel intensity. Representative classification output is presented Fig EV5. Droplet classification, NSP2 and NSP5 concentration were then combined on a per‐droplet basis to produce phase diagrams. Two‐dimensional probability maps were constructed by division of the phase space (NSP2 vs. NSP5 concentration) into regular squares. The proportion of homogeneous or phase‐separated droplets present in each region of phase space was calculated, before being passed through the error function (erf) to classify the phase separation propensity of each region as represented by the colourmap.

### Affinity measurements by fluorescence anisotropy

Fluorescence anisotropy measurements with Alexa Fluor 488 dye‐labelled 20‐mer RNA, as described previously (Bravo *et al*, 2018), were performed at 25°C using a POLARstar Omega plate reader (BMG Labtech) in Greiner 384 well black polypropylene plates. Serial 2‐fold dilutions of NSP2 were titrated into 5 nM RNA in 50 mM Tris–HCl pH 7.5, 50 mM NaCl, 1 mM EDTA, 0.05% Tween‐20 in a total volume of 50 µl and equilibrated at room temperature for 15 min prior to measurements were taken. Where required, buffers were supplemented with 4.5% v/v 1,2‐propanediol. Raw Anisotropy (r) values were calculated as follows:

$$r=\frac{\left(I\|‐I⊥\right)}{\left(I\|+2I⊥\right)}$$

where I‖ and I⊥ are the parallel and perpendicular emission signals, respectively. Normalised anisotropy values were plotted as a function of protein concentration and fitted to a Hill equation using OriginPro 9.0.

### Single‐molecule Fluorescence in situ Hybridisation (smFISH) and TagPAINT

Rotavirus‐infected and mock‐infected MA104 cell controls, where appropriate, were fixed with 4% (v/v) methanol‐free paraformaldehyde in nuclease‐free phosphate saline buffer (PBS, Sigma) for 10 min at room temperature. Samples were then washed twice with PBS, and fixed cells were permeabilised with 70% (v/v) ethanol (200 proof) in RNAse‐free water and stored in ethanol at +4°C for at least 12 h prior to hybridisation, and no longer than 24 h. Permeabilised cells were then re‐hydrated for 5 min in a pre‐hybridisation buffer (300 mM NaCl, 30 mM trisodium citrate, pH 7.0 in nuclease‐free water, 10% v/v Hi‐Di formamide (Thermo Scientific), supplemented with 2 mM vanadyl ribonucleoside complex). Re‐hydrated samples were hybridised with an equimolar mixture of DNA probes specific to the RNA targets (RVA strain RF sequences), 62.5 nM final concentration, see Table EV1, in a total volume of 200 µl of the hybridisation buffer (Stellaris RNA FISH hybridisation buffer, Biosearch Technologies, supplemented with 10% v/v Hi‐Di formamide). After 4 h of incubation at 37°C in a humidified chamber, samples were briefly rinsed with the wash buffer (300 mM NaCl, 30 mM trisodium citrate, pH 7.0, 10% v/v formamide in nuclease‐free water, after which a fresh aliquot of 0.3 ml of the wash buffer was applied to each well and incubated twice at 37°C for 30 min. After washes, nuclei were briefly stained with 300 nM 4’,6‐diamidino‐2‐phenylindole (DAPI) solution in 300 mM NaCl, 30 mM trisodium citrate, pH 7.0) and the samples were finally rinsed with and stored in the same buffer without DAPI prior to the addition of the photostabilising imaging buffer (PBS containing an oxygen scavenging system of 2.5 mM protocatechuic acid, 10 nM protocatechuate‐3,4‐dioxygenase supplemented with 1 mM (±)‐6‐hydroxy‐2,5,7,8‐tetramethylchromane‐2‐carboxylic acid (Trolox). TagPAINT imaging (Nieves *et al*, 2019) was carried out for SNAP‐tagged NSP2‐expressing cells infected with RVs. After fixation, cells were permeabilised with PBS supplemented with 0.2% Triton‐X100 for 3 min and then subsequently incubated with 50 mg ml^−1^ BSA in PBS for 10 min. 5 μM benzylguanine (BG)‐conjugated DNA (Biomers.com) dissolved in PBS supplemented with 0.2% Tween‐20 (PBST) was incubated with fixed cell samples for 15 min. The samples were then washed with 0.4 ml of PBST several times, to remove any non‐specifically adsorbed ligand. Finally, the samples were incubated with gold nanoparticles in PBST for 10 min before mounting for DNA‐PAINT imaging.

### DNA‐PAINT imaging

#### Microscope configuration

DNA‐PAINT imaging was carried out on an inverted Nikon Eclipse Ti microscope (Nikon Instruments) equipped with the Perfect Focus System using objective‐type total internal reflection fluorescence (TIRF) configuration (oil immersion Apo SR TIRF, NA 1.49 100× objective). A 200 mW 561 nm laser beam (Coherent Sapphire) was passed through a clean‐up filter (ZET561/10, Chroma Technology) and coupled into the microscope objective using a beam splitter (ZT561rdc, Chroma Technology). Fluorescence light was spectrally filtered with an emission filter (ET575lp, Chroma Technology) and imaged with a sCMOS camera (Andor Zyla 4.2) without further magnification, resulting in an effective pixel size of 130 nm after 2 × 2 binning. Images were acquired using a region of interest of 512 × 512 pixels. The camera read‐out rate was set to 540 MHz, and images were acquired with an integration time of 200 ms.

### Sample preparation, imaging and data analysis

5’‐ATACATTGA‐Cy3B‐3’ (Metabion) was used as ssDNA “imager” for visualising Seg 3 RNA target. 20,000 frames were acquired for each target. Sequences of the oligonucleotide RNA FISH probes are described in ref. Strauss *et al* (2021). These were generated using the Stellaris RNA FISH probe designer (https://www.biosearchtech.com/stellaris‐designer), using each gene‐specific ORF sequence as inputs and level 2 masking. The resulting pools of probes were then further filtered to remove the sequences targeting the RNA transcripts sequences with higher propensity to form stable intra‐molecular base‐pairing. 5’‐TAATGAAGA‐Cy3B‐3’ (Metabion) was used as ssDNA “imager” for a complementary benzylguanine (BG)‐conjugated oligonucleotide DNA (Biomers.com) for reacting with the SNAP‐tagged NSP2. Imager strands were diluted to 100 pM (Seg3 RNA), and 300 pM (SNAP‐tagged NSP2), respectively. Drift correction was performed with a redundant cross‐correlation and gold particles used as fiducial markers. Fluorescence data were subjected to super‐resolution reconstruction using Picasso software package (Jungmann *et al*, 2014; Schnitzbauer *et al*, 2017).

### Statistical analysis

Statistical analyses of all repeated measurements where appropriate, and as indicated in figure legends, were carried out in GraphPad Prism version 9.1.1 (223) for MacOS, GraphPad Software, San Diego, California USA.

## Author contributions

AB, FG, JA, GP, XW, WEA, GK, HE, KLS, JPKB, SS designed and/or carried out experiments and analysed data. KLS, WEA, NAE, RQ, GK, TPJK, RJ, ORB contributed analytical tools. AB managed the project. All authors contributed ideas, discussed the results and were involved in writing of the manuscript.

## Conflict of interest

Parts of this work have been the subject of a patent application filed by Cambridge Enterprise Limited, a fully owned subsidiary of the University of Cambridge. T.P.J.K. is a founder, and W.E.A. is an employee, and K.L.S., G.K. and A.B. are consultants for Transition Bio Inc.

## Supporting information



Expanded View Figures PDF

Movie EV1

Movie EV2

Movie EV3

Table EV1

Source Data for Expanded View

Source Data for Figure 2

Source Data for Figure 4

Source Data for Figure 5

Source Data for Figure 6

Source Data for Figure 7

## Data Availability

Primary image datasets and stacks of 3D DNA‐PAINT images are available at: http://doi.org/10.5281/zenodo.5009222. DeePhase tool is available at: https://deephase.ch.cam.ac.uk.

## References

[embj2021107711-bib-0001] Alberti S (2017) Phase separation in biology. Curr Biol 27: R1097–R1102 29065286 10.1016/j.cub.2017.08.069

[embj2021107711-bib-0002] Alberti S , Gladfelter A , Mittag T (2019) Considerations and challenges in studying liquid‐liquid phase separation and biomolecular condensates. Cell 176: 419–434 30682370 10.1016/j.cell.2018.12.035 PMC6445271

[embj2021107711-bib-0003] Albro MB , Petersen LE , Li R , Hung CT , Ateshian GA (2009) Influence of the partitioning of osmolytes by the cytoplasm on the passive response of cells to osmotic loading. Biophys J 97: 2886–2893 19948117 10.1016/j.bpj.2009.09.011 PMC2784563

[embj2021107711-bib-0004] Alenquer M , Vale‐Costa S , Etibor TA , Ferreira F , Sousa AL , Amorim MJ (2019) Influenza A virus ribonucleoproteins form liquid organelles at endoplasmic reticulum exit sites. Nat Commun 10: 1–19 30967547 10.1038/s41467-019-09549-4 PMC6456594

[embj2021107711-bib-0005] Altenburg BC , Graham DY , Estes MK (1980) Ultrastructural Study of Rotavirus Replication in Cultured Cells. J Gen Virol 46: 75–85 6243348 10.1099/0022-1317-46-1-75

[embj2021107711-bib-0006] Aponte C , Poncet D , Cohen J (1996) Recovery and characterization of a replicase complex in rotavirus‐infected cells by using a monoclonal antibody against NSP2. J Virol 70: 985–991 8551639 10.1128/jvi.70.2.985-991.1996 PMC189903

[embj2021107711-bib-0007] Arnold M , Patton JT , McDonald SM (2009) Culturing, storage, and quantification of Rotaviruses. Curr Protoc Microbiol, 15. 10.1002/9780471729259.mc15c03s15 PMC3403738 19885940

[embj2021107711-bib-0008] Arnoldi F , Campagna M , Eichwald C , Desselberger U , Burrone OR (2007) Interaction of rotavirus polymerase VP1 with nonstructural protein NSP5 is stronger than that with NSP2. J Virol 81: 2128–2137 17182692 10.1128/JVI.01494-06 PMC1865955

[embj2021107711-bib-0009] Arter WE , Qi R , Krainer G , Welsh TJ , Xu Y (2020a) Rapid generation of protein condensate phase diagrams using combinatorial droplet microfluidics. bioRxiv. 10.1101/2020.06.04.132308

[embj2021107711-bib-0010] Arter WE , Yusim Y , Peter Q , Taylor CG , Klenerman D , Keyser UF , Knowles TPJ (2020b) Digital sensing and molecular computation by an enzyme‐free DNA circuit. ACS Nano 14: 5763–5771 32293175 10.1021/acsnano.0c00628

[embj2021107711-bib-0011] Aumiller WM , Keating CD (2016) Phosphorylation‐mediated RNA/peptide complex coacervation as a model for intracellular liquid organelles. Nat Chem 8: 129–137 26791895 10.1038/nchem.2414

[embj2021107711-bib-0012] Bah A , Vernon RM , Siddiqui Z , Krzeminski M , Muhandiram R , Zhao C , Sonenberg N , Kay LE , Forman‐Kay JD (2015) Folding of an intrinsically disordered protein by phosphorylation as a regulatory switch. Nature 519: 106–109 25533957 10.1038/nature13999

[embj2021107711-bib-0013] Banani SF , Lee HO , Hyman AA , Rosen MK (2017) Biomolecular condensates: organizers of cellular biochemistry. Nat Rev Mol Cell Biol 18: 285–298 28225081 10.1038/nrm.2017.7 PMC7434221

[embj2021107711-bib-0014] Bergeron‐Sandoval L‐P , Michnick SW (2018) Mechanics, Structure and Function of Biopolymer Condensates. J Mol Biol 430: 4754–4761 29913159 10.1016/j.jmb.2018.06.023

[embj2021107711-bib-0015] Bergeron‐Sandoval LP , Safaee N , Michnick SW (2016) Mechanisms and consequences of macromolecular phase separation. Cell 165: 1067–1079 27203111 10.1016/j.cell.2016.05.026

[embj2021107711-bib-0016] Berois M , Sapin C , Erk I , Poncet D , Cohen J (2003) Rotavirus nonstructural protein NSP5 interacts with major core protein VP2. J Virol 77: 1757–1763 12525609 10.1128/JVI.77.3.1757-1763.2003 PMC140918

[embj2021107711-bib-0017] Boeynaems S , Holehouse AS , Weinhardt V , Kovacs D , Van Lindt J , Larabell C , Van Den Bosch L , Das R , Tompa PS , Pappu RV *et al* (2019) Spontaneous driving forces give rise to protein−RNA condensates with coexisting phases and complex material properties. Proc Natl Acad Sci USA 116: 7889–7898 30926670 10.1073/pnas.1821038116 PMC6475405

[embj2021107711-bib-0018] Borodavka A , Desselberger U , Patton JT (2018) Genome packaging in multi‐segmented dsRNA viruses: distinct mechanisms with similar outcomes. Curr Opin Virol 33: 106–112 30145433 10.1016/j.coviro.2018.08.001 PMC6289821

[embj2021107711-bib-0019] Borodavka A , Dykeman EC , Schrimpf W , Lamb DC (2017) Protein‐mediated RNA folding governs sequence‐specific interactions between rotavirus genome segments. Elife 6: 1–22 10.7554/eLife.27453 PMC5621836 28922109

[embj2021107711-bib-0020] Brangwynne CP (2011) Soft active aggregates: Mechanics, dynamics and self‐assembly of liquid‐like intracellular protein bodies. Soft Matter 7: 3052–3059

[embj2021107711-bib-0021] Brangwynne CP , Tompa P , Pappu RV (2015) Polymer physics of intracellular phase transitions. Nat Phys 11: 899–904

[embj2021107711-bib-0022] Bravo JPK , Bartnik K , Venditti L , Acker J , Gail EH , Colyer A , Davidovich C , Lamb DC , Tuma R , Calabrese AN *et al* (2021) Structural basis of rotavirus RNA chaperone displacement and RNA annealing. PNAS. 10.1073/pnas.2100198118 [PREPRINT] PMC8521686 34615715

[embj2021107711-bib-0023] Bravo JPK , Borodavka A , Barth A , Calabrese AN , Mojzes P , Cockburn J , Lamb DC , Tuma R (2018) Stability of local secondary structure determines selectivity of viral RNA chaperones. Nucleic Acids Res 46: 7924–7937 29796667 10.1093/nar/gky394 PMC6125681

[embj2021107711-bib-0024] Buttafuoco A , Michaelsen K , Tobler K , Ackermann M , Fraefel C , Eichwald C (2020) Conserved rotavirus NSP5 and VP2 domains interact and affect viroplasm. J Virol 94: 1–21 10.1128/JVI.01965-19 PMC7081909 31915278

[embj2021107711-bib-0025] Campagna M , Budini M , Arnoldi F , Desselberger U , Allende JE , Burrone OR (2007) Impaired hyperphosphorylation of rotavirus NSP5 in cells depleted of casein kinase 1α is associated with the formation of viroplasms with altered morphology and a moderate decrease in virus replication. J Gen Virol 88: 2800–2810 17872534 10.1099/vir.0.82922-0

[embj2021107711-bib-0026] Campbell EA , Reddy VRAP , Gray AG , Wells J , Simpson J , Skinner MA , Hawes PC , Broadbent AJ (2020) Discrete virus factories form in the cytoplasm of cells coinfected with two replication‐competent tagged reporter birnaviruses that subsequently coalesce over time. J Virol 94: 1–16 10.1128/JVI.02107-19 PMC7307154 32321810

[embj2021107711-bib-0027] Carrẽo‐Torres JJ , Gutiérrez M , Arias CF , Lápez S , Isa P , Carreño‐torres JJ , Gutiérrez M , Arias CF , López S , Isa P (2010) Characterization of viroplasm formation during the early stages of rotavirus infection. Virol J 7: 350 21114853 10.1186/1743-422X-7-350 PMC3009706

[embj2021107711-bib-0028] Cheung W , Gill M , Esposito A , Kaminski CF , Courousse N , Trugnan G , Keshavan N , Desselberger U , Chwetzoff S , Lever A (2010) Rotaviruses associate with cellular lipid droplet components to replicate in viroplasms, and compounds disrupting or blocking lipid droplets inhibit viroplasm formation and viral replication. J Virol 84: 6782–6798 20335253 10.1128/JVI.01757-09 PMC2903253

[embj2021107711-bib-0029] Choi J‐M , Holehouse AS , Pappu RV (2020) Physical principles underlying the complex biology of intracellular phase transitions. Annu Rev Biophys 49: 107–133 32004090 10.1146/annurev-biophys-121219-081629 PMC10715172

[embj2021107711-bib-0030] Conicella AE , Dignon GL , Zerze GH , Schmidt HB , D’Ordine AM , Kim YC , Rohatgi R , Ayala YM , Mittal J , Fawzi NL (2020) TDP‐43 α ‐helical structure tunes liquid – liquid phase separation and function. Proc Natl Acad Sci 117: 5883–5894 32132204 10.1073/pnas.1912055117 PMC7084079

[embj2021107711-bib-0031] Contin R , Arnoldi F , Campagna M , Burrone OR (2010) Rotavirus NSP5 orchestrates recruitment of viroplasmic proteins. J Gen Virol 91: 1782–1793 20200190 10.1099/vir.0.019133-0

[embj2021107711-bib-0032] Crawford SE , Desselberger U (2016) Lipid droplets form complexes with viroplasms and are crucial for rotavirus replication. Curr Opin Virol 19: 11–15 27341619 10.1016/j.coviro.2016.05.008 PMC5125616

[embj2021107711-bib-0033] Criglar JM , Anish R , Hu L , Crawford SE , Sankaran B , Prasad BVV , Estes MK (2018) Phosphorylation cascade regulates the formation and maturation of rotaviral replication factories. Proc Natl Acad Sci 115: E12015–E12023 30509975 10.1073/pnas.1717944115 PMC6304940

[embj2021107711-bib-0034] Criglar JM , Hu L , Crawford SE , Hyser JM , Broughman JR , Prasad BVV , Estes MK (2014) A novel form of rotavirus NSP2 and phosphorylation‐dependent NSP2‐NSP5 interactions are associated with viroplasm assembly. J Virol 88: 786–798 24198401 10.1128/JVI.03022-13 PMC3911676

[embj2021107711-bib-0035] Dai M , Jungmann R , Yin P (2016) Optical imaging of individual biomolecules in densely packed clusters. Nat Nano 11: 798–807 10.1038/nnano.2016.95 PMC5014615 27376244

[embj2021107711-bib-0036] Desmet EA , Anguish LJ , Parker JSL (2014) Virus‐mediated compartmentalization of the host translational machinery. MBio 5: 1–11 10.1128/mBio.01463-14 PMC4172071 25227463

[embj2021107711-bib-0037] Desselberger U , Richards J , Tchertanov L , Lepault J , Lever A , Burrone O , Cohen J (2013) Further characterisation of rotavirus cores: Ss(+)RNAs can be packaged in vitro but packaging lacks sequence specificity. Virus Research 178: 252–263 24091366 10.1016/j.virusres.2013.09.034 PMC3854842

[embj2021107711-bib-0038] Distefano DJ , Gould SL , Munshi S , Robinson DK (1995) Titration of human‐bovine rotavirus reassortants using a tetrazolium‐based colorimetric end‐point dilution assay. J Virol Methods 55: 199–208 8537458 10.1016/0166-0934(95)00057-2

[embj2021107711-bib-0039] Ditlev JA , Case LB , Rosen MK (2018) Who’s in and who’s out — Compositional control of biomolecular condensates. J Mol Biol 430: 4666–4684 30099028 10.1016/j.jmb.2018.08.003 PMC6204295

[embj2021107711-bib-0040] Eichwald C , Arnoldi F , Laimbacher AS , Schraner EM , Fraefel C , Wild P , Burrone OR , Ackermann M (2012) Rotavirus viroplasm fusion and perinuclear localization are dynamic processes requiring stabilized microtubules. PLoS One 7: 1–16 10.1371/journal.pone.0047947 PMC3479128 23110139

[embj2021107711-bib-0041] Eichwald C , De Lorenzo G , Schraner EM , Papa G , Bollati M , Swuec P , de Rosa M , Milani M , Mastrangelo E , Ackermann M *et al* (2018) Identification of a small molecule that compromises the structural integrity of viroplasms and rotavirus double‐layered particles. J Virol 92: e01943‐17 29142132 10.1128/JVI.01943-17 PMC5774888

[embj2021107711-bib-0042] Eichwald C , Rodriguez JF , Burrone OR (2004) Characterization of rotavirus NSP2/NSP5 interactions and the dynamics of viroplasm formation. J Gen Virol 85: 625–634 14993647 10.1099/vir.0.19611-0

[embj2021107711-bib-0043] Fabbretti E , Afrikanova I , Vascotto F , Burrone OR (1999) Two non‐structural rotavirus proteins, NSP2 and NSP5, form viroplasm‐like structures in vivo. J Gen Virol 80: 333–339 10073692 10.1099/0022-1317-80-2-333

[embj2021107711-bib-0044] Fei J , Jadaliha M , Harmon TS , Li ITS , Hua B , Hao Q , Holehouse AS , Reyer M , Sun Q , Freier SM *et al* (2017) Quantitative analysis of multilayer organization of proteins and RNA in nuclear speckles at super resolution. J Cell Sci 130: 4180–4192 29133588 10.1242/jcs.206854 PMC5769577

[embj2021107711-bib-0045] Feng Z , Chen X , Wu X , Zhang M (2019) Formation of biological condensates via phase separation: Characteristics, analytical methods, and physiological implications. J Biol Chem 294: 14823–14835 31444270 10.1074/jbc.REV119.007895 PMC6779427

[embj2021107711-bib-0046] Feric M , Vaidya N , Harmon TS , Mitrea DM , Zhu L , Richardson TM , Kriwacki RW , Pappu RV , Brangwynne CP (2016) Coexisting liquid phases underlie nucleolar subcompartments. Cell 165: 1686–1697 27212236 10.1016/j.cell.2016.04.047 PMC5127388

[embj2021107711-bib-0047] Fujioka A , Terai K , Itoh RE , Aoki K , Nakamura T , Kuroda S , Nishida E , Matsuda M (2006) Dynamics of the Ras/ERK MAPK cascade as monitored by fluorescent probes. J Biol Chem 281: 8917–8926 16418172 10.1074/jbc.M509344200

[embj2021107711-bib-0048] Garcés Suárez Y , Martínez JL , Torres Hernández D , Hernández HO , Pérez‐Delgado A , Méndez M , Wood CD , Rendon‐Mancha JM , Silva‐Ayala D , López S *et al* (2019) Nanoscale organization of rotavirus replication machineries. Elife 8: e42906 31343403 10.7554/eLife.42906 PMC6692110

[embj2021107711-bib-0049] Garcia‐Jove Navarro M , Kashida S , Chouaib R , Souquere S , Pierron G , Weil D , Gueroui Z (2019) RNA is a critical element for the sizing and the composition of phase‐separated RNA–protein condensates. Nature Communications, 10. 10.1038/s41467-019-11241-6 PMC6642089 31324804

[embj2021107711-bib-0050] Guseva S , Milles S , Jensen MR , Salvi N , Kleman JP , Maurin D , Ruigrok RWH , Blackledge M (2020) Measles virus nucleo‐ and phosphoproteins form liquid‐like phase‐separated compartments that promote nucleocapsid assembly. Sci Adv 6: 1–12 10.1126/sciadv.aaz7095 PMC7112944 32270045

[embj2021107711-bib-0051] Hastings RL , Boeynaems S (2021) Designer Condensates: A Toolkit for the Biomolecular Architect: FASE toolkit for synthetic condensates. J Mol Biol 433: 166837 33539874 10.1016/j.jmb.2021.166837

[embj2021107711-bib-0052] Heinrich BS , Maliga Z , Stein DA , Hyman AA , Whelan SPJ (2018) Phase transitions drive the formation of vesicular stomatitis virus replication compartments. MBio 9: 1–10 10.1128/mBio.02290-17 PMC6123442 30181255

[embj2021107711-bib-0053] Hu G , Katuwawala A , Wang K , Wu Z , Ghadermarzi S , Gao J , Kurgan L (2021) flDPnn: Accurate intrinsic disorder prediction with putative propensities of disorder functions. Nat Commun 12: 4438 34290238 10.1038/s41467-021-24773-7 PMC8295265

[embj2021107711-bib-0054] Hu L , Chow D‐C , Patton JT , Palzkill T , Estes MK , Prasad BVV (2012) Crystallographic analysis of rotavirus NSP2‐RNA complex reveals specific recognition of 5′ GG sequence for RTPase activity. J Virol 86: 10547–10557 22811529 10.1128/JVI.01201-12 PMC3457270

[embj2021107711-bib-0055] Iserman C , Roden CA , Boerneke MA , Sealfon RSG , McLaughlin GA , Jungreis I , Fritch EJ , Hou YJ , Ekena J , Weidmann CA *et al* (2020) Genomic RNA elements drive phase separation of the SARS‐CoV‐2 nucleocapsid. Mol Cell 80: 1078–1091.e6 33290746 10.1016/j.molcel.2020.11.041 PMC7691212

[embj2021107711-bib-0056] Jayaram H , Taraporewala Z , Patton JT , Prasad BVV (2002) Rotavirus protein involved in genome replication and packaging exhibits a HIT‐like fold. Nature 417: 311–315 12015608 10.1038/417311a

[embj2021107711-bib-0057] Jiang X , Jayaram H , Kumar M , Ludtke SJ , Estes MK , Prasad BVV (2006) Cryoelectron microscopy structures of rotavirus NSP2‐NSP5 and NSP2‐RNA complexes: implications for genome replication. J Virol 80: 10829–10835 16928740 10.1128/JVI.01347-06 PMC1641785

[embj2021107711-bib-0058] Jumper J , Evans R , Pritzel A , Green T , Figurnov M , Ronneberger O , Tunyasuvunakool K , Bates R , Žídek A , Potapenko A *et al* (2021) Highly accurate protein structure prediction with AlphaFold. Nature 596: 583–589 34265844 10.1038/s41586-021-03819-2 PMC8371605

[embj2021107711-bib-0059] Jungmann R , Avendano MS , Woehrstein JB , Dai M , Shih WM , Yin P (2014) Multiplexed 3D cellular super‐resolution imaging with DNA‐PAINT and exchange‐PAINT. Nat Meth 11: 313–318 10.1038/nmeth.2835 PMC4153392 24487583

[embj2021107711-bib-0060] Kaur T , Raju M , Alshareedah I , Davis RB , Potoyan DA , Banerjee PR (2021) Sequence‐encoded and composition‐dependent protein‐RNA interactions control multiphasic condensate morphologies. Nat Commun 12: 872 33558506 10.1038/s41467-021-21089-4 PMC7870978

[embj2021107711-bib-0061] Khong A , Matheny T , Jain S , Mitchell SF , Wheeler JR , Parker R (2017) The stress granule transcriptome reveals principles of mRNA accumulation in stress granules. Mol Cell 68: 808–820 29129640 10.1016/j.molcel.2017.10.015 PMC5728175

[embj2021107711-bib-0062] King MR , Petry S (2020) Phase separation of TPX2 enhances and spatially coordinates microtubule nucleation. Nat Commun 11: 1–13 31937751 10.1038/s41467-019-14087-0 PMC6959270

[embj2021107711-bib-0063] Knowles TPJ , Vendruscolo M , Dobson CM (2014) The amyloid state and its association with protein misfolding diseases. Nat Rev Mol Cell Biol 15: 384–396 24854788 10.1038/nrm3810

[embj2021107711-bib-0064] Kroschwald S , Maharana S , Simon A (2017) Hexanediol: a chemical probe to investigate the material properties of membrane‐less compartments. Matters 10.19185/matters.201702000010

[embj2021107711-bib-0065] Labanieh L , Nguyen TN , Zhao W , Kang DK (2015) Floating droplet array: An ultrahigh‐throughput device for droplet trapping, real‐time analysis and recovery. Micromachines 6: 1469–1482 27134760 10.3390/mi6101431 PMC4849166

[embj2021107711-bib-0066] Langdon EM , Gladfelter AS (2018) A new lens for RNA localization: liquid‐liquid phase separation. Annu Rev Microbiol 72: 255–271 30200855 10.1146/annurev-micro-090817-062814

[embj2021107711-bib-0067] Li P , Banjade S , Cheng H‐C , Kim S , Chen B , Guo L , Llaguno M , Hollingsworth JV , King DS , Banani SF *et al* (2012) Phase transitions in the assembly of multivalent signalling proteins. Nature 483: 336–340 22398450 10.1038/nature10879 PMC3343696

[embj2021107711-bib-0068] Lin YI , Mori E , Kato M , Xiang S , Wu L , Kwon I , McKnight SL (2016) Toxic PR poly‐dipeptides encoded by the C9orf72 repeat expansion target LC domain polymers. Cell 167: 789–802 27768897 10.1016/j.cell.2016.10.003 PMC5076566

[embj2021107711-bib-0069] Lin Y , Protter DSW , Rosen MK , Parker R (2015) Formation and maturation of phase‐separated liquid droplets by RNA‐binding proteins. Mol Cell 60: 208–219 26412307 10.1016/j.molcel.2015.08.018 PMC4609299

[embj2021107711-bib-0070] Lu X , McDonald SM , Tortorici MA , Tao YJ , Vasquez‐Del Carpio R , Nibert ML , Patton JT , Harrison S (2008) Mechanism for coordinated RNA packaging and genome replication by rotavirus polymerase VP1. Structure 16: 1678–1688 19000820 10.1016/j.str.2008.09.006 PMC2602806

[embj2021107711-bib-0071] Marenduzzo D , Finan K , Cook PR (2006) The depletion attraction: an underappreciated force driving cellular organization. J Cell Biol 175: 681–686 17145959 10.1083/jcb.200609066 PMC2064666

[embj2021107711-bib-0072] Martin D , Ouldali M , Ménétrey J , Poncet D (2011) Structural organisation of the rotavirus nonstructural protein NSP5. J Mol Biol 413: 209–221 21864538 10.1016/j.jmb.2011.08.008

[embj2021107711-bib-0073] Maucuer A , Desforges B , Joshi V , Boca M , Kretov DA , Hamon L , Bouhss A , Curmi PA , Pastré D (2018) Microtubules as platforms for probing liquid–liquid phase separation in cells – application to RNA‐binding proteins. J Cell Sci 131: jcs214692 29728455 10.1242/jcs.214692 PMC6031325

[embj2021107711-bib-0074] Mazutis L , Gilbert J , Ung WL , Weitz DA , Griffiths AD , Heyman JA (2013) Single‐cell analysis and sorting using droplet‐based microfluidics. Nat Protoc 8: 870–891 23558786 10.1038/nprot.2013.046 PMC4128248

[embj2021107711-bib-0075] McDonald JC , Duffy DC , Anderson JR , Chiu DT , Wu H , Schueller OJA , Whitesides GM (2000) Fabrication of microfluidic systems in Poly(dimethylsiloxane). Electrophoresis 21: 27–40 10634468 10.1002/(SICI)1522-2683(20000101)21:1<27::AID-ELPS27>3.0.CO;2-C

[embj2021107711-bib-0076] Micsonai A , Wien F , Bulyáki É , Kun J , Moussong É , Lee YH , Goto Y , Réfrégiers M , Kardos J (2018) BeStSel: a web server for accurate protein secondary structure prediction and fold recognition from the circular dichroism spectra. Nucleic Acids Res 46: W315–W322 29893907 10.1093/nar/gky497 PMC6031044

[embj2021107711-bib-0077] Milles S , Jensen MR , Lazert C , Guseva S , Ivashchenko S , Communie G , Maurin D , Gerlier D , Ruigrok RWH , Blackledge M (2018) An ultraweak interaction in the intrinsically disordered replication machinery is essential for measles virus function. Sci Adv 4: eaat7778 30140745 10.1126/sciadv.aat7778 PMC6105297

[embj2021107711-bib-0078] Mochida K , Gomyoda M (1987) Toxicity of ethylene glycol, diethylene glycol, and propylene glycol to human cells in culture. Bull Environ Contam Toxicol 38: 151–153 3814844 10.1007/BF01606573

[embj2021107711-bib-0079] Mohan KVK , Muller J , Som I , Atreya CD (2003) The N‐ and C‐terminal regions of rotavirus NSP5 are the critical determinants for the formation of viroplasm‐like structures independent of NSP2. J Virol 77: 12184–12192 14581555 10.1128/JVI.77.22.12184-12192.2003 PMC254265

[embj2021107711-bib-0080] Monette A , Niu M , Chen L , Rao S , Gorelick RJ , Mouland AJ (2020) Pan‐retroviral nucleocapsid‐mediated phase separation regulates genomic RNA positioning and trafficking. Cell Rep 31: 107520 32320662 10.1016/j.celrep.2020.03.084 PMC8965748

[embj2021107711-bib-0081] Nieves DJ , Hilzenrat G , Tran J , Yang Z , MacRae HH , Baker MAB , Gooding JJ , Gaus K (2019) TagPAINT: Covalent labelling of genetically encoded protein tags for DNA‐PAINT imaging. R Soc Open Sci 6: 191268 31903209 10.1098/rsos.191268 PMC6936279

[embj2021107711-bib-0082] Nikolic J , Le Bars R , Lama Z , Scrima N , Lagaudrière‐Gesbert C , Gaudin Y , Blondel D (2017) Negri bodies are viral factories with properties of liquid organelles. Nat Commun 8: 58 28680096 10.1038/s41467-017-00102-9 PMC5498545

[embj2021107711-bib-0083] Nott TJ , Craggs TD , Baldwin AJ (2016) Membraneless organelles can melt nucleic acid duplexes and act as biomolecular filters. Nat Chem 8: 569–575 27219701 10.1038/nchem.2519

[embj2021107711-bib-0084] Papa G , Borodavka A , Desselberger U (2021) Viroplasms: assembly and functions of rotavirus replication factories. Viruses 13: 1349 34372555 10.3390/v13071349 PMC8310052

[embj2021107711-bib-0085] Papa G , Venditti L , Arnoldi F , Schraner EM , Potgieter C , Borodavka A , Eichwald C , Burrone OR (2020a) Recombinant rotaviruses rescued by reverse genetics reveal the role of NSP5 hyperphosphorylation in the assembly of viral factories. J Virol 94: 1–23 10.1128/JVI.01110-19 PMC6912106 31619556

[embj2021107711-bib-0086] Papa G , Venditti L , Braga L , Schneider E , Giacca M , Petris G , Burrone OR. (2020b) CRISPR‐Csy4‐Mediated Editing of Rotavirus Double‐Stranded RNA Genome. Cell Reports 32: 108205 32997981 10.1016/j.celrep.2020.108205 PMC7523552

[embj2021107711-bib-0087] Patel A , Lee H , Jawerth L , Maharana S , Jahnel M , Hein M , Stoynov S , Mahamid J , Saha S , Franzmann T *et al* (2015) A liquid‐to‐solid phase transition of the ALS protein FUS accelerated by disease mutation. Cell 162: 1066–1077 26317470 10.1016/j.cell.2015.07.047

[embj2021107711-bib-0088] Patton JT , Chen D (1999) RNA‐binding and capping activities of proteins in rotavirus open cores. J Virol 73: 1382–1391 9882343 10.1128/jvi.73.2.1382-1391.1999 PMC103962

[embj2021107711-bib-0089] Patton JT , Silvestri LS , Tortorici MA , Vasquez‐Del Carpio R , Taraporewala ZF (2006) Rotavirus genome replication and morphogenesis: role of the viroplasm. In Reoviruses: Entry, Assembly and Morphogenesis, Roy P (ed), pp 169–187. Berlin, Heidelberg: Springer Berlin Heidelberg 10.1007/3-540-30773-7_6 16909900

[embj2021107711-bib-0090] Patton JT , Spencer E (2000) Genome replication and packaging of segmented double‐stranded RNA viruses. Virology 277: 217–225 11080470 10.1006/viro.2000.0645

[embj2021107711-bib-0091] Petrie BL , Greenberg HB , Graham DY , Estes MK (1984) Ultrastructural localization of rotavirus antigens using colloidal gold. Virus Res 1: 133–152 6099654 10.1016/0168-1702(84)90069-8

[embj2021107711-bib-0092] Pizarro JL , Sandino AM , Pizarro JM , Fernandez J , Spencer E (1991) Characterization of rotavirus guanylyltransferase activity associated with polypeptide VP3. J Gen Virol 72: 325–332 1704411 10.1099/0022-1317-72-2-325

[embj2021107711-bib-0093] Poncet D , Lindenbaum P , L’Haridon R , Cohen J (1997) In vivo and in vitro phosphorylation of rotavirus NSP5 correlates with its localization in viroplasms. J Virol 71: 34–41 8985320 10.1128/jvi.71.1.34-41.1997 PMC191021

[embj2021107711-bib-0094] Ray S , Singh N , Kumar R , Patel K , Pandey S , Datta D , Mahato J , Panigrahi R , Navalkar A , Mehra S *et al* (2020) α‐Synuclein aggregation nucleates through liquid – liquid phase separation. Nat Chem 12: 705–716 32514159 10.1038/s41557-020-0465-9

[embj2021107711-bib-0095] Rhine K , Vidaurre V , Myong S (2020) RNA Droplets. Annu Rev Biophys 49: 247–265 32040349 10.1146/annurev-biophys-052118-115508 PMC7695521

[embj2021107711-bib-0096] Risso‐Ballester J , Galloux M , Cao J , Le Goffic R , Hontonnou F , Jobart‐Malfait A , Desquesnes A , Sake SM , Haid S , Du M *et al* (2021) A condensate‐hardening drug blocks RSV replication in vivo. Nature 595: 596–599 34234347 10.1038/s41586-021-03703-z

[embj2021107711-bib-0097] Roden C , Gladfelter AS (2021) RNA contributions to the form and function of biomolecular condensates. Nat Rev Mol Cell Biol 22: 183–195 32632317 10.1038/s41580-020-0264-6 PMC7785677

[embj2021107711-bib-0098] Saar KL , Morgunov AS , Qi R , Arter WE , Krainer G , Lee AA , Knowles TPJ (2021) Learning the molecular grammar of protein condensates from sequence determinants and embeddings. Proc Natl Acad Sci USA 118: e2019053118 33827920 10.1073/pnas.2019053118 PMC8053968

[embj2021107711-bib-0099] Savastano A , Ibáñez de Opakua A , Rankovic M , Zweckstetter M (2020) Nucleocapsid protein of SARS‐CoV‐2 phase separates into RNA‐rich polymerase‐containing condensates. Nat Commun 11: 6041 33247108 10.1038/s41467-020-19843-1 PMC7699647

[embj2021107711-bib-0100] Schindelin J , Arganda‐Carreras I , Frise E , Kaynig V , Longair M , Pietzsch T , Preibisch S , Rueden C , Saalfeld S , Schmid B *et al* (2012) Fiji: an open‐source platform for biological‐image analysis. Nat Methods 9: 676–682 22743772 10.1038/nmeth.2019 PMC3855844

[embj2021107711-bib-0101] Schnitzbauer J , Strauss MT , Schlichthaerle T , Schueder F , Jungmann R (2017) Super‐resolution microscopy with DNA‐PAINT. Nat Protoc 12: 1198–1228 28518172 10.1038/nprot.2017.024

[embj2021107711-bib-0102] Sen A , Agresti D , Mackow ER (2006) Hyperphosphorylation of the rotavirus NSP5 protein is independent of serine 67 or NSP2 and the intrinsic insolubility of NSP5 is regulated by cellular phosphatases. J Virol 80: 1807–1816 16439537 10.1128/JVI.80.4.1807-1816.2006 PMC1367154

[embj2021107711-bib-0103] Shin Y , Brangwynne CP (2017) Liquid phase condensation in cell physiology and disease. Science 357: eaaf4382 28935776 10.1126/science.aaf4382

[embj2021107711-bib-0104] Silvestri LS , Taraporewala ZF , Patton JT (2004) Rotavirus replication: Plus‐sense templates for double‐stranded RNA synthesis are made in viroplasms. J Virol 78: 7763–7774 15220450 10.1128/JVI.78.14.7763-7774.2004 PMC434085

[embj2021107711-bib-0105] Sotelo PH , Schümann M , Krause E , Chnaiderman J (2010) Analysis of rotavirus non‐structural protein NSP5 by mass spectrometry reveals a complex phosphorylation pattern. Virus Res 149: 104–108 20036292 10.1016/j.virusres.2009.12.006

[embj2021107711-bib-0106] Strauss S , Borodavka A , Papa G , Desiró D , Schueder F , Jungmann R (2021) Principles of RNA recruitment to viral ribonucleoprotein condensates in a segmented dsRNA virus. bioRxiv 10.1101/2021.03.22.435476 [PREPRINT] PMC9925054 36700549

[embj2021107711-bib-0107] Taraporewala ZF , Jiang X , Vasquez‐Del Carpio R , Jayaram H , Prasad BVV , Patton JT (2006) Structure‐function analysis of rotavirus NSP2 octamer by using a novel complementation system. J Virol 80: 7984–7994 16873255 10.1128/JVI.00172-06 PMC1563784

[embj2021107711-bib-0108] Tauber D , Tauber G , Khong A , Van Treeck B , Pelletier J , Parker R (2020) Modulation of RNA condensation by the DEAD‐box protein eIF4A. Cell 180: 411–426 31928844 10.1016/j.cell.2019.12.031 PMC7194247

[embj2021107711-bib-0109] Trask SD , Mcdonald SM , Patton JT (2012) Structural insights into coupling of virion assembly and rotavirus replication. Nat Rev Microbiol 10: 165–177 22266782 10.1038/nrmicro2673 PMC3771686

[embj2021107711-bib-0110] Van Treeck B , Parker R (2018) Emerging roles for intermolecular RNA‐RNA interactions in RNP assemblies. Cell 174: 791–802 30096311 10.1016/j.cell.2018.07.023 PMC6200146

[embj2021107711-bib-0111] Viskovska M , Anish R , Hu L , Chow D‐C , Hurwitz AM , Brown NG , Palzkill T , Estes MK , Prasad BVV (2014) Probing the sites of interactions of rotaviral proteins involved in replication. J Virol 88: 12866–12881 25165107 10.1128/JVI.02251-14 PMC4248930

[embj2021107711-bib-0112] Wang J , Choi J‐M , Holehouse AS , Lee HO , Zhang X , Jahnel M , Maharana S , Lemaitre R , Pozniakovsky A , Drechsel D *et al* (2018) A molecular grammar governing the driving forces for phase separation of prion‐like RNA binding proteins. Cell 174: 688–699 29961577 10.1016/j.cell.2018.06.006 PMC6063760

[embj2021107711-bib-0113] Wei MT , Elbaum‐Garfinkle S , Holehouse AS , Chen CCH , Feric M , Arnold CB , Priestley RD , Pappu RV , Brangwynne CP (2017) Phase behaviour of disordered proteins underlying low density and high permeability of liquid organelles. Nat Chem 9: 1118–1125 29064502 10.1038/nchem.2803 PMC9719604

[embj2021107711-bib-0114] Wheeler JR , Matheny T , Jain S , Abrisch R , Parker R (2016) Distinct stages in stress granule assembly and disassembly. Elife 5: 1–25 10.7554/eLife.18413 PMC5014549 27602576

[embj2021107711-bib-0115] Yamazaki T , Souquere S , Chujo T , Kobelke S , Chong YS , Fox AH , Bond CS , Nakagawa S , Pierron G , Hirose T (2018) Functional domains of NEAT1 architectural lncRNA induce paraspeckle assembly through phase separation. Mol Cell 70: 1038–1053 29932899 10.1016/j.molcel.2018.05.019

