# Supplementary material for: Liquid–liquid phase separation underpins the formation of replication factories in rotaviruses
Source: EMBO J. 2021 Sep 15;40(21):e107711. doi: 10.15252/embj.2021107711 (PMC8561643; doi:10.15252/embj.2021107711)
Supplement: Supplementary file 1 — Expanded View Figures PDF [file EMBJ-40-e107711-s012.pdf]

Expanded View Figures

Figure EV1. Viroplasms are sensitive to aliphatic diols during RV infection and require expression of NSP5.

A RV-infected MA104-NSP5-EGFP cells (4 HPI) fixed with 4% (v/v) paraformaldehyde for 5 min (PFA-fixed, left panel). Application of 1,6HD (5% v/v) does not dissolve NSP5-EGFP granules after chemical cross-linking with PFA (right).

B Live-cell images of RV-infected MA104-NSP2-mCherry cells at 4 HPI, shown in Fig 1. NSP2-mCherry-tagged replication factories dissolve upon application of 4% (v/v) 1,6HD (Movie EV2).

C Immunofluorescent (IF) staining of viral replication factories in RV-infected MA104 cells 6 HPI, before (left) and after a brief (5 min) application of 4% 1,6HD or propylene glycol (PG), respectively, prior to PFA fixation and IF detection of NSP5 (red). Nuclei are stained with DAPI (blue).

D Recombinant rotavirus NSP5-KO (NSP5 knockout) infection of MA104-derived stable cell lines producing NSP5-EGFP (*left*), NSP2-mCherry (*middle*) and the wild type NSP5 (*right*, NSP5-rich condensates, IF staining). All cells were fixed and imaged 8 h after infection with NSP5-KO RV.

Data information: Scale bar, 10 μm.

© 2021 The Authors

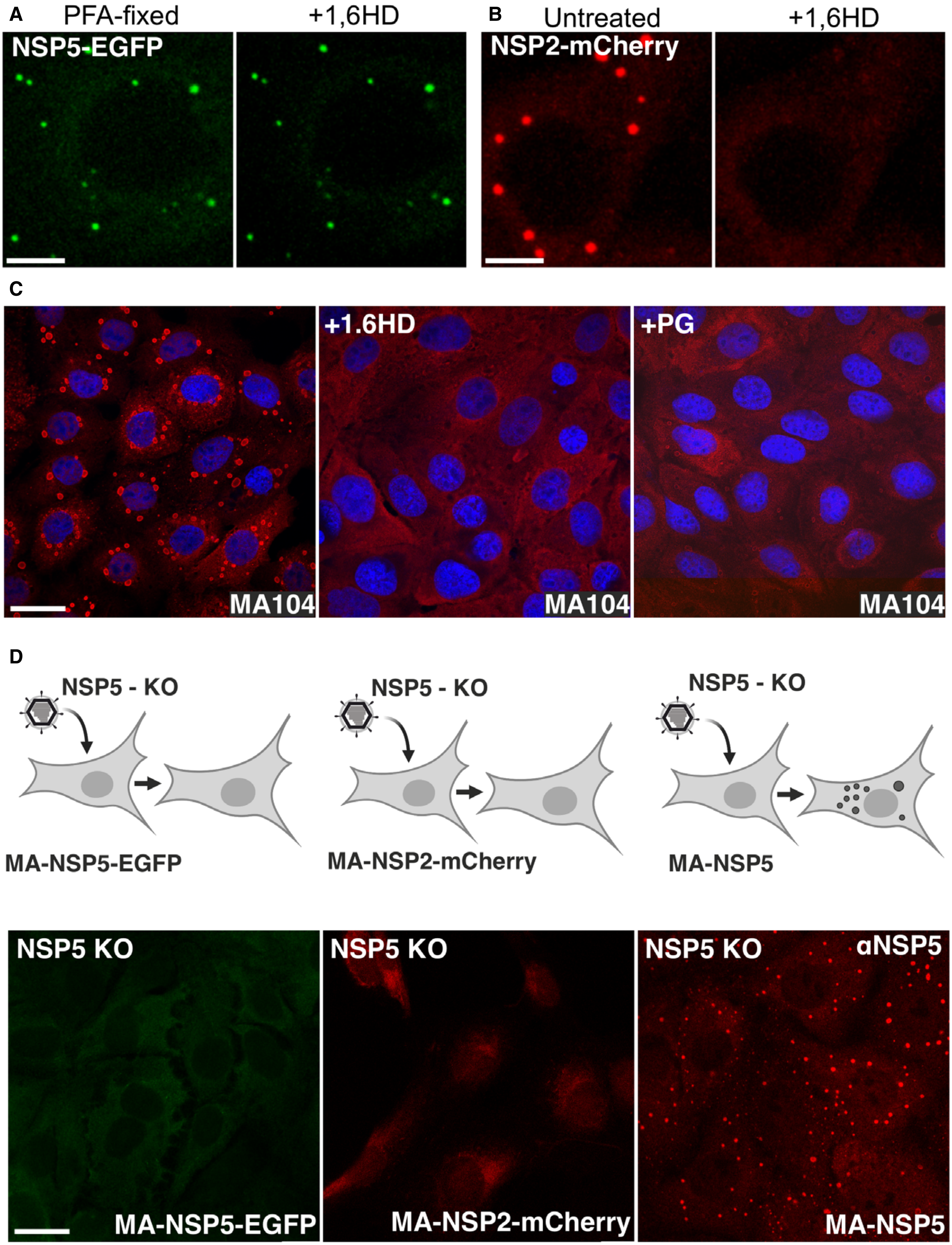

Figure EV1.

 © 2021 The Authors

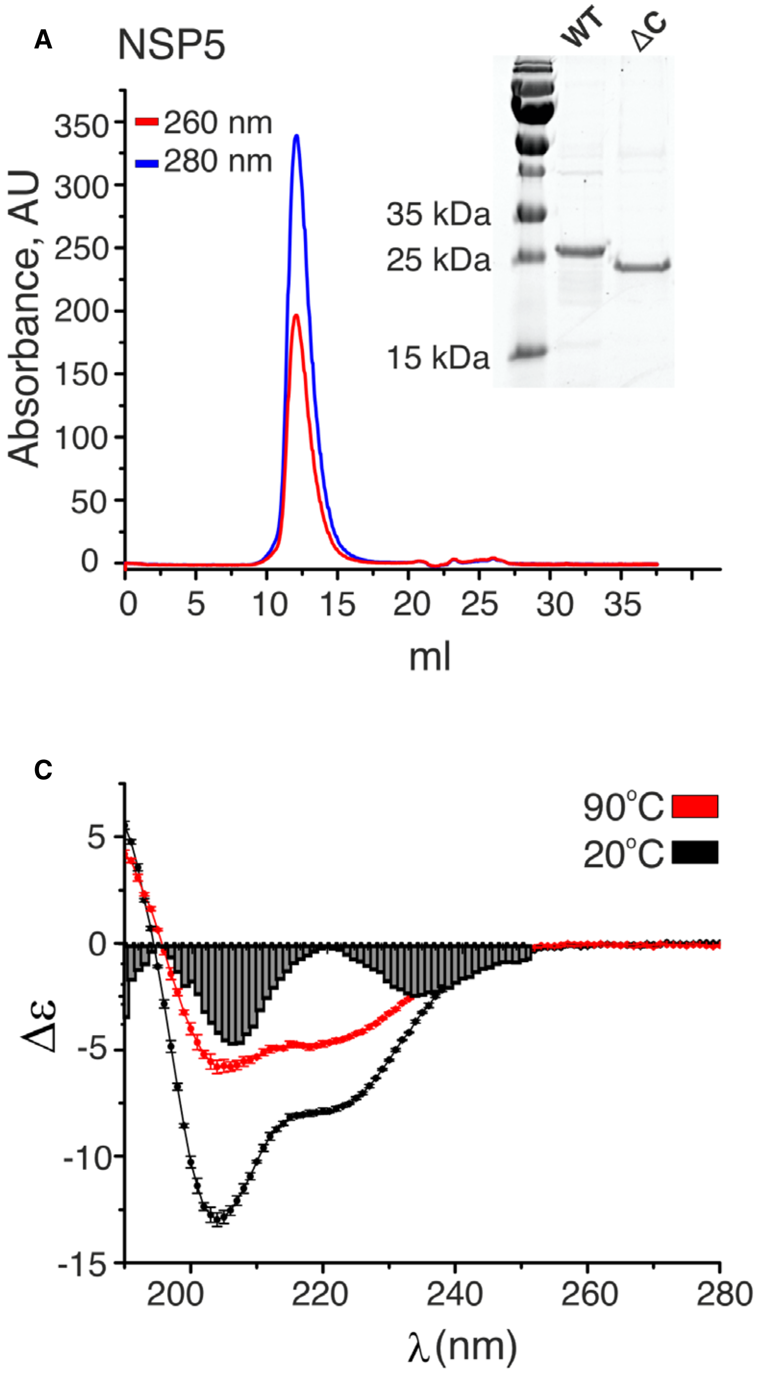

Figure EV2. Solution characterisation of NSP5.

A Size-exclusion (SEC) analysis (Superdex 200 Increase 10/300 GL) of the purified recombinant protein NSP5. After purification and refolding, the protein was monodisperse and free of nucleic acids, as judged by the A_{260}/A_{280} ratio (blue trace—absorbance at 280 nm, red trace—absorbance at 260 nm). Inset—SDS–PAGE analysis of purified NSP5 and its C-terminal truncation variant (ΔC).

B Quasi-elastic scattering analysis of the SEC peak fraction shown in (A). A non-linear fit of the data (red line) indicates an estimated hydrodynamic radius, R_h ~ 6.8 nm. 5 autocorrelation functions (ACFs) were calculated for 60 s measurement per protein sample, with errors representing SD values from ACFs. Data were recorded to confirm the oligomeric state of NSP5, which is consistent with the SEC trace shown in (A).

C Circular dichroism (CD) spectra of NSP5 acquired at 20°C (black) and after thermal denaturation at 90°C (red). 3 scans were acquired and averaged per sample for each temperature (each point represents mean ± SD). Secondary structure analysis of NSP5 determined by spectral deconvolution of the CD spectra recorded at 20°C (black) and after the thermal denaturation (red). H—helices, B—β-sheets, T—turns, O—disordered. BestSel fit residuals are shown for region 190–240 nm along the x-axis.

Source data are available online for this figure.

© 2021 The Authors

Figure EV3. LLPS of NSP5 *in vitro* and quantitative characterisation of LLPS using PhaseScan approach.

A NSP2-488 + untagged NSP5 (10 μM NSP5 + 5 μM NSP2) at physiological salt concentration (*left*) and in the presence of 0.5 M NaCl (*right*). Scale bar, 10 μm.

B *Left*: Untagged NSP5 (35 μM) spiked with NSP5-647 incubated with 5 μM poly-lysine (average MW 70 kDa, polyK); *Right*: 75 μM NSP5 + 5 μM poly-lysine. Scale bar, 10 μm.

C Schematics of the droplet-generating device. Droplets were generated using a microfluidic device controlled by automated syringe pumps. Combination of aqueous droplet components prior to the droplet-generating junction (inset) enables variation in droplet solution composition. Droplets are collected (6 min collection time) off-chip, before undergoing analysis by epifluorescence microscopy. Inset i—flow profile for NSP2 and NSP5 concentrations as produced by automated flow control in droplet generation. Flow set points (black squares) are maintained for 7 s, with the overall flow programme lasting 168 s. The arrow indicates the beginning of the continuous flow programme loop.

 © 2021 The Authors

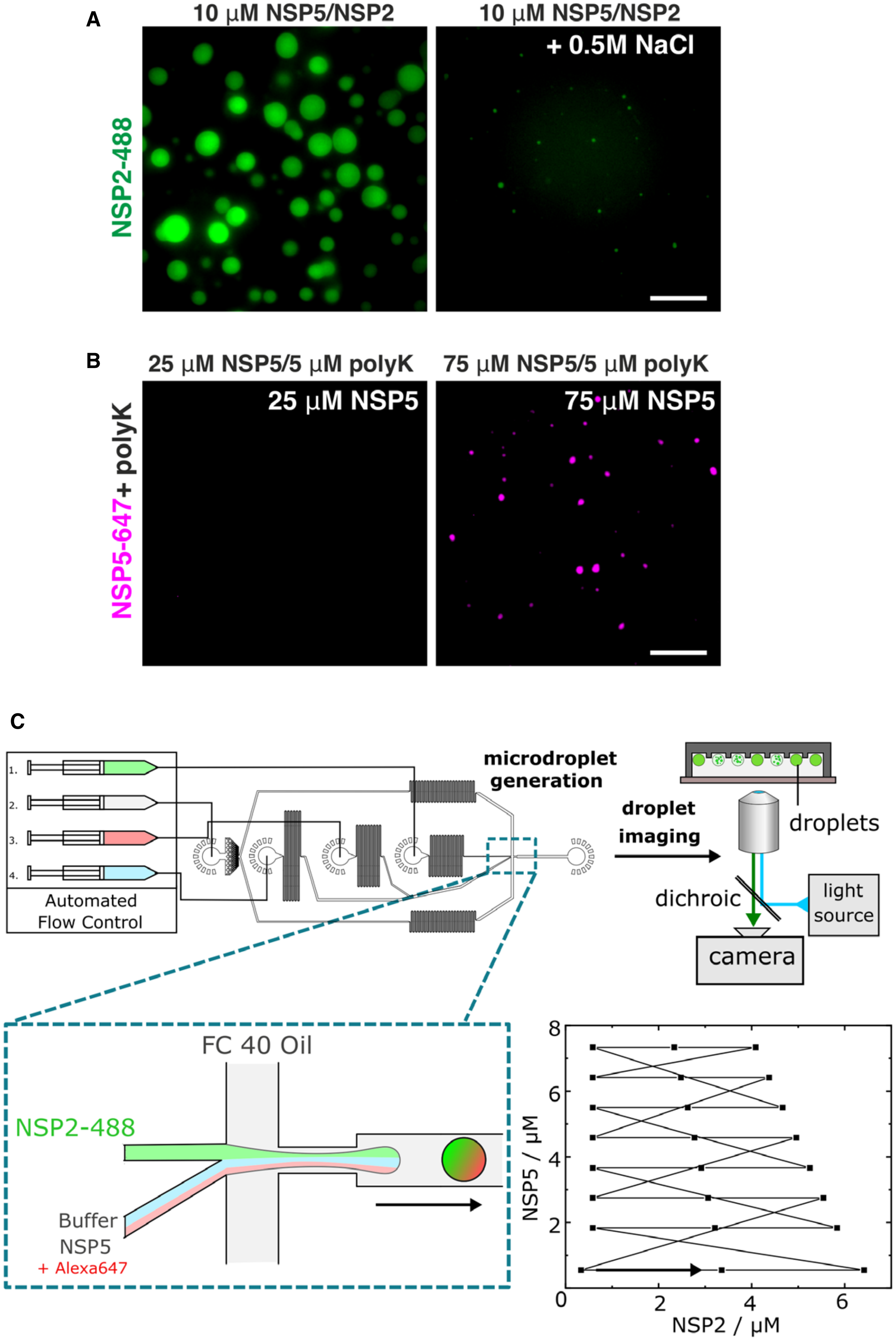
A
10 µM NSP5/NSP2
10 µM NSP5/NSP2
+ 0.5M NaCl
NSP2-488
B
25 µM NSP5/5 µM polyK
75 µM NSP5/5 µM polyK
25 µM NSP5
75 µM NSP5
NSP5-647+ polyK
C
1.
2.
3.
4.
Automated
Flow Control
microdroplet
generation
droplet
imaging
droplets
dichroic
light
source
camera
FC 40 Oil
NSP2-488
Buffer
NSP5
+ Alexa647
NSP5 / µM
NSP2 / µM
0
2
4
6
8
0
2
4
6

Figure EV3.

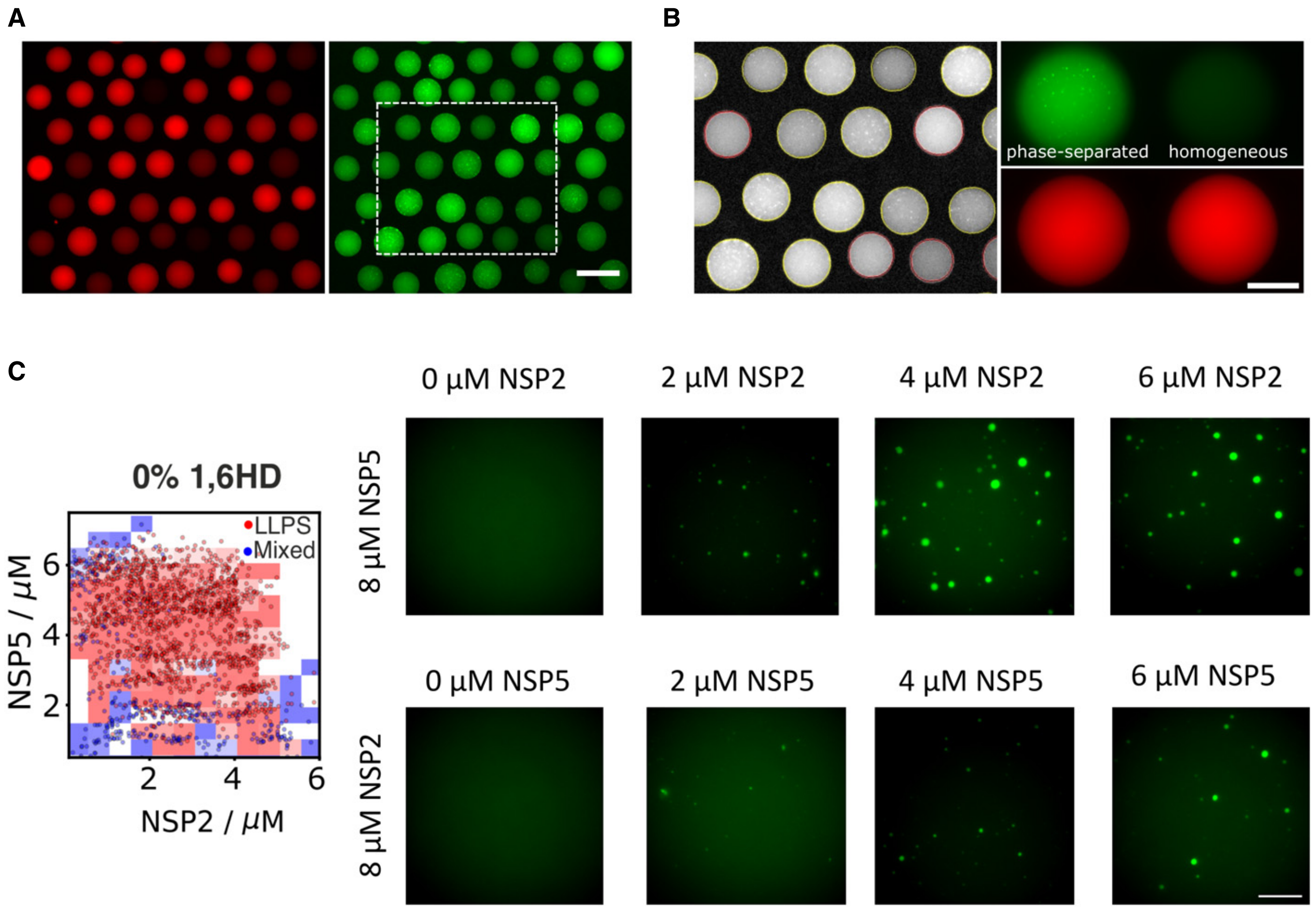

Figure EV4. PhaseScan analysis of NSP5/NSP2 coacervates *in vitro* and quantitation of NSP5 expression during RV infection.

A, B Representative epifluorescence data for 0% 1,6-hexanediol phase diagram of trapped microdroplets and barcode fluorescence imaged in 488 nm (*left*) and 647 nm (*right*) channels. Scale bar, 200 μm. (B) Fit of droplet outlines and phase separation classification output for region enclosed by dashed box in (A), red and yellow outlines denote droplet classification as homogeneous and phase-separated, respectively. Representative images of microdroplets and 647-dye-labelled barcode fluorescence classified as phase-separated (*left*) and homogenous (*right*) imaged in 488 nm (upper panel) and 647 nm (lower panel) channels. Scale bar, 100 μm.

C PhaseScan-generated phase diagram of the NSP5/NSP2 mix (*left panel*), with representative droplet images of NSP5/NSP2 condensates formed with 2–8 μM protein. Scale bar, 10 μm.

Source data are available online for this figure.

 © 2021 The Authors

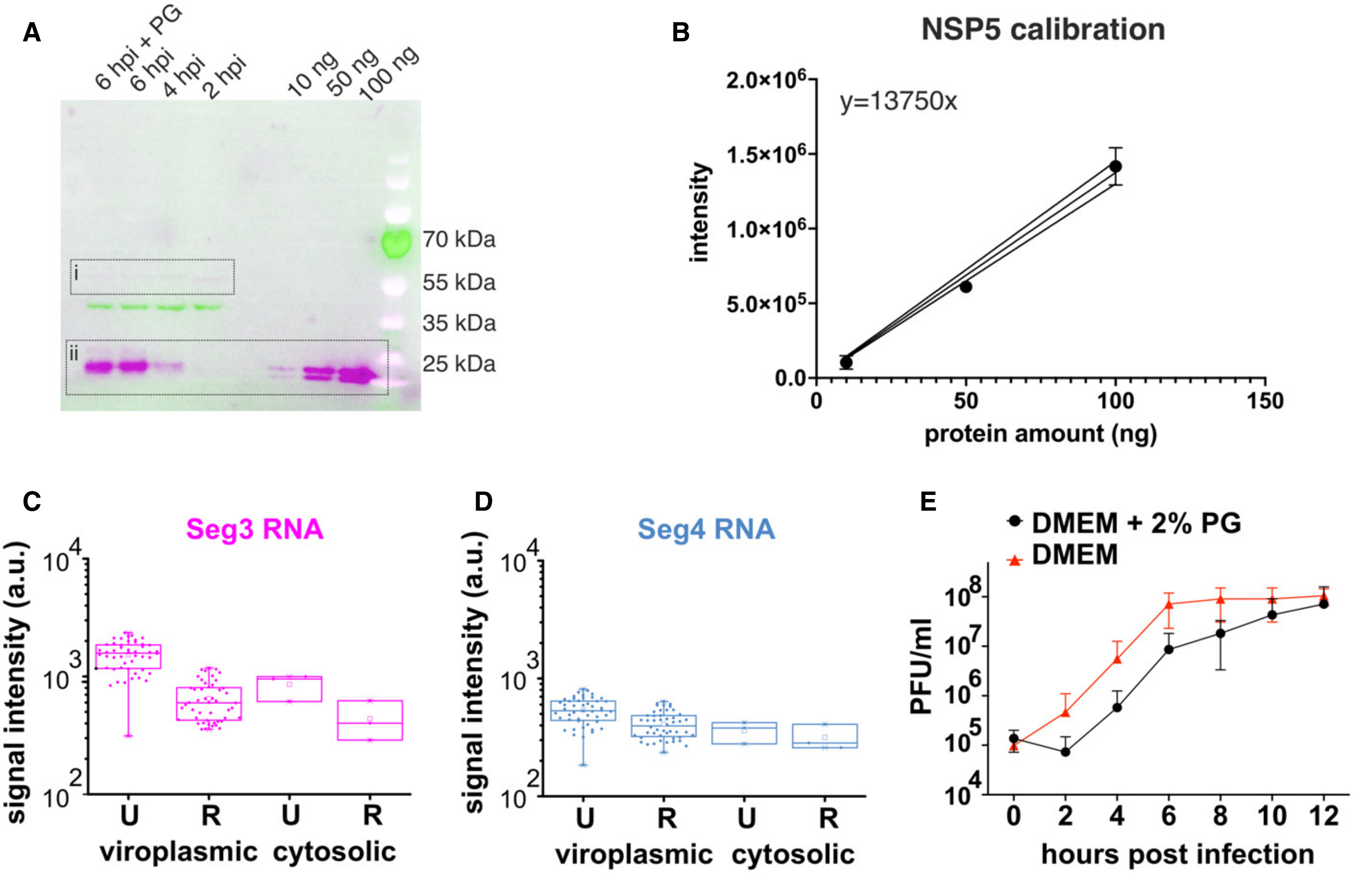

Figure EV5. Effects of propylene glycol on rotavirus replication.

A Western blot quantification of NSP5 produced in RV-infected cells (MOI = 10) harvested at 2, 4 and 6 HPI. 1 × 10^6 RV-infected MA-NSP5-EGFP cells were harvested (see Materials and Methods), and 10% of each total cell lysate sample were loaded on a 12% SDS–PAGE gel, along with recombinant N-His-tagged and untagged NSP5 standards of known concentrations (10–100 ng). NSP5 was detected using anti-NSP5 antibodies, as described in Materials and Methods. NSP5 signal (DyLight800 anti-guinea pig, magenta, box ii) was detected simultaneously with b-actin (hFAB Rhodamine Anti-Actin, green) for sample loading normalisation purposes. Note a very low level of NSP5-EGFP expression detected by NSP5-specific antibodies (box i). The low-level expression of NSP5-EGFP remains constant across the infection course, while only the virally expressed NSP5 levels increase between 2 and 6 HPI. NSP5 amount in RV-infected cells treated with 4.7% propylene glycol ("6 hpi + PG," 15 min treatment) is similar to that produced at 6 HPI in untreated cells.

B Quantification of the Western blot data shown in (A). Integrated band intensities for NSP5 samples of known concentrations were determined using Chemidoc MP Imaging system and plotted as mean ± SD values. Linear regression analysis fit (solid black line) shown along with 95% CI (solid lines) was used to determine the amount of NSP5 produced at 2, 4 and 6 HPI. Quantification for each point was carried out three times, each representing technical replicates.

C, D Changes in the localisation of Seg3 and Seg4 RNAs and their relative distribution between the viroplasms and the cytosol before (Untreated, U) and 15 min after PG treatment (Recovery, R). Median and quartile values of integrated signal intensities (normalised by area) for each channel for viroplasms 2 ("viroplasmic"), and individual cells (*N* = 9, "cytosolic") are shown, data represent technical replicates (individual RV-infected cells). Box plots represent the $25^{th}/75^{th}$ interquartile range, with whiskers representing the $5^{th}/95^{th}$ percentile values. Medians shown as central bands, and means shown as squares. Crosses denote 1% and 99% percentile values, and minimum and maximum values are shown as dashes.

E Rotavirus replication kinetics (strain SA11) in MA104 cells adapted to grow in the presence of 2% PG. Infection was carried out in the presence of 2% PG (black) and a standard PG-free medium (red). 2% PG was added 1 h after virus absorption (MOI of 10). Virus titres are expressed in PFU/ml, and each measurement represents a mean ± SD values estimated for three independent repeats.

Source data are available online for this figure.

© 2021 The Authors
