# Supplementary material for: Liquid–liquid phase separation underpins the formation of replication factories in rotaviruses
Source: EMBO J. 2021 Sep 15;40(21):e107711. doi: 10.15252/embj.2021107711 (PMC8561643; doi:10.15252/embj.2021107711)
Supplement: Supplementary file 2 — Movie EV1 [file EMBJ-40-e107711-s009.zip › Movie_EV1_Legend.docx]

**Movie Legend EV1.** Live-cell confocal imaging of MA-NSP5-EGFP cells infected with rotavirus (MOI 10) at 5 HPI. Cell culture medium was briefly supplemented with 4% (v/v) 1,6-hexanediol (disappearance of EGFP granules), then removed 60 s later, and replaced with fresh cell culture medium without 1.6 hexanediol to allow reformation of droplets. Maximum intensity projections of EGFP granules were used to assemble the file containing 30 frames in total.
