# Supplementary material for: Liquid–liquid phase separation underpins the formation of replication factories in rotaviruses
Source: EMBO J. 2021 Sep 15;40(21):e107711. doi: 10.15252/embj.2021107711 (PMC8561643; doi:10.15252/embj.2021107711)
Supplement: Supplementary file 3 — Movie EV2 [file EMBJ-40-e107711-s003.zip › Movie_EV2_Legend.docx]

**Movie Legend EV2.** Live-cell imaging of MA-NSP2-mCherry cells infected with rotavirus (MOI 10) at 5 HPI. Cell culture medium was briefly supplemented with 4% (v/v) 1,6-hexanediol (disappearance of NSP2-mCherry tagged granules upon application of 1.6HD).
