# Supplementary figures and images for: Liquid–liquid phase separation underpins the formation of replication factories in rotaviruses
Source: EMBO J. 2021 Sep 15;40(21):e107711. doi: 10.15252/embj.2021107711 (PMC8561643; doi:10.15252/embj.2021107711)

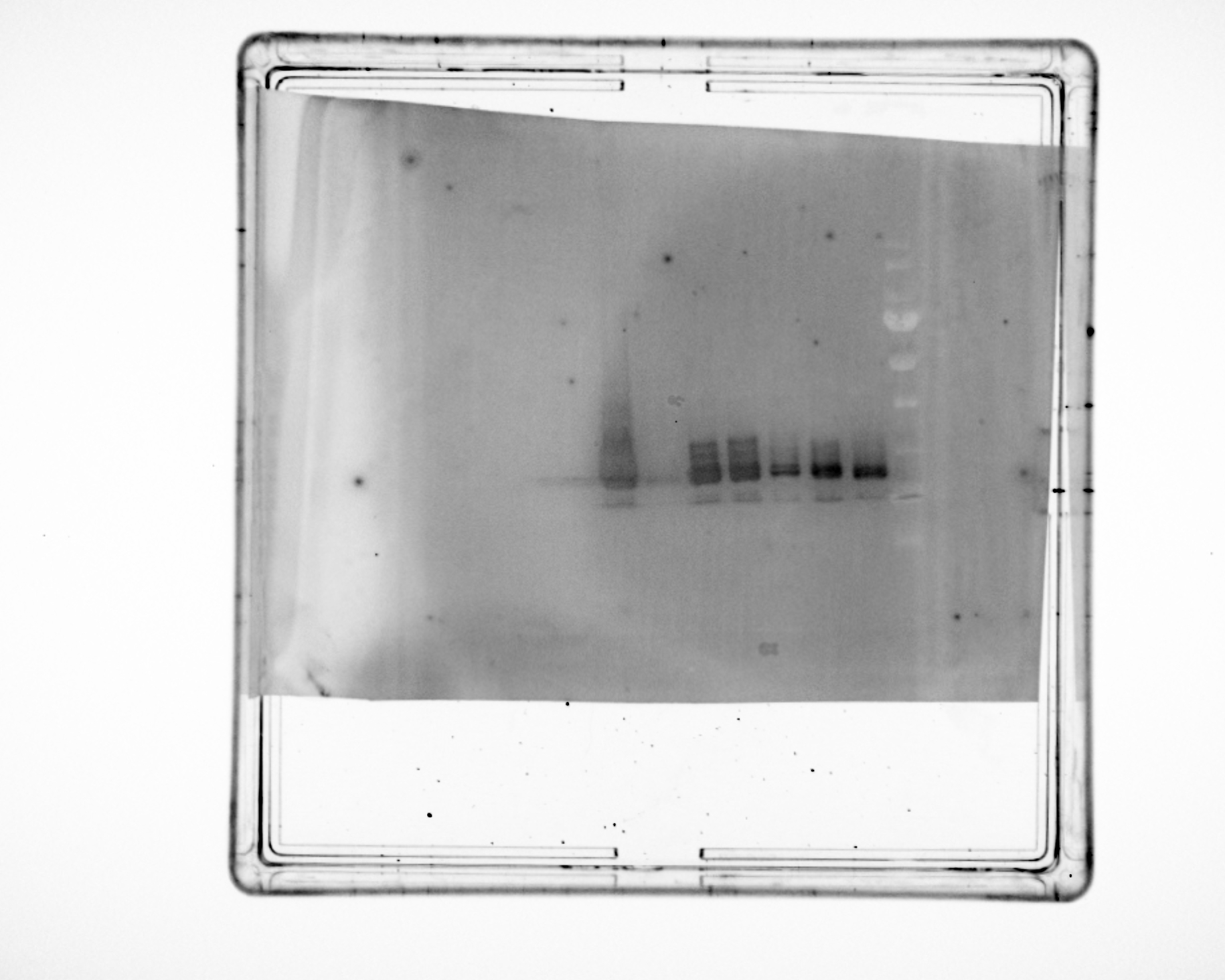

Supplement: Supplementary file 10 — Source Data for Figure 6 [file EMBJ-40-e107711-s005.zip › Fig6f_WB/NSP5.jpg]

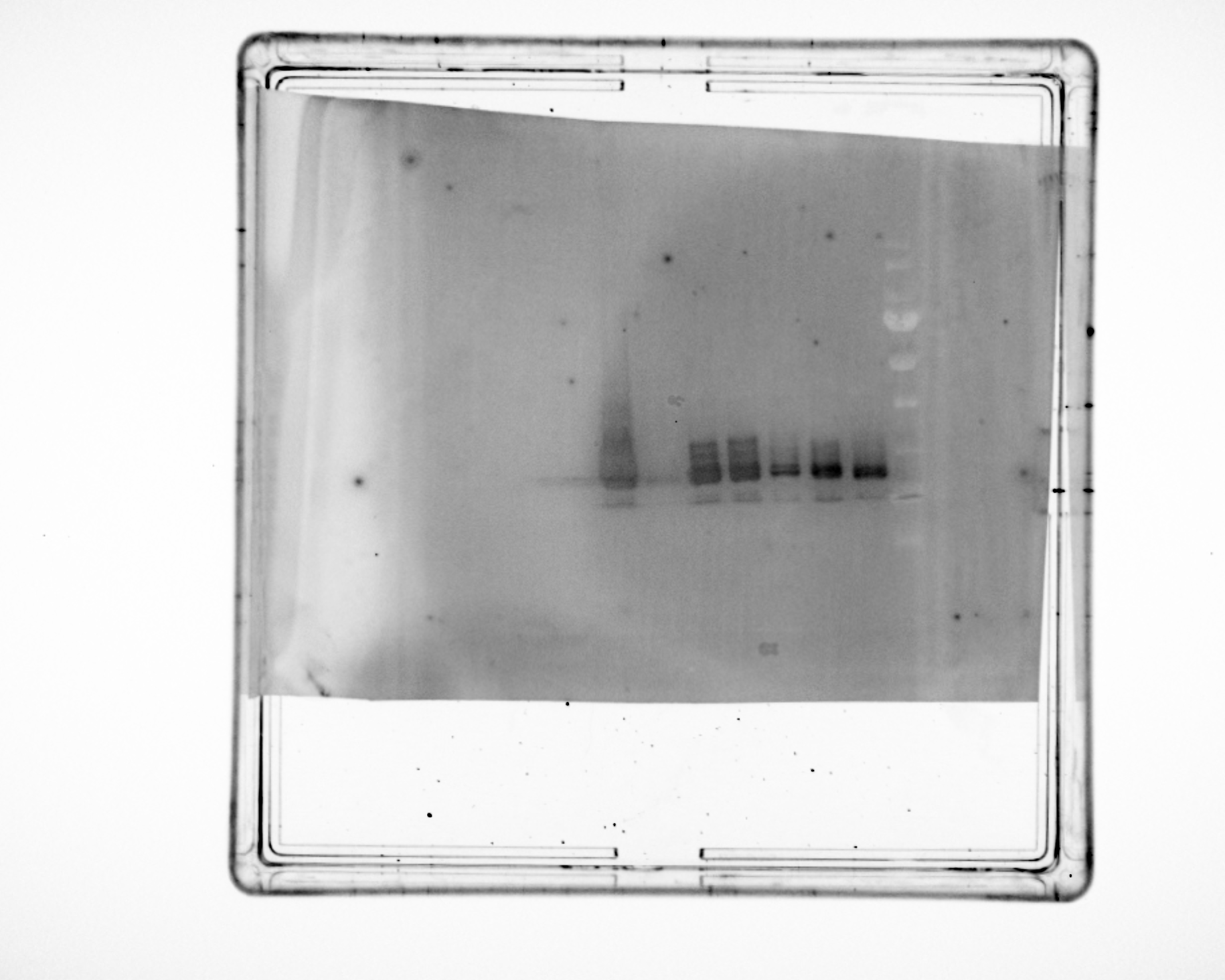

Supplement: Supplementary file 10 — Source Data for Figure 6 [file EMBJ-40-e107711-s005.zip › Fig6f_WB/NSP5.tif]

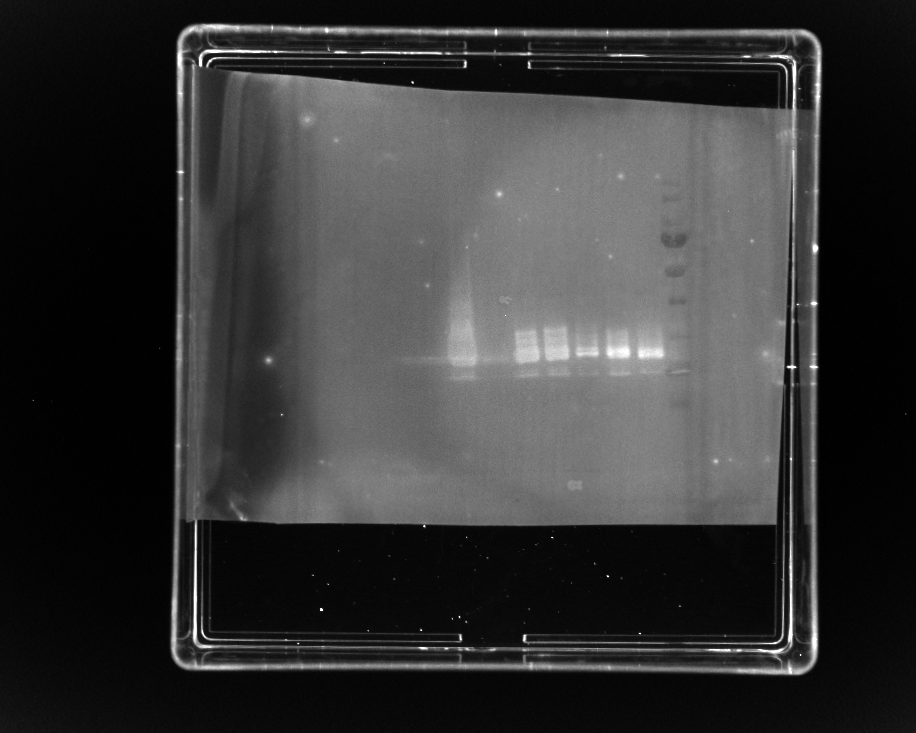

Supplement: Supplementary file 10 — Source Data for Figure 6 [file EMBJ-40-e107711-s005.zip › Fig6f_WB/NSP5_raw.tif]

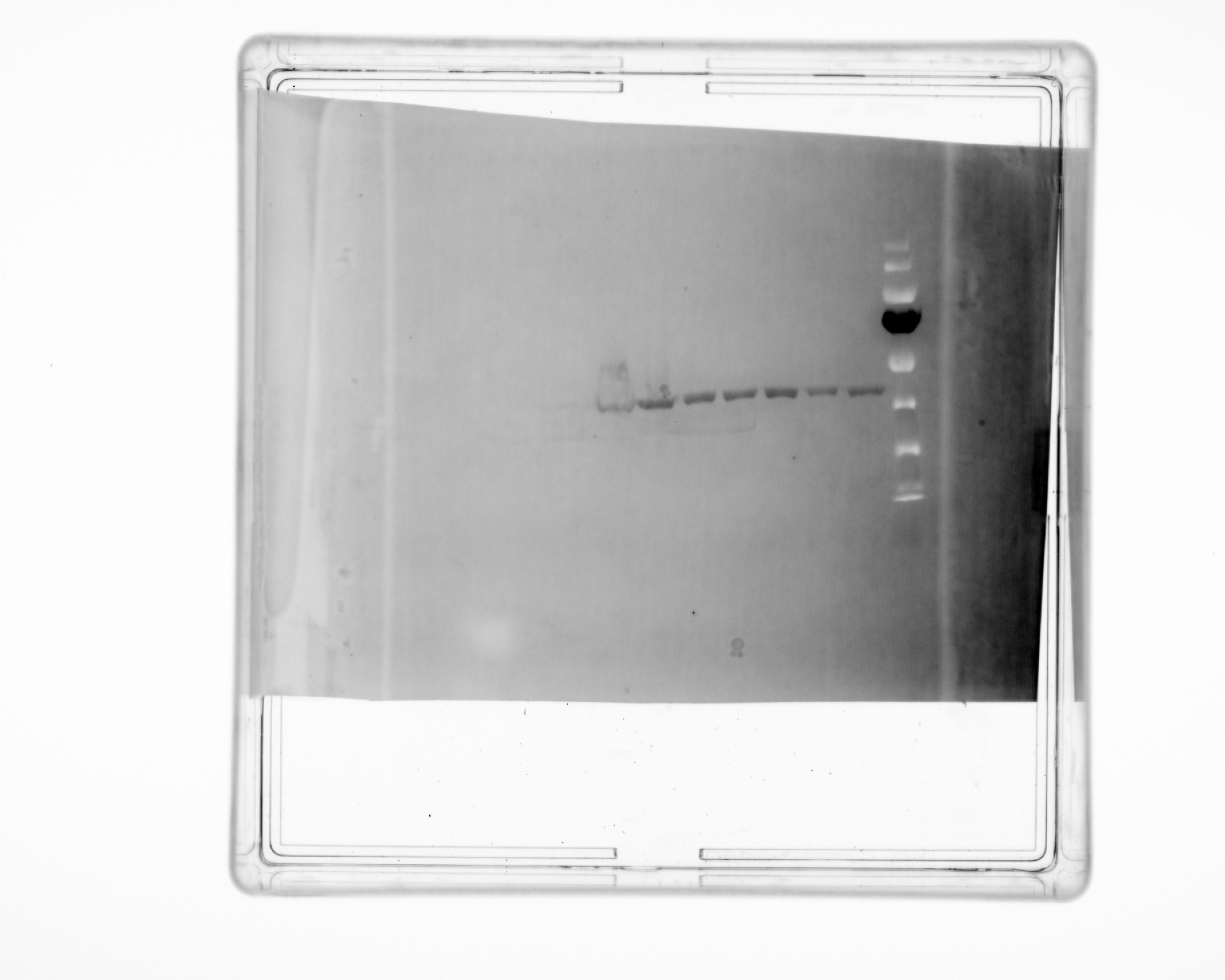

Supplement: Supplementary file 10 — Source Data for Figure 6 [file EMBJ-40-e107711-s005.zip › Fig6f_WB/Rhodamine.jpg]

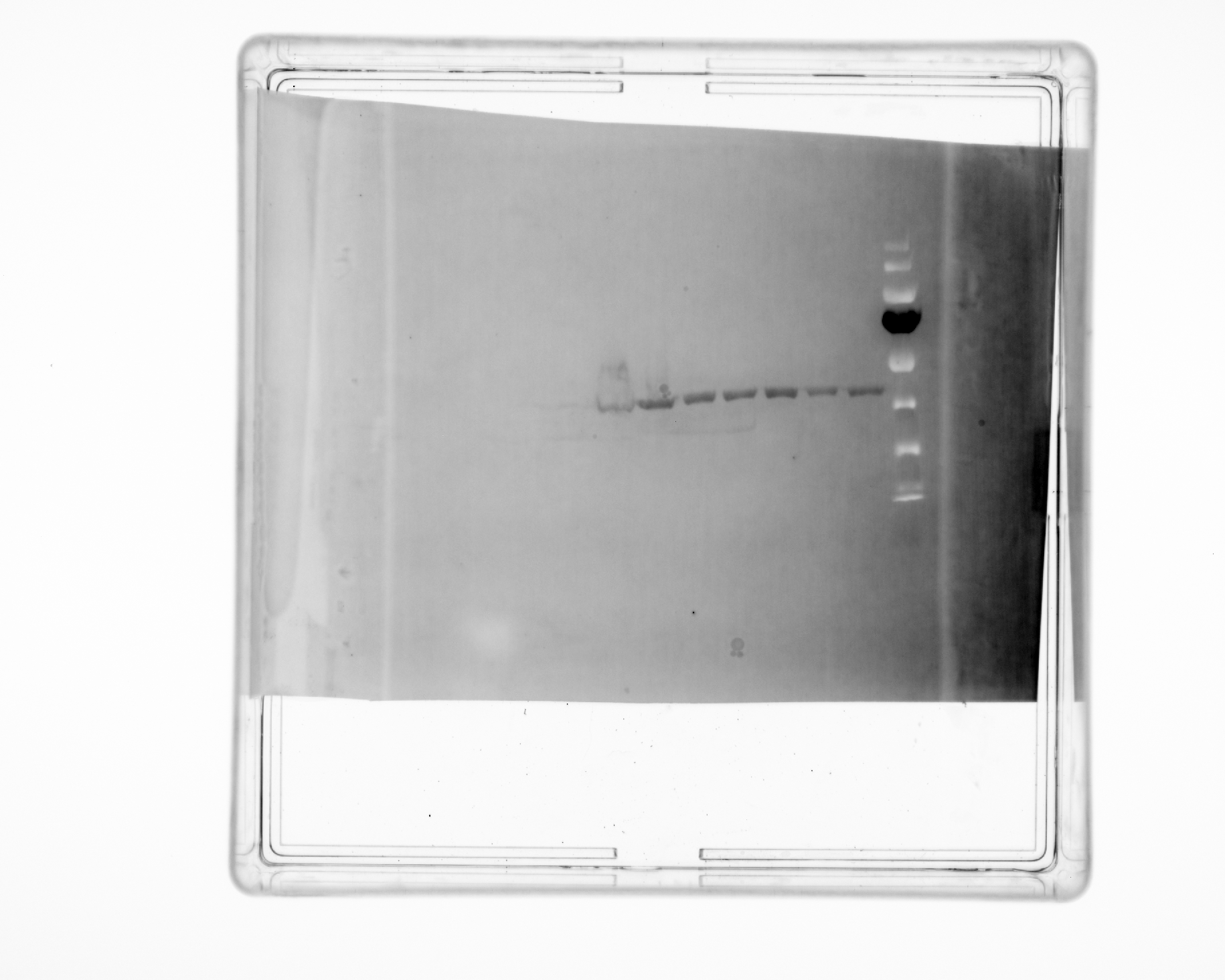

Supplement: Supplementary file 10 — Source Data for Figure 6 [file EMBJ-40-e107711-s005.zip › Fig6f_WB/Rhodamine.tif]

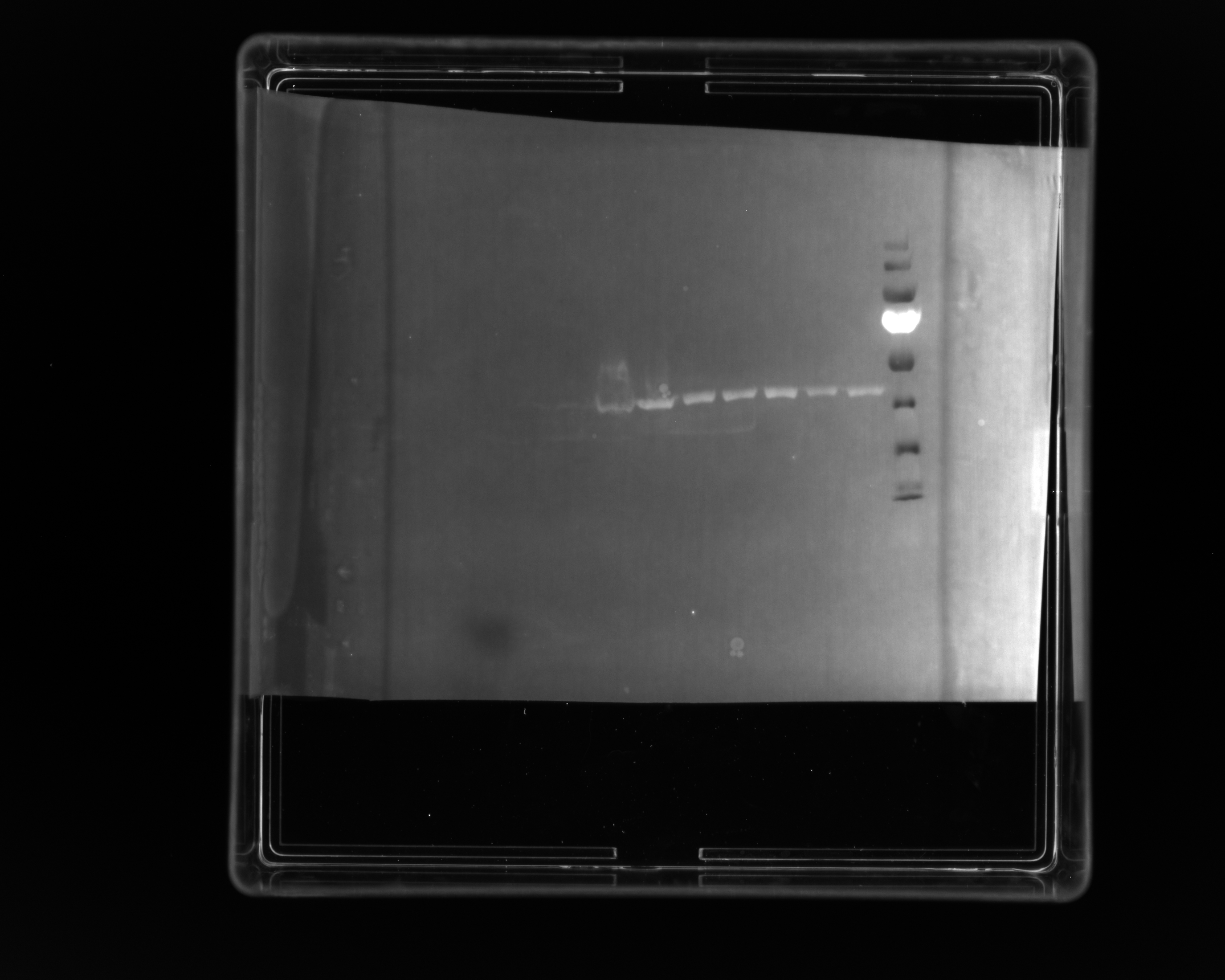

Supplement: Supplementary file 10 — Source Data for Figure 6 [file EMBJ-40-e107711-s005.zip › Fig6f_WB/Rhodamine_raw.tif]

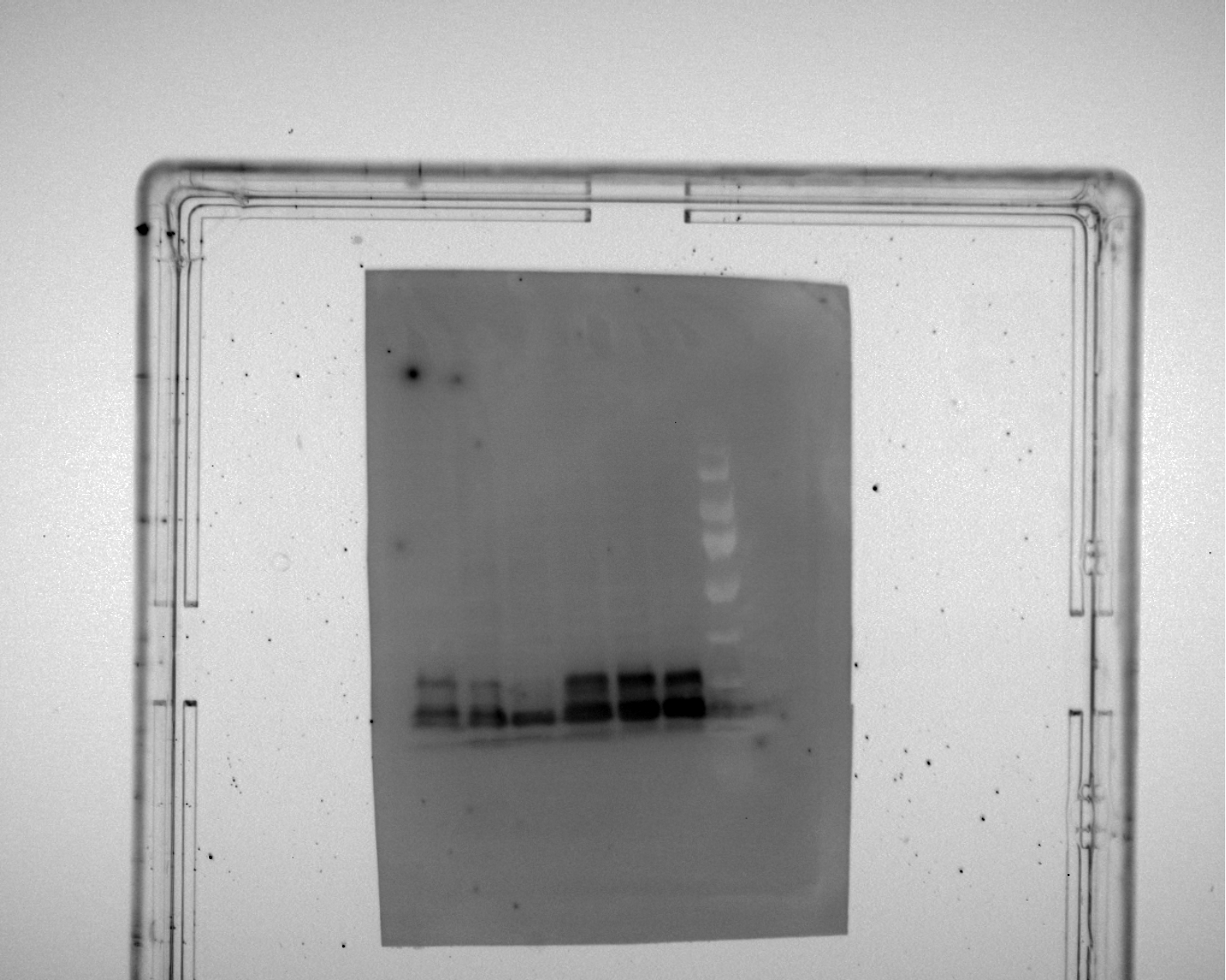

Supplement: Supplementary file 10 — Source Data for Figure 6 [file EMBJ-40-e107711-s005.zip › Fig6g_WB/ADMIN_2021-05-21_19h08m34s(DyLight_800).jpg]

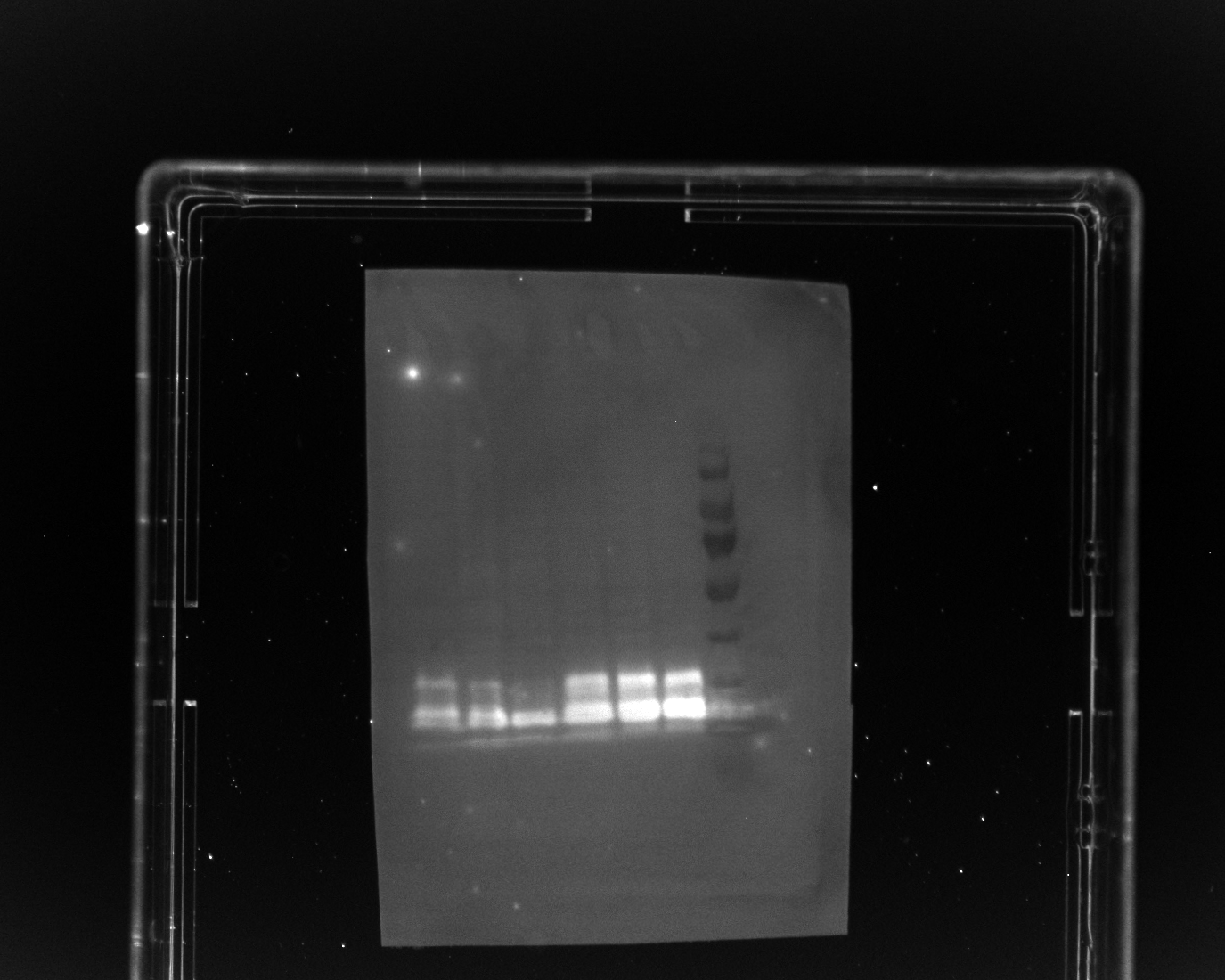

Supplement: Supplementary file 10 — Source Data for Figure 6 [file EMBJ-40-e107711-s005.zip › Fig6g_WB/ADMIN_2021-05-21_19h08m34s(DyLight_800).raw16.tif]

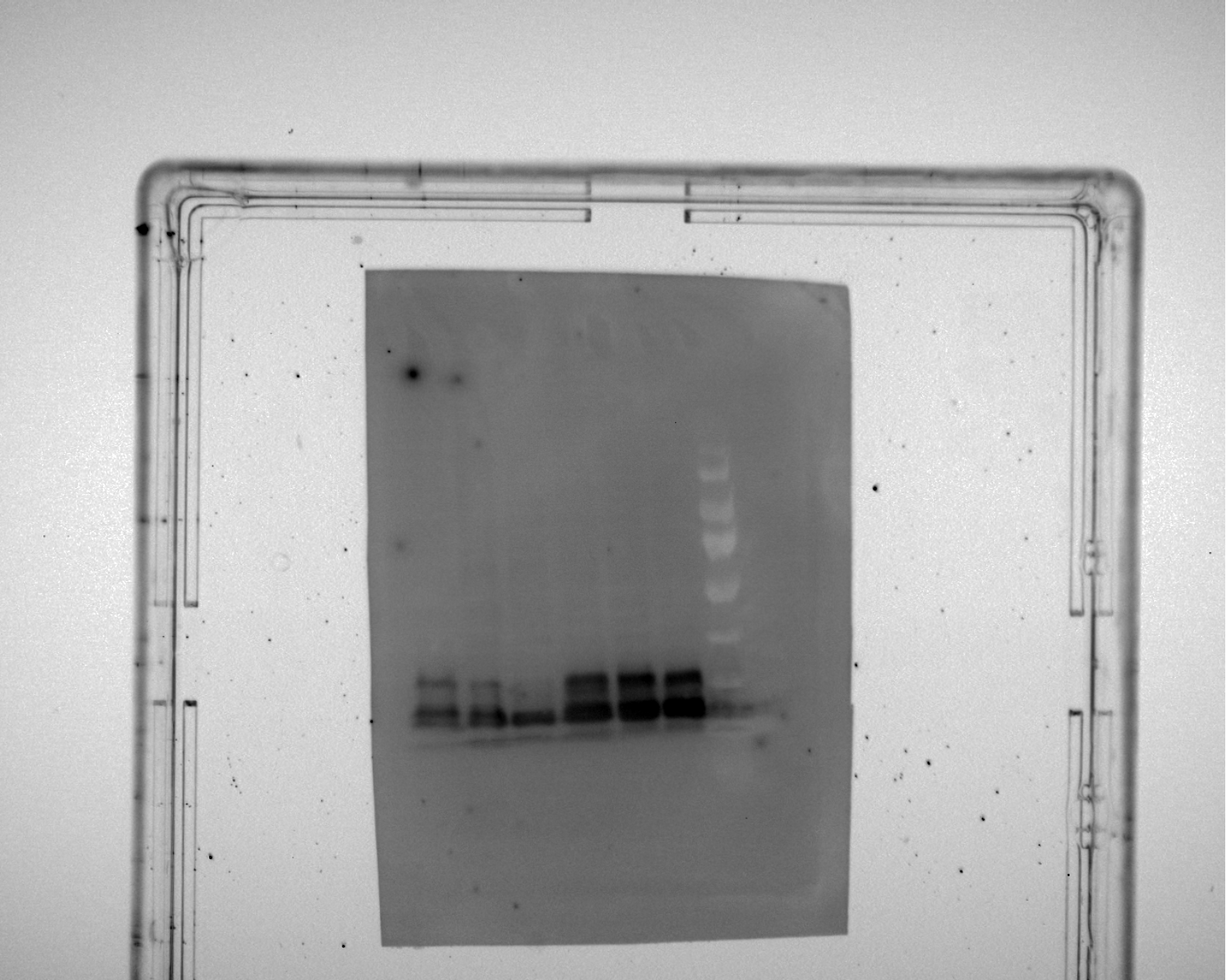

Supplement: Supplementary file 10 — Source Data for Figure 6 [file EMBJ-40-e107711-s005.zip › Fig6g_WB/ADMIN_2021-05-21_19h08m34s(DyLight_800).tif]

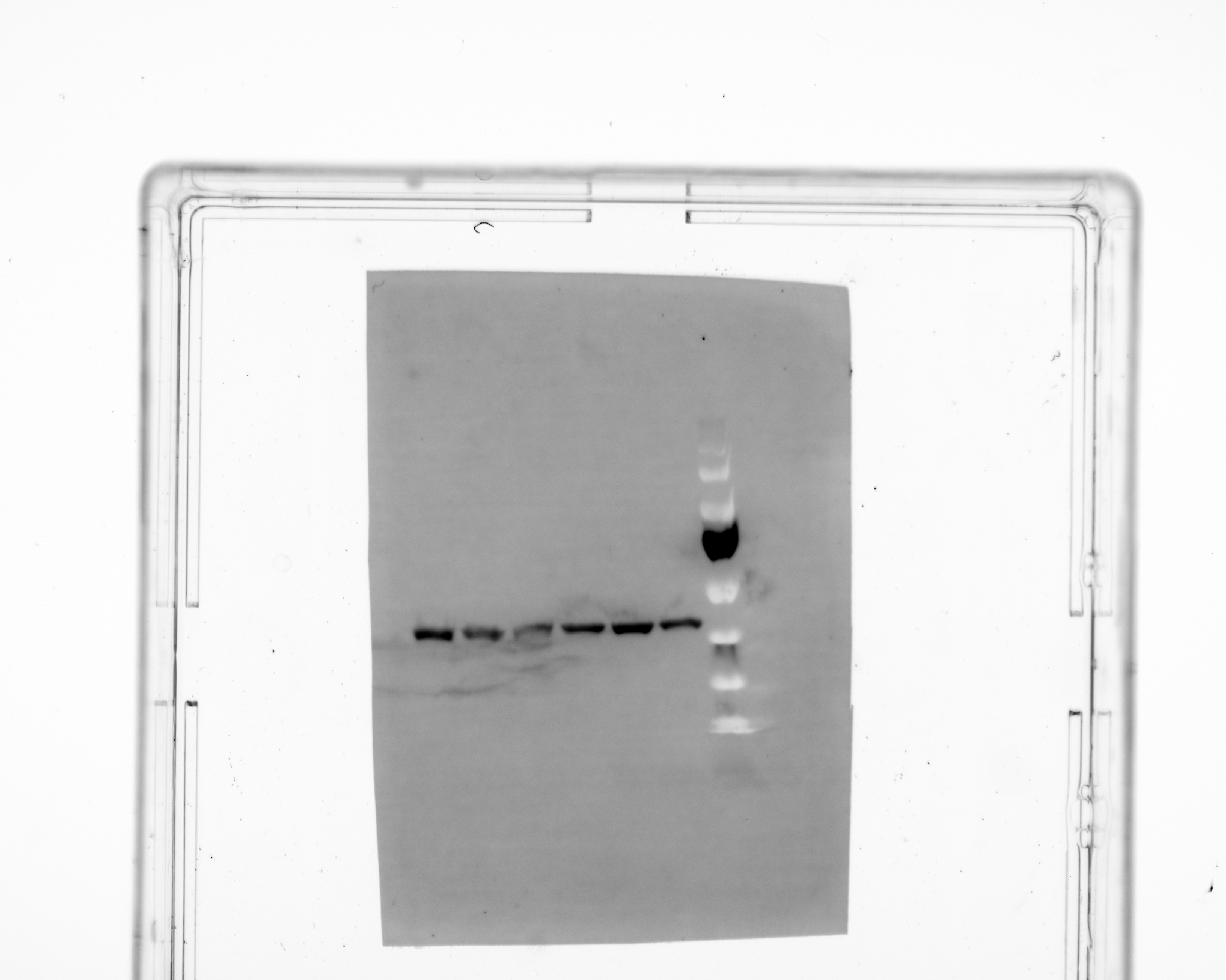

Supplement: Supplementary file 10 — Source Data for Figure 6 [file EMBJ-40-e107711-s005.zip › Fig6g_WB/ChemiDoc_Images_2021-05-21_19.30.11_Rhodamin_b-actin/ADMIN_2021-05-21_19h09m33s(Rhodamine).jpg]

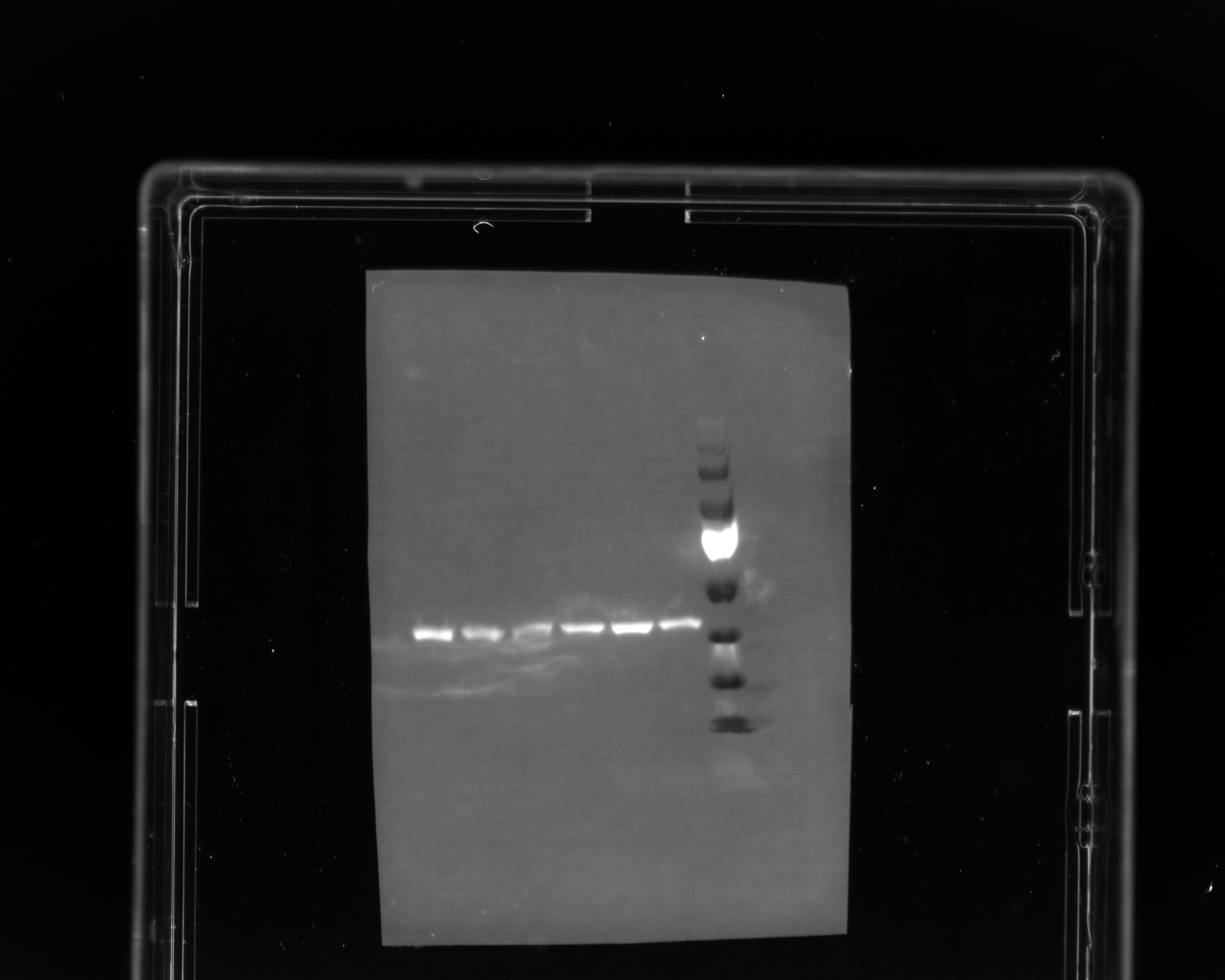

Supplement: Supplementary file 10 — Source Data for Figure 6 [file EMBJ-40-e107711-s005.zip › Fig6g_WB/ChemiDoc_Images_2021-05-21_19.30.11_Rhodamin_b-actin/ADMIN_2021-05-21_19h09m33s(Rhodamine).raw16.tif]

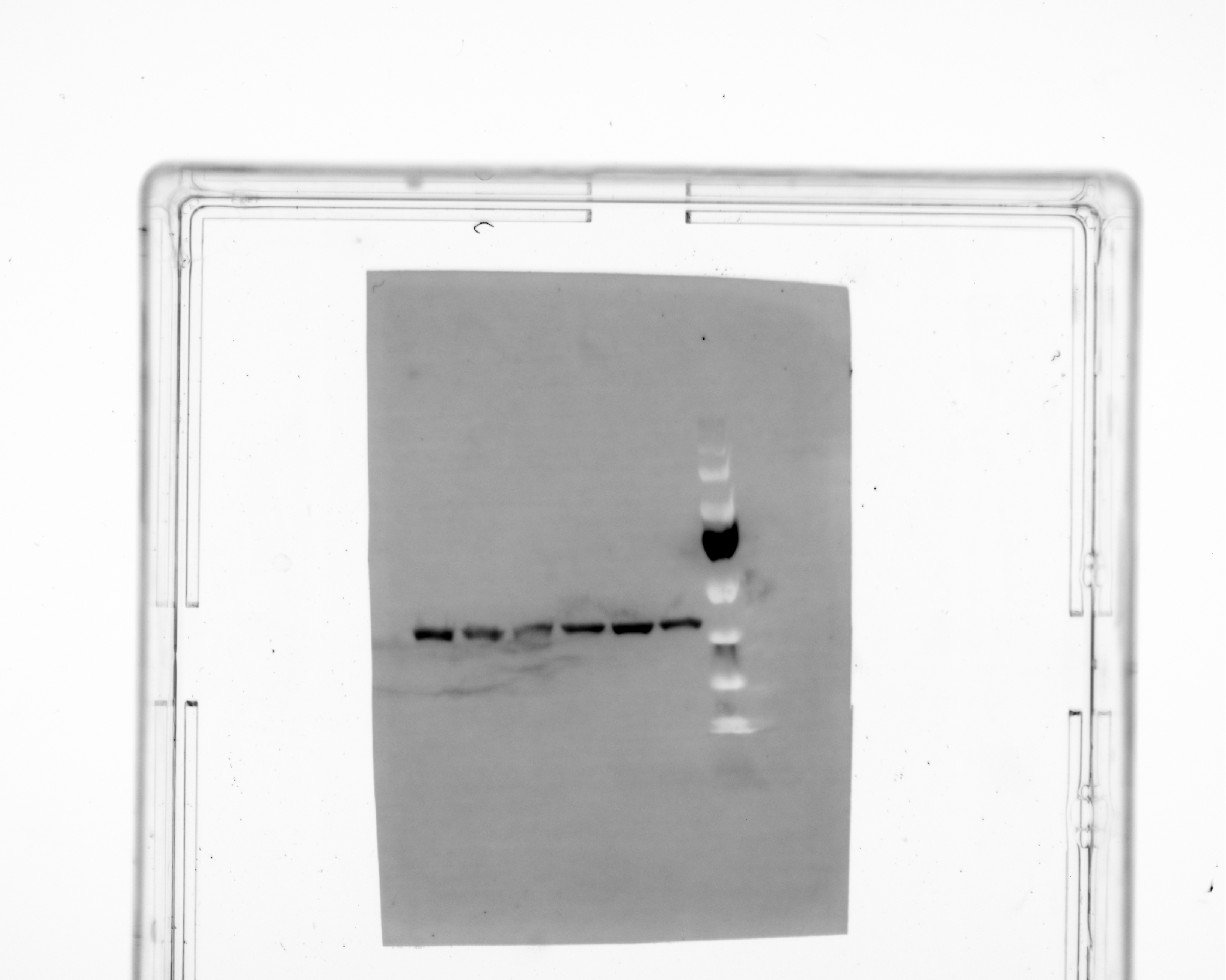

Supplement: Supplementary file 10 — Source Data for Figure 6 [file EMBJ-40-e107711-s005.zip › Fig6g_WB/ChemiDoc_Images_2021-05-21_19.30.11_Rhodamin_b-actin/ADMIN_2021-05-21_19h09m33s(Rhodamine).tif]
