# Supplementary material for: Liquid–liquid phase separation underpins the formation of replication factories in rotaviruses
Source: EMBO J. 2021 Sep 15;40(21):e107711. doi: 10.15252/embj.2021107711 (PMC8561643; doi:10.15252/embj.2021107711)
Supplement: Supplementary file 12 [file EMBJ-40-e107711-s002.pdf]

THE EMBO JOURNAL

Liquid-Liquid Phase Separation Underpins the Formation of Replication Factories in Rotaviruses

Florian Geiger, Julia Acker, Guido Papa, Xinyu Wang, William E. Arter, Kadi L. Saar, Nadia A. Erkamp, Runzhang Qi, Jack P.K. Bravo, Sebastian Strauss, Georg Krainer, Oscar R. Burrone, Ralf Jungmann, Tuomas P.J. Knowles, Hanna Engelke and Alexander Borodavka
DOI: 10.15252/embj.2021107711

Corresponding author: Alexander Borodavka (ab2677@cam.ac.uk)

Review Timeline:

Submission Date:	12th Jan 21
Editorial Decision:	18th Jan 21
Author Correspondence:	18th Jan 21
Editor Correspondence:	18th Jan 21
Revision Received:	3rd Mar 21
Editorial Decision:	28th Apr 21
Revision Received:	23rd Jun 21
Editorial Decision:	6th Aug 21
Revision Received:	23rd Aug 21
Accepted:	27th Aug 21

Editors: Ieva Gailite / Hartmut Vodermaier

Transaction Report:

(Note: With the exception of the correction of typographical or spelling errors that could be a source of ambiguity, letters and reports are not edited. Depending on transfer agreements, referee reports obtained elsewhere may or may not be included in this compilation. Referee reports are anonymous unless the Referee chooses to sign their reports.)

1st Editorial Decision 18th Jan 2021

Thank you for submitting your manuscript "Liquid-Liquid Phase Separation Underlies the Formation of Replication Factories in dsRNA Viruses" for consideration by The EMBO Journal. I apologise for the unusual delay in the assessment of your manuscript due to the high manuscript submission rate to our journal at the moment. I have now read your study carefully and discussed it with my colleagues. I am afraid that we have decided not to pursue the publication at The EMBO Journal, but I recommend a transfer to our sister journal EMBO Reports, where the responsible editor would be interested in sending the manuscript for peer review.

We appreciate that your study shows that rotavirus replication factories/viroplasms form liquid condensates via NSP2 and NSP5 interaction. Further findings show that these condensates undergo a shift to solid phase later in infection, and their disruption causes viral RNA dispersal, but does not induce RNA dissociation from NSP2. While we find the proposed role of liquid-liquid phase separation in viral genomic RNA fragment concentration for successful packaging into virions interesting, we also noted that the functional effect of liquid condensate disruption on viral replication efficiency remains to be shown. Thus, also noting that liquid-liquid phase separation has been implicated in viroplasm formation in various other viruses, we unfortunately concluded that the broader conceptual advance provided in your current manuscript in its current form is not sufficiently striking to offer peer review and publication in The EMBO Journal.

Author Correspondence **18th Jan 2021**

thank you very much for taking the time to read our manuscript - I am glad you've emailed me today, as we have had some very exciting results that I am sure you'd also find exciting! Since submission, we have recently finalised quantification of the effect of propylene glycol treatment on rotavirus replication. The results are very nice indeed, since we see the 10,000-fold reduction in the virus titre. We are now persuing testing other compounds that we identified in our screens that disrupt LLPS. Overall, these data are the first of its kind to demonstrate the disruption of LLPS with propylene glycol which is well-tolerated by cells, can lead to the reduction of virus replication. What's really fascinating about this system, is the fact that we can reconstitute NSP5/NSP2 condensates in vitro; ii) disrupt them in vitro and iii) in vivo using matching PG or hexane diol concentrations.

I hope you will share our excitement, and you'd be able to reconsider sending out the revised version of the manuscript for further review in your journal.

Editor Correspondence **18th Jan 2021**

These certainly sound like very interesting results, and I would be happy to

reconsider the manuscript if this information could be added.

1st Revision - Editorial Decision 28th Apr 2021

Thank you for submitting your manuscript for consideration by the EMBO Journal. We have now received two referee reports on your manuscript, which are included below for your information.

As you will see from the comments, both reviewers with expertise in reovirus replication find the topic of interest. However, while reviewer #1 is more positive in their assessment, reviewer #2 points out issues with the characterisation of the proposed liquid droplet properties of rotavirus replication factories and asks for better integration of the results with the previous analyses on the role of NSP2/NSP5 phosphorylation and viroplasm association with lipid droplets for rotavirus replication. Furthermore, both reviewers ask for clarification of the comparative role of NSP2 and NSP5 in condensate formation.

Unfortunately, reviewer #3 with expertise in liquid-liquid phase separation was not able to submit their report. I have contacted an alternative advisor on this aspect but have not received their input so far. Since I have not received their comments in a reasonable timeframe, I will ask for such input on the revised study, which will also be taken into account in the decision process. Therefore, to avoid further delays in the decision process and based on the two available reports, I would like to invite you to address the comments of the referees in a revised version of your manuscript.

Please also note that the acceptance of the manuscript will depend on the reviewer assessment of the revised study. I think it would also be helpful to discuss the revision in more detail via email or phone/videoconferencing - please let me know what time would be convenient for you.

Referee #1:

In this paper, Geiger et al report major new findings about the formation of rotavirus replication complexes, which are called viroplasms. The authors discovered that rotavirus nonstructural proteins NSP2 and NSP5 can phase-separate when mixed, establishing for the first time a convincing mechanism that describes how rotavirus viroplasms are built. These findings advance an understanding of dsRNA virus replication and yield another conclusive example of the natural utility of phase-separating condensates. While precise criteria have not been established for the conclusive demonstration of condensates, this group did an admirable job of gathering evidence to support their claims. The following data strongly support the main conclusions of the study:

1. NSP5-eGFP-labeled viroplasms are dynamic structures that appear and act like condensates, exhibiting typical FRAP recovery curves as seen with condensates formed in other systems.

2. Treatment of infected cells with 1,6 hexanediol (and other aliphatic diols) reversibly disrupts viroplasms, and longer treatment of infected cells with PG impedes rotavirus replication.

3. Using a clever microfluidic device, phase diagrams of purified NSP2 and NSP5 were obtained to establish conditions for condensate formation in vitro.

Other important findings are that the dynamics of viroplasms decrease with size and age, and RNA is not required to nucleate early condensates. The authors were able to nicely tie together these findings with many previously published results to develop a model to explains the formation of rotavirus viroplasms. This is an important study.

Some minor questions and comments that might enhance the presentation of the findings are as follows:

1. What are the possible explanations for a decrease in viroplasm dynamics, and how might these changes affect the processes that occurs within these structures?

2. In Figure 4A, NSP2 and NSP5 appear to have high LLPS scores, but they do not phase-separate unless mixed together. Are the authors aware of a minimal average LLPS score that more accurately predicts whether a protein is capable of phase-separation on its own?

3. Since isolated NSP5 does not phase-separate and appears to require NSP2, shouldn't both proteins be referred to as scaffolds?

4. RNA should be mixed with NSP2 and NSP5 in vitro to obtain phase diagrams in order to determine whether condensate formation is positively affected.

5. How well does rotavirus infection recover after reversal of PG treatment as shown in Figure 5A? It appears that there are many more Seg3 RNA granules in the cytoplasm. Will these RNA granules and viroplasms ever coalesce? While the viroplasm structures reform, it is possible that viroplasm functionality does not. Such experiments could define potential therapeutic doses.

Referee #2:

Review: The EMBO Journal [EMBOJ-2021-107711R-Q]
Liquid-liquid Phase Separation Underpins the Formation of Replication Factories in Segmented dsRNA Viruses
Authors: Florian Geiger, Guido Papa, William E Arter, Julia Acker, Kadi L Saar, Nadia A Erkamp, Runzhang Qi, Jack PK Bravo, Sebastian Strauss, Georg Krainer, Oscar R. Burrone, Ralf Jungmann, Tuomas P J Knowles, Hanna Engelke, and Dr. Alexander Borodavka

Evidence is mounting that liquid-liquid phase separation (LLPS) form membrane-less compartments in cells. Many viruses form cellular compartments called viral factories or in the case of rotavirus, viroplasms, required for virus replication. However, the mechanism for the formation of viroplasms remains largely unknown. The rotavirus nonstructural proteins NSP2 and NSP5 are required for the formation of viroplasms. Geiger et al., suggest NSP5 and NSP2 undergo liquid-liquid phase separation to form viroplasms. They used several techniques to provide evidence of LLPS: 1)

cytosolic inclusions formed by NSP5/NSP2 are initially spherical; 2) two or more NSP5-rich droplets can fuse and relax into a sphere; 3) infection with the NSP5-defficient rotavirus mutant does not yield these droplets, unless NSP5 is produced in trans; 4) these droplets are formed upon NSP5 and NSP2 mixing in vitro, and when these proteins are co-expressed in vivo; and 5) NSP2/NSP5 droplets can be instantly dissolved when treated with aliphatic alcohols known to disrupt multivalent interactions driving liquid-liquid phase separation. At later times post infection, viroplasms did not dissolve in the presence of aliphatic diols suggesting a liquid-to-solid transition.

Although the authors used several techniques to provide evidence of LLPS, biological phase separation must be rigorously characterized. Several of the techniques used, FRAP and treatment with hexanediol should not be used as a definitive diagnostic for determining if LLPS is the mechanism of assembly of a structure (Alberti et al., Cell 2019). Hexanediol should be used with caution when used on live cells because it changes the permeability of membranes and thus can lead to additional artifacts (Kroschwald et al., Matters 2017).

Major considerations:

Intrinsically disordered domain(s) are associated with LLPS. The authors use a computer program to predict domains to promote LLPS (Fig. 4a black, and 4b) and compare their results with an EMBOSS Protein charge plot (Fig. 4a). Eichwald et al., J. Gen. Virol, 2004 showed that the N-terminal region (33 aa) as well as the C-terminal part (aa 131-198) of NSP5 are required for binding to NSP2 and essential for viroplasm formation (shown in green in Fig. 4a). Of the regions identified by the authors in Fig. 4b, only the residues around 150 overlap with the domains shown in Fig. 4a; however, the authors do not specify the amino acids involved so it is difficult to determine if this is the same regions reported by Eichwald et al. Mutation of the intrinsically disordered domains found by the authors and functional analysis of the residues in LLPS would increase the evidence that viroplasms are formed by LLPS. The fact that a given protein forms assemblies at high concentrations is not necessarily evidence that the phase separation ability of this protein is functionally relevant. To change the phase behavior it is necessary to introduce mutations to alter protein multivalency and prove that the assembly occurs by LLPS.

How do the authors reconcile their results with the results shown by Cheung et al., JV 2010 and Kim et al., JV 2011 that disruption of lipid droplet formation inhibits viroplasm formation? Lipid droplets also fuse and form spherical organelles in the cytoplasm. Although the authors state on page 19 "association of lipid droplets ... with viroplasms is thus entirely consistent with our model of formation of viral replicative factories" it is unclear how this occurs. Is the lipid droplet part of the LLPS? In addition, Criglar et al., JV 2020 used a recombinant virus expressing NSP2 mutated at one amino acid S313D to form a phosphomimic and showed that viroplasm assembly correlates with NSP5 hyperphosphorylation (also shown by many others) and (ii) NSP2 S313D colocalizes with rotavirus-induced lipid droplets without NSP5, suggesting that vNSP2 phospho-S313 is sufficient for interacting with LDs and may be the virus factor required for rotavirus-induced lipid droplet formation prior to viroplasm assembly. The authors address NSP5 phosphorylation but how does their model address these and other papers that demonstrate phosphorylation is required for viroplasm formation?

On page 17, the authors state, "we propose that rotavirus NSP5 acts as the primary scaffold required to form these condensates." Knocking out NSP2 also abolishes viroplasm formation and expression of NSP5 alone does not induce VLS. NSP2 also binds RNA. Why do the authors think that NSP5 acts as the primary scaffold and not NSP2?

The title including "in Segmented dsRNA Viruses" is overstated. It would appear from the title that all segmented dsRNA viruses assemble replication factories by LLPS. However, the authors only assessed rotavirus.

Why did most viroplasms not bleach at 12 hpi in Fig. 2a? Couldn't this also mean that less protein is going to the viroplasm?

Fig. 7, VP3 is the capping enzyme. There is not a rotavirus cap-binding protein.

Minor comments:

The introduction has too many results from the paper and includes information more suited for the discussion. The introduction should include more information about what is known about liquid-liquid phase.

Fig. 5b and the discussion of this figure on page 5 is misleading. The authors describe "Electron microscopy analysis of the NSP5-EGFP cells infected with RVs revealed multiple electron-dense granules (Fig. 1b), with the RNA-containing particles budding from their surfaces, further confirming that the observed cytoplasmic granules represent viral replication factories." From this description, it appears that particles assembly and bud directly from the viroplasms. Lopez et al., JVI, 2005 reported that silencing NSP4 resulted in the assembly of little or no viral particles indicating that the NSP4-containing membranes are necessary for the budding of nascent viral particles from viroplasms. The section omits the description of the particles budding through ER-COPII-derived NSP4-containing membranes described by Crawford et al., JV 2020. In addition, determining whether a particle is empty or filled with RNA can not be determined by thin section EM. Detection of the electron dense RNA depends on where the particle is sectioned.

Point-by-point responses

Referee #1:

In this paper, Geiger et al report major new findings about the formation of rotavirus replication complexes, which are called viroplasms. The authors discovered that rotavirus nonstructural proteins NSP2 and NSP5 can phase-separate when mixed, establishing for the first time a convincing mechanism that describes how rotavirus viroplasms are built. These findings advance an understanding of dsRNA virus replication and yield another conclusive example of the natural utility of phase-separating condensates. While precise criteria have not been established for the conclusive demonstration of condensates, this group did an admirable job of gathering evidence to support their claims. The following data strongly support the main conclusions of the study:

1. NSP5-eGFP-labeled viroplasms are dynamic structures that appear and act like condensates, exhibiting typical FRAP recovery curves as seen with condensates formed in other systems.

2. Treatment of infected cells with 1,6 hexanediol (and other aliphatic diols) reversibly disrupts viroplasms, and longer treatment of infected cells with PG impedes rotavirus replication.

3. Using a clever microfluidic device, phase diagrams of purified NSP2 and NSP5 were obtained to establish conditions for condensate formation in vitro.

Other important findings are that the dynamics of viroplasms decrease with size and age, and RNA is not required to nucleate early condensates. The authors were able to nicely tie together these findings with many previously published results to develop a model to explains the formation of rotavirus viroplasms. This is an important study.

We thank the expert reviewer for their very positive comments, below are our further responses.

Some minor questions and comments that might enhance the presentation of the findings are as follows:

1. What are the possible explanations for a decrease in viroplasm dynamics, and how might these changes affect the processes that occurs within these structures?

This is an interesting and a very complex question. Maturation of condensates is a well-known phenomenon that results in changes of their material properties (e.g., liquid-like to gel-like), that would explain a decrease in viroplasmic dynamics. Along the condensation pathway, proteins undergoing LLPS may convert from their native state to non-native states (e.g., amyloid-like). These states are metastable, and the functional droplet states are commonly stabilised by extrinsic factors, e.g., RNA or post-translational modifications (PTMs). The reported observations of decreased viroplasm dynamics at later stages of infection appear to coincide with both extensive phosphorylation of NSP5 ('NSP5 hyperphosphorylation') and higher RNA content. Thus, we hypothesize that phosphorylation

may play role in maturation of the NSP5/NSP2 condensates (e.g., due to modulation of the electrostatic interactions between NSP5 and its clients). Another important aspect of viroplasm maturation is the evolution of the viroplasmic protein:RNA composition throughout the infection. As a multivalent polyanion, RNA plays an important role in maintaining the composition and the liquid-like state of many condensates. Indeed, late stage viroplasms (i.e., > 6 HPI) have been reported to associate with lipid droplets [1], *ER and microtubules* [3]. *However, these cellular components have been reported to directly partition into the NSP5/NSP2 condensates. We hope that our studies will lay the foundation for investigating physicochemical properties of viroplasms at distinct stages of their formation in order to elucidate how these condensates dynamically change and interact with other cellular components.*

2. In Figure 4A, NSP2 and NSP5 appear to have high LLPS scores, but they do not phase-separate unless mixed together. Are the authors aware of a minimal average LLPS score that more accurately predicts whether a protein is capable of phase-separation on its own?

We thank the reviewer for this comment – we have now clarified the minimum DeePhase Score expected for residues that drive LLPS (> 0.5). We have added the following statement:

'To dissect the sequence features of NSP5 that drive its phase-separation, we employed our recently developed machine learning approach termed DeePhase[4] *to identify the LLPS-prone regions. The overall DeePhase score of 0.61 indicated that NSP5 meets the criteria of a phase-separating protein, i.e., DeePhase score of > 0.5. In contrast, global DeePhase score of NSP2 of 0.2 suggested that this RNA chaperone has low propensity to drive phase-separation. Further sequence analysis of NSP5 with a moving average of 30 amino acid residues revealed several regions with high propensity to drive phase separation, i.e., LLPS score > 0.5 (**Fig. 5a**). Remarkably, these LLPS-prone regions overlapped with the two sections of NSP5 previously shown to be crucial for viroplasm-like structure assembly with NSP2*[5] *(**Fig. 5a**, regions highlighted in green)'.*

We would like to note that currently there is no single accurate predictor of LLPS due to the complexity of interactions involved in the formation of these condensates. We (Kadi L Saar et al 2021)[4] *have recently published an in silico strategy for understanding the associations between protein sequence and phase behavior and further constructed machine-learning models for predicting LLPS. This analysis highlighted that on average, LLPS-prone proteins are more disordered and less hydrophobic, showing a fine balance in their relative content of polar and hydrophobic residues. Unlike NSP2 and other early infection viroplasmic proteins (VP1, VP2 and potentially VP3), NSP5 meets all the criteria of such proteins, i.e., possessing multiple IDRs, low complexity sequences, and thus having a higher DeePhase Score of 0.61 (vs 0.2 for an RNA-binding, stable octamer NSP2).*

3. Since isolated NSP5 does not phase-separate and appears to require NSP2, shouldn't both proteins be referred to as scaffolds?

Thank you for this question. We have now added new data demonstrating that NSP5 is capable of forming condensates on its own, particularly in the presence of crowding agents (PEG 20K), or when mixed with charged homopolymers, whereas NSP2 does not (Fig. 4a). This is reflected in the revised Figure 4. Moreover, we have also included new data, as shown in Fig. 5, revealing the role of NSP5 oligomerization in LLPS by generating a non-

oligomerising mutant of NSP5 that lacks the last 18 C-terminal residues. We have made a new Figure 5 to emphasize the role of NSP5 as a scaffold that drives LLPS. Classically, proteins capable of forming droplets on their own (NSP5) are referred as 'scaffolds', whereas other macromolecular 'clients' do not[6,7]*. Moreover, since NSP2 is a well-characterised RNA chaperone, we have previously extensively studied its behavior in the presence of RNAs of different lengths. We hypothesized that a multivalent, RNA-binding protein such as NSP2, could, in principle, form condensates with RNAs. However, unlike NSP5, which readily formed NSP5/poly-arginine or poly-lysine droplets, NSP2 did not form condensates with RNAs under our experimental conditions, as we have previously demonstrated by investigating various NSP2:RNA complexes using fluorescence correlation spectroscopy* [8]*. Lastly, only co-expression of NSP5 with either NSP2, or the inner capsid protein VP2, leads to the formation of viroplasm-like inclusions in cells* [9,10]*. In each case, NSP5 is always the required for viroplasm-like structure (VLS) formation, and its C-terminal truncation fails to form VLS, further suggesting that NSP5 should be regarded as a scaffold, and NSP2, VP2 and VP1 (based on its high nM affinity for NSP5*[11]*) being its clients. Perhaps, a more appropriate term to describe NSP5 would be 'a driver' of phase-separation* [7]

4. RNA should be mixed with NSP2 and NSP5 in vitro to obtain phase diagrams in order to determine whether condensate formation is positively affected.

We thank the reviewer for this comment. Mapping three component (RNA:NSP5:NSP2) phase diagrams would indeed be the next logical step. However, such experiments will constitute a new study, since mapping phase diagrams for multi-component biomolecular condensates, and their in-depth characterisation remains poses many experimental challenges. Both length and structure of the RNA chosen will likely affect the phase diagram[12–14]*. In addition, other RNA-binding proteins, e.g., (RNA-dependent RNA Polymerase VP1) are likely to be involved in the RNA partitioning into these condensates. Work on the roles of these clients is currently under way.*

5. How well does rotavirus infection recover after reversal of PG treatment as shown in Figure 5A? It appears that there are many more Seg3 RNA granules in the cytoplasm. Will these RNA granules and viroplasms ever coalesce? While the viroplasm structures reform, it is possible that viroplasm functionality does not. Such experiments could define potential therapeutic doses.

We have carried out additional studies of the effects that PG exerts on (i) viroplasms, and (ii) viral replication. These results are now summarized in the updated Figure 6. A brief 15 min treatment with 4.7% PG does not significantly alter viral titres measured approximately 6 h after PG application. In contrast, prolonged (5 h) PG treatments would significantly decrease viral titres, in accord with the observed sensitivity of viroplasms to towards PG. With respect to the RNA coalescence - while we can track the recovery of fluorescently labelled NSP5 in live cells, unfortunately, live cell imaging and tracking of the RNA component of condensates, particularly, in viruses, remains a very challenging, unattainable task. We therefore focused on imaging of the RNA component using a more conventional smFISH approach. However, our approach required cell fixation, and as such we were unable to directly track the recovery and coalescence of viral RNAs.

Referee #2:

Evidence is mounting that liquid-liquid phase separation (LLPS) form membrane-less compartments in cells. Many viruses form cellular compartments called viral factories or in the case of rotavirus, viroplasms, required for virus replication. However, the mechanism for the formation of viroplasms remains largely unknown. The rotavirus nonstructural proteins NSP2 and NSP5 are required for the formation of viroplasms. Geiger et al., suggest NSP5 and NSP2 undergo liquid-liquid phase separation to form viroplasms. They used several techniques to provide evidence of LLPS: 1) cytosolic inclusions formed by NSP5/NSP2 are initially spherical; 2) two or more NSP5-rich droplets can fuse and relax into a sphere; 3) infection with the NSP5-defficient rotavirus mutant does not yield these droplets, unless NSP5 is produced in trans; 4) these droplets are formed upon NSP5 and NSP2 mixing in vitro, and when these proteins are co-expressed in vivo; and 5) NSP2/NSP5 droplets can be instantly dissolved when treated with aliphatic alcohols known to disrupt multivalent interactions driving liquid-liquid phase separation. At later times post infection, viroplasms did not dissolve in the presence of aliphatic diols suggesting a liquid-to-solid transition.

Although the authors used several techniques to provide evidence of LLPS, biological phase separation must be rigorously characterized. Several of the techniques used, FRAP and treatment with hexanediol should not be used as a definitive diagnostic for determining if LLPS is the mechanism of assembly of a structure (Alberti et al., Cell 2019). Hexanediol should be used with caution when used on live cells because it changes the permeability of membranes and thus can lead to additional artifacts (Kroschwald et al., Matters 2017).

We thank the reviewer for their comments, and particularly for pointing out this wonderful review by Alberti et al, which we have referenced in our publication. Indeed, Alberti et al., (Cell 2019), name the following criteria for defining a phase-separated structure: they are spherical, they fuse and recover after photobleaching. We have demonstrated that our system meets all these criteria. Although this critical review does mention the caveats of FRAP assessment of condensates (e.g., full-FRAP reporting not only on the exchange rate between the dilute and dense phase), the authors also state that 'it can be still useful for assessing extremes in material state or changes in the material state through time' (sic). Because of these challenges, we did not estimate NSP5-EGFP diffusion rates from the observed FRAP rates; however, we show clear differences between the two extremes of the two states of viroplasms that evolve throughout the infection (i.e., sensitive to aliphatic diols, spherical droplets with high recovery rates during early infection vs. insensitive to 1,6-HD more anisotropic inclusions with slow recovery rates). More importantly, these two types of viroplasms can be found within the same cell (Figure 3a), notably the second type of viroplasms observed only after 6 HPI. Even if such brief treatments with 1,6-HD did change the permeability of membranes, this could not explain the differences in the solubility of such condensates within the same cell. Removal of 1,6-HD from the cell culture medium also resulted in almost immediate recovery of these condensates, as we have demonstrated throughout the manuscript (Supplementary Movies 1-3). Again, these results cannot be explained by changes in membrane permeability. Finally, we have also used 1,6-HD in vitro, thus demonstrating that same concentration of 1,6HD is required for inhibiting NSP5/NSP2 condensation in vitro.

Major considerations:

Intrinsically disordered domain(s) are associated with LLPS. The authors use a computer program to predict domains to promote LLPS (Fig. 4a black, and 4b) and compare their results with an EMBOSS Protein charge plot (Fig. 4a). Eichwald et al., J. Gen. Virol, 2004 showed that the N-terminal region (33 aa) as well as the C-terminal part (aa 131-198) of NSP5 are required for binding to NSP2 and essential for viroplasm formation (shown in green in Fig. 4a). Of the regions identified by the authors in Fig. 4b, only the residues around 150 overlap with the domains shown in Fig. 4a; however, the authors do not specify the amino acids involved so it is difficult to determine if this is the same regions reported by Eichwald et al.

We thank the reviewer for these comments. Since our original submission, our DeePhase approach has been formally peer-reviewed and published (Kadi L Saar et al., 2021, PNAS). We have used an updated version of the algorithm to re-evaluate the global LLPS scores for both NSP5 and NSP2. We have removed the DeePhase plot for NSP2 to avoid any confusion, as the overall LLPS score of NSP2 (0.2) is below of that required for driving LLPS (Global DeePhase score of 0.5). In contrast, the overall DeePhase score of NSP5 is > 0.6, suggesting that NSP5 is likely to undergo LLPS on its own. Regarding specific residues, we would like to point out that as DeePhase calculates a moving average of the LLPS score, we only highlighted regions containing residues with high propensity to drive LLPS. Since DeePhase recognised global features of the LLPS drivers (i.e., LLPS-prone proteins are more disordered and less hydrophobic, showing a fine balance in their relative content of polar and hydrophobic residues), it is most sensitive when used to analyse a complete protein sequence (or a specific domain/larger region containing multiple residues). We therefore used a larger sliding window (30 residues) to improve the sensitivity of the approach to detect LLPS-prone sequences of NSP5, as shown in the new Fig.5. Using the sliding window approach, however, does not allow precise identification of the individual residues that drive LLPS. Furthermore, unlike global DeePhase score that has higher positive predictive value for LLPS behaviour, analyses of individual residues, or of their average, may result in certain biases, i.e., higher relative LLPS scores. Therefore, we started with estimating the global scores for NSP5 (0.61) and NSP2 (0.2), which was followed by the 30-residue analysis of NSP5.

Secondly, we would like to point out that Eichwald et al. publication[5] focuses on the regions of NSP5 that were important for viroplasm-like structure formation, however, this study did not identify NSP2-binding residues. In fact, a later publication, by Jiang et al., revealed that the N-terminal region of NSP5 is dispensable for the interaction between NSP5 and NSP2, since the absence of NSP5 residues 1–65 does not prevent formation of the complex[15]. In contrast, Martin et al 2011 [16] have proposed that the polybasic NSP5 region 132–146 interacts with NSP2, while the far-Western experiments suggested that the C-terminal residues of NSP5 were not required for NSP2 binding. The authors, however, show that when 'the C-terminal residues of NSP5 were removed, it no longer co-localised with NSP2 and no VLSs were formed' (sic), completely in accord with the results of our in vitro LLPS assay presented in the new Figure 5. In summary, our data is in fact in agreement with the latest results published by Martin et al (2011).

Mutation of the intrinsically disordered domains found by the authors and functional analysis of the residues in LLPS would increase the evidence that viroplasms are formed by LLPS. The fact that a given protein forms assemblies at high concentrations is not necessarily

evidence that the phase separation ability of this protein is functionally relevant. To change the phase behavior it is necessary to introduce mutations to alter protein multivalency and prove that the assembly occurs by LLPS.

We would like to thank the reviewer for the excellent suggestion to test this idea. Indeed, Martin et al, 2011 [16] have convincingly demonstrated that the C-terminal residues of NSP5 are required for NSP5 oligomerisation, and were shown to be crucial for VLS formation when co-expressed with NSP2. We therefore expressed and purified ΔC-NSP5 lacking the last 18 residues predicted to contribute to phase separation (high DeePhase Score, Figure 5a). Removal of these residues does not alter the overall negatively charged nature of NSP5 (Figure 5b). These new results are now summarized in Figure 5 as follows: (i) non-oligomerizing NSP5 mutant does not form protein droplets with NSP2; (ii) importantly, this mutant has also lost its capacity to make condensates with poly-arginine (Figure 5c) and PEG-20K, directly demonstrating that this oligomerisation region is indeed crucial for the LLPS of NSP5. Assuming the results published by Jiang et al (2006) and Martin et al (2011) are correct, and that the C-terminal residues of NSP5 are not involved in NSP2 binding, our new data reveal that NSP2 binding to NSP5 alone is not sufficient to drive LLPS of the system, and demonstrate the key role of NSP5 oligomerization in its phase-separation behaviour. Lastly, we show that despite its loss of capacity to undergo LLPS, this mutant could still partition into the condensates formed by full-length NSP5 (Figure 5c). These data demonstrate that ΔC-NSP5 mutant is able to engage in either homotypic (NSP5) protein-protein interactions, or more likely heterotypic (with NSP2) interactions within these condensates. We have also included a reference to our publication demonstrating the crucial role of the CTR of NSP5 for rotavirus replication and viroplasm formation (Papa et al., J Virol., 2020) [17].

Secondly, we have also addressed the issue around NSP5 concentration during RV infection. These new results have been added in the new Figure 4c. We have estimated NSP5 concentration in the cytoplasm of RV infected cells between 2-6 hours post infection. Our estimates suggest that intracellular [NSP5] ~ 0.3 – 10 μM, which is indeed lower than some protein concentrations reported for other systems known to undergo LLPS in vitro. This is not the case for NSP5:NSP2 condensates, as seen from our in vitro LLPS experiments (Figure 4b) that reveal phase-separation occurring at low μM protein concentrations. Importantly, we report a clear shift in the phase boundary that results in complete solubilization (no condensates formed) at 4% (v/v) 1,6-HD concentrations in vitro. These quantitative results are entirely consistent with our in vivo data, further confirming that the observed effect of 1.6-HD on viroplasms has nothing to do with cellular response to this compound.

How do the authors reconcile their results with the results shown by Cheung et al., JV 2010 and Kim et al., JV 2011 that disruption of lipid droplet formation inhibits viroplasm formation? Lipid droplets also fuse and form spherical organelles in the cytoplasm. Although the authors state on page 19 "association of lipid droplets ... with viroplasms is thus entirely consistent with our model of formation of viral replicative factories" it is unclear how this occurs. Is the lipid droplet part of the LLPS?

We have clarified this statement in our discussion. The data by Cheung et al, JVI 2010 indeed demonstrate that viroplasm formation ***precedes*** *their interaction with lipid droplets (LDs), with which they interact only from 5-6 HPI. Thus, formation of NSP5/NSP2 condensates is unlikely to have been affected by recruitment of LDs. It can be also argued that association of*

LDs with viroplasms at later stages of infection (ca 8 HPI, as reported in Cheung et al., JVI 2010) could be related to the maturation of the NSP5/NSP2 condensates, as discussed above. This is a very interesting point, but dissecting the exact molecular mechanism of condensate maturation throughout the infection would extend beyond the scope of this study.

In addition, Criglar et al., JV 2020 used a recombinant virus expressing NSP2 mutated at one amino acid S313D to form a phosphomimic and showed that viroplasm assembly correlates with NSP5 hyperphosphorylation (also shown by many others) and (ii) NSP2 S313D colocalizes with rotavirus-induced lipid droplets without NSP5, suggesting that vNSP2 phospho-S313 is sufficient for interacting with LDs and may be the virus factor required for rotavirus-induced lipid droplet formation prior to viroplasm assembly. The authors address NSP5 phosphorylation but how does their model address these and other papers that demonstrate phosphorylation is required for viroplasm formation?

Regarding the LD point raised by the reviewer – we have already commented on the observations of lipid droplets etc observed during later infection stages. In fact, Criglar et al, 2020 study states 'time course of viroplasm formation and interaction with LDs suggests that viroplasms form early (2 to 4 hours postinfection [hpi]) before recruiting or interacting with LDs' (sic). In contrast, our study only discusses the mechanism of early viroplasm nucleation through LLPS of NSP5 and NSP2. We would like to point out that although multiple studies demonstrated hyperphosphorylation of NSP5 during viroplasm assembly, it does not mean that NSP5 phosphorylation is a prerequisite of NSP5/NSP2 condensation. In fact, our in vitro data demonstrate that NSP5/NSP2 condensation spontaneously occurs in vitro, and the establishment of such a reconstituted system from purified NSP5 and NSP2 would be an highly valuable tool for interrogating maturation of these condensates and roles of PTMs in this process. These will include dissection of each step of NSP5 phosphorylation, investigation of NSP5 dynamics that leads to its phosphorylation, and its role in condensate/viroplasm maturation.

Importantly, the most recent comprehensive study of NSP5 phosphorylation that has now somewhat superseded previous results describing NSP5 phosphorylation was carried out using a fully tractable reverse genetics system for rotaviruses[17]. We summarise these observations as follows:

(i) *Using S67A NSP5 mutant, the study unambiguously demonstrates that phosphorylation of NSP5 is important, but not essential for viroplasm formation during early stage (~ 5-10 HPI) of infection. Albeit smaller in sizes, S67A viroplasms contained NSP5/NSP2, and viral RNA, while S67A NSP5 was not phosphorylated. Intriguingly, the titre of S67A virus deficient in NSP5 phosphorylation was about 100-fold lower compared to the WT virus. This reduction is comparable to the observed drop in the titre after 4.7% PG treatments of cells during early infection window that also resulted in the reduction of NSP5 phosphorylation. Thus, the observed lack of phosphorylation does not abrogate NSP5/NSP2 binding during RV infection.*

(ii) *Intriguingly, however, during late infection stages (10-12 HPI), S67A mutant formed large, irregular rod-like inclusions in cells[17]. These results are in accord with the observed rod-like aggregation of NSP5 during late RV infection when casein kinase1a (CK1a) was inhibited, thus impairing NSP5 phosphorylation[18].*

These aberrant NSP5 structures did not contain viral RNA, suggesting they are non-functional condensates of NSP5 that did not support RV replication[19].

(iii) *Removal of the last 18 AA (as in our ΔC-NSP5 mutant shown in Figure 5) resulted in complete loss of viroplasm assembly, viral replication, and NSP5 phosphorylation, confirming that unlike phosphorylation, NSP5 oligomerisation is absolutely required for condensate formation and for NSP5 phosphorylation.*

Given that these data demonstrate the importance of the oligomerization region in condensate formation, our revised model proposes that NSP5/NSP2 condensation occurs first, followed by NSP5 phosphorylation/hyperphosphorylation. We propose that phosphorylation is a consequence of NSP5/NSP2 condensation, and it is required for regulation of the maturation of NSP5 condensates during infection, when large amounts of condensate-forming proteins are rapidly accumulating in cells during infection. Dissolution of NSP5/NSP2-rich condensates in the presence of 4.7% PG demonstrate reduction of phosphorylation of NSP5, which could be reversed by inhibiting cellular phosphatases with okadaic acid (Figure 6). In fact, this model has been proposed in the past, suggesting that 'soluble NSP5 is constitutively dephosphorylated by cellular phosphatases'[20].

To our knowledge, these results represent the only reported example of modulation of LLPS during viral replication, which resulted in (i) alteration of the phosphorylation state of the scaffold protein (NSP5) and (ii) reduction of viral replication. Loss of phosphorylation could be blocked in vivo by inhibiting cytoplasmic phosphatases; and PG treatments at concentrations required for condensate solubilization caused reduction in virus production only when such treatments were applied during early stages of RV infection (1-5 HPI), but not late stages (5-10 HPI), when viroplasms become refractory to PG treatments.

In summary, we propose that viroplasms present a remarkable example of complex condensates that evolve during the RV infection in response to rapid changes in the cytoplasmic NSP5, NSP2 and RNA concentrations during the early stages of infection. Posttranslational modifications, particularly phosphorylation of resides within IDRs, are highly abundant and well-documented in literature, and are well-known to play crucial roles in maintaining liquid-like states or controlling maturation of condensates.

On page 17, the authors state, "we propose that rotavirus NSP5 acts as the primary scaffold required to form these condensates." Knocking out NSP2 also abolishes viroplasm formation and expression of NSP5 alone does not induce VLS. NSP2 also binds RNA. Why do the authors think that NSP5 acts as the primary scaffold and not NSP2?

We thank the reviewer for this comment. Please see our response to a similar question raised by the Reviewer 1.

The title including "in Segmented dsRNA Viruses" is overstated. It would appear from the title that all segmented dsRNA viruses assemble replication factories by LLPS. However, the authors only assessed rotavirus.

Agreed, we have changed the title to 'in Rotaviruses'. We do, however, believe that similar mechanism is exploited by other related dsRNA viruses, and several pieces of unpublished data strongly suggest this might be correct.

Why did most viroplasms not bleach at 12 hpi in Fig. 2a? Couldn't this also mean that less protein is going to the viroplasm?

Full FRAP reports on the exchange rate of NSP5-EGFP between the dilute and dense phase. The amount of NSP5-EGFP produced remains constant between 2-6 HPI (Supplementary figure 4f). As the reviewer suggests, our interpretation is exactly what they say – less NSP5-EGFP is exchanged between the viroplasm (dense phase) and the cytoplasmic (dilute) phase, due to the change of material properties of viroplasms. This is further corroborated by their overall decreased mobility, loss of sphericity, and being refractory to 1,6-HD or PG treatments.

Fig. 7, VP3 is the capping enzyme. There is not a rotavirus cap-binding protein.

Agreed, this has now been fixed.

Minor comments:

The introduction has too many results from the paper and includes information more suited for the discussion. The introduction should include more information about what is known about liquid-liquid phase.

We have expanded the Intro section and included a whole new paragraph to summarise the current knowledge of LLPS.

Fig. 5b and the discussion of this figure on page 5 is misleading. The authors describe "Electron microscopy analysis of the NSP5-EGFP cells infected with RVs revealed multiple electron-dense granules (Fig. 1b), with the RNA-containing particles budding from their surfaces, further confirming that the observed cytoplasmic granules represent viral replication factories." From this description, it appears that particles assembly and bud directly from the viroplasms. Lopez et al., JVI, 2005 reported that silencing NSP4 resulted in the assembly of little or no viral particles indicating that the NSP4-containing membranes are necessary for the budding of nascent viral particles from viroplasms. The section omits the description of the particles budding through ER-COPII-derived NSP4-containing membranes described by Crawford et al., JV 2020. In addition, determining whether a particle is empty or filled with RNA can not be determined by thin section EM. Detection of the electron dense RNA depends on where the particle is sectioned.

We have changed these figures and figure legends. We changed 'particles' to double-layered particles (DLPs), we also replaced 'budding' to 'emerge' from the surface. As suggested by the reviewer, Lopez et al observed budding of nascent particles, which indeed requires NSP4. In our image we only show DLPs. We did not discuss the subject of virus budding through ER-COPII-derived NSP4-containing membranes, as this is irrelevant to the scope of our study. Yet, we felt that showing an EM image of a viroplasm would be very useful for non-rotavirus experts, who are interested in LLPS and condensates.

References:

1. Cheung, W. *et al.* Rotaviruses associate with cellular lipid droplet components to replicate in viroplasms, and compounds disrupting or blocking lipid droplets inhibit viroplasm formation and viral replication. *J. Virol.* **84**, 6782–6798 (2010).
2. Crawford, S. E. & Desselberger, U. Lipid droplets form complexes with viroplasms and are crucial for rotavirus replication. *Curr. Opin. Virol.* **19**, 11–15 (2016).
3. Eichwald, C. *et al.* Rotavirus viroplasm fusion and perinuclear localization are dynamic processes requiring stabilized microtubules. *PLoS One* **7**, 1–16 (2012).
4. Saar, K. L. *et al.* Learning the molecular grammar of protein condensates from sequence determinants and embeddings. *Proc. Natl. Acad. Sci. U. S. A.* **118**, (2021).
5. Eichwald, C., Rodriguez, J. F. & Burrone, O. R. Characterization of rotavirus NSP2/NSP5 interactions and the dynamics of viroplasm formation. *J. Gen. Virol.* **85**, 625–634 (2004).
6. Alberti, S., Gladfelter, A. & Mittag, T. Considerations and challenges in studying liquid-liquid phase separation and biomolecular condensates. *Cell* **176**, 419–434 (2019).
7. Ghosh, A., Mazarakos, K. & Zhou, H. X. Three archetypical classes of macromolecular regulators of protein liquid-liquid phase separation. *Proc. Natl. Acad. Sci. U. S. A.* **116**, 19474–19483 (2019).
8. Borodavka, A., Dykeman, E. C., Schrimpf, W. & Lamb, D. C. Protein-mediated RNA folding governs sequence-specific interactions between rotavirus genome segments. *Elife* **6**, (2017).
9. Buttafuoco, A., Michaelsen, K. & Tobler, K. Conserved Rotavirus NSP5 and VP2 Domains Interact and Affect Viroplasm. *J. Virol.* **94**, 1–21 (2020).
10. Sen, A., Sen, N. & Mackow, E. R. The formation of viroplasm-like structures by the rotavirus NSP5 protein is calcium regulated and directed by a C-terminal helical domain. *J. Virol.* **81**, 11758–11767 (2007).
11. Arnoldi, F., Campagna, M., Eichwald, C., Desselberger, U. & Burrone, O. R. Interaction of Rotavirus Polymerase VP1 with Nonstructural Protein NSP5 Is Stronger than That with NSP2. *J. Virol.* **81**, 2128–2137 (2007).
12. Zhang, H. *et al.* RNA Controls PolyQ Protein Phase Transitions. *Mol. Cell* **60**, 220–230 (2015).
13. Roden, C. & Gladfelter, A. S. RNA contributions to the form and function of biomolecular condensates. *Nat. Rev. Mol. Cell Biol.* doi:10.1038/s41580-020-0264-6
14. Iserman, C. *et al.* Genomic RNA elements drive phase separation of the SARS-CoV-2 nucleocapsid. *Mol. Cell* **80**, 1078-1091.e6 (2020).
15. Jiang, X. *et al.* Cryoelectron Microscopy Structures of Rotavirus NSP2-NSP5 and NSP2-RNA Complexes: Implications for Genome Replication. *J. Virol.* **80**, 10829–10835 (2006).
16. Martin, D., Ouldali, M., Ménétrey, J. & Poncet, D. Structural organisation of the rotavirus nonstructural protein NSP5. *J. Mol. Biol.* **413**, 209–221 (2011).
17. Papa, G. *et al.* Recombinant rotaviruses rescued by reverse genetics reveal the role of NSP5 hyperphosphorylation in the assembly of viral factories. *J. Virol.* **94**, (2020).
18. Campagna, M. *et al.* Impaired hyperphosphorylation of rotavirus NSP5 in cells depleted of casein kinase 1α is associated with the formation of viroplasms with altered morphology and a moderate decrease in virus replication. *J. Gen. Virol.* **88**, 2800–2810 (2007).
19. Papa, G. *et al.* Recombinant rotaviruses rescued by reverse genetics reveal the role of NSP5 hyperphosphorylation in the assembly of viral factories. *J. Virol.* **94**, 1–23 (2019).
20. Sen, A., Agresti, D. & Mackow, E. R. Hyperphosphorylation of the rotavirus NSP5 protein is independent of serine 67 or NSP2 and the intrinsic insolubility of NSP5 is regulated by cellular phosphatases. *J. Virol.* **80**, 1807–1816 (2006).

1st Revision - Editorial Decision 6th Aug 2021

Thank you for submitting your revised manuscript to our editorial office. We have now heard back from the original referee 1, as well as from an additional referee particularly familiar with LLPS aspects. Given that both the virologist and the new referee found the study conceptually interesting as well as generally convincing, we shall be happy to publish it in The EMBO Journal, following a final round of minor revision to incorporate the (mainly presentational) requests of referee 4.

Referee #1:

The authors have responded satisfactorily to the concerns I raised in my initial review of this paper. The findings are significant, innovative, and justify the conclusions. This is an important study that should attract a wide readership.

Referee #4:

The authors present evidence that viroplasms formed by a rotavirus composed of the proteins Nsp5 and Nsp2 form by phase separation. The authors make a compelling case with in vitro and in cell work, showing reversible droplets and using microscopy to evaluate the importance of the proteins involved. They identify Nsp5 as the scaffold based on in vitro and in cell evidence for necessity. Interestingly, they find that both 1,6 hexanediol and propylene glycol reversibly disassemble these condensates, while high salt concentration disrupts their formation. Overall the

work is well performed and described. It is also a set of exciting results with broad implications. A number of major and minor areas should be addressed.

Abstract:
The sentence starting "Some aspects of the assembly" is too vague - what aspects?
The abstract mentions propylene glycol but not hexanediol - this is surprising.

Introduction:
"solely dependent on physical forces": membrane assembly is dependent on physical forces too. Rephrase

The first sentence of the LLPS introduction paragraph should mention that they are not membrane bound.

"are typically organized into complex, multilayered structures" - it is probably not true that condensates comprising multiple components are necessarily multilayered.

MAJOR - the introduction does not discuss the questions in the field at all. The introduction should be reworked to say something about Nsp5 and Nsp2 proposed role and then say what they may do. It should probably also introduce them as E/D/S rich and R rich, respectively, and disordered. This section of the introduction also seems like a list of results which is should not be as said by an earlier review.

Results
MAJOR - it is not clear to my untrained eye from the data presented that the EM image contains for sure the viroplasm. Shouldn't immunogold labeling be done to show this structure is enriched in Nsp5/2 or something else to show what it is. Further, what else are we looking at and are there membranes here? Is the cell membrane forming vesicles and that is what is peeling off into the shapes we see as double layered RNA containing particles? For a broad audience, more information is needed.

"As most typical properties of liquid" this sentence is not clear.

Figure 2c just shows an evaluation of sphericity but should show an image or at least he main text should have a reference to the figure 1 or 2 panels where round droplets are seen in cells. Time axis should be in hours.

MAJOR: it is not clear why the authors used 1,2 and 1,3 propane diol instead of other diols. For example, 2,5 hexanediol is thought to be less effective. Nevertheless, their observation of propylene glycol is very interesting. I do suggest staying consistent in the figure labeling it as "PG" instead of "1,2PD" because next to 1,6HD you imagine that the authors are showing pentanediol instead of propane diol.

"anisotropic" is used throughout but this means "(of an object or substance) having a physical property that has a different value when measured in different directions. A simple example is wood, which is stronger along the grain than across it." So the authors need a different word - irregularly shaped?

MAJOR: The authors show CD data to say that the protein is 40% disordered. The authors should show the residuals/fit-line for the CD data to see if these values are really well explained by the

model. Furthermore, they should show disorder prediction (DISOPRED) and secondary structure prediction (PSI-PRED) (both are available from PSI-PRED webserver) vs residue for this protein. Finally, they should show domain structure or predicted domain structure. I suggest updating their discussion of the domains and also the deltaCTD variant using something like the presentation in Perdikari et al EMBO J 2020 https://www.embopress.org/doi/full/10.15252/embj.2020106478 where they show the impact of domains on phase separation of SARS-CoV-2 nucleocapsid protein. The authors may also consider looking at alphafold to see if the structure of the structured regions can be predicted.

MAJOR: the authors don't say anything about how the radius of hydration measurement tells them anything about the structure of the protein. Also, isn't Nsp5 dimeric? How is this considered here?

The EV2a chromatogram should be labeled with the protein we are looking at as well as 280 and 260. The 260/280 ratio should be explicitly listed.

"lacks a pi cloud" - I think a better wording would be "lacks sp2 hybridized groups in its sidechain". Also the difference between lys and arginine sidechain is not going to be about cation-pi as both are cations. Better to say that pi interactions are enhanced by arginine.

MAJOR: the authors mention that they use Arg-9 at the same concenetraiton as polyArg but are they counting in mass/volume equivalents (mg/ml) or in micromolar? They should do the experiment at equivalent mg/ml only.

MAJOR: it is exciting to use the highthroughput method to generate the phase diagram, but the authors need to test the values at least at a single concentration of one component (or maybe in a "t" or "cross") to show what these values mean, especially as things at the edges of their data are "ragged" and look unclear. At the very least, the authors should make reference to how the results from this assay have been compared to bulk macro studies in another system.

In figure 6 the authors should show the different channels separately and then show the merge. Also, some measure of colocalization in the Nsp area would be useful.

"the observed effect" what effect?

The labeling in Fig 6e is hard to follow

MAJOR: The authors need to show that the higher bands are phosphorylated (for example by phosphatase treatment gel) or point to the specific literature showing that this pattern has been observed before for phosphor Nsp5

"less isotropic" see above - replace with "less round"

Discussion
The paragraph starting "coacervation of viral RNA chaperone" seems to go on about RNA-RNA interactions which is not probed at all in the paper and seems disconnected.

A mention in the discussion linking to nucleocapsid protein phase separation that has been examine for SARS-CoV-2 as well as the work on measles virus nucleo and phosphoprotein LLPS by Sigrid
Milles et al should be included. Are these related? Does Nsp5/2 recruit human proteins in the

condensates?

Methods
Why is sodium fluoride used for the CD? The authors should mention in the methods.

References
Some references I noticed were messed up
Conicella et al. and Krosschwald et al ("Simon A"?) have errors. Other references likely do too. I suggest the authors check their reference library or reference strategy.

Some additional comments from a student reviewer are below. Note, I find that the main idea that Nsp5 is the scaffold well supported, but I suggest looking at these detailed comments and possibly performing experiments suggested to clarify the matter.

I reviewed the respective aspects of the revised manuscript. 3 are my major comments regarding LLPS of the NSP5/NSP2 complex:
1. Is addition of PEG a diagnostic of a "scaffold protein"? In the first submission, isolated NSP5 (the so-called "scaffold") does not undergo LLPS without NSP2. In the revised manuscript, they show that 35 uM NSP5 + PEG-20K forms tiny droplets (Fig. 4A bottom panel). In contrast, 25 uM of NSP2 + PEG-20 K does not. Why didn't they test NSP2 and NSP5 at the same concentration (35 uM) in the presence of PEG-20K?
2. To further support the argument that NSP5 is the scaffold, they mixed 50 uM NSP5 with 5 uM poly-arginine (Fig. 4A middle panel). The droplets formed by arginine were partially dissolved by 1-6 hexanediol. In the supplementary information, they mixed 25 uM of NSP5 and 75 uM NSP5 with 5 uM poly-lysine. At 75 uM, there are some small droplets but they don't show data with 16-hexanediol in the case of lysine. Also, in the legend of Figure EV3 b left, they mention that the experiment was done at 35 uM NSP5 but in the figure and the main text they used 25 uM NSP5. Because NSP5 formed droplets with homopolymers, they argue that NSP5 is the scaffold.
3. In Figure 5c top, they deleted the CTR oligomerization domain and they show that 25 uM delta-CTR in the presence of PEG and poly-arginine doesn't undergo LLPS. 10 uM NSP5 mixed with 10 uM NSP2 triggers phase separation but in this case, 25 uM NSP5 delta-CTR + 10 uM NSP2 does not. In Figure 5c bottom, they mixed 10 uM NSP2 + 25 uM NSP5 + 5 uM NSP5 delta-CTR to show that the deletion construct partitions into preformed NSP5/NSP2 droplets. They interpret this result by stating that delta-CTR is capable of forming heterotypic (with NSP2) and homotypic interactions (with NSP5). Does this experiment provide enough evidence for a protein to be called a "scaffold"? Does stoichiometric excess of NSP5 favor homotypic interactions?
Minor comments:
• Figure EV3: NSP2/NSP5 phase separates in physiological NaCl concentration but they don't mention the exact NaCl mM concentration anywhere in the text.
• Figure 1: It would be helpful to have a domain description of NSP2 and NSP5. Also, in the introduction they don't include details about the sequence composition and the biophysical characteristics of these sequences (length, charge, % disorder etc).
• They have an interesting method for making phase diagrams using a microfluidic device. In Figure 4b, they show how hexanediol creates one phase versus two phases. Since propylene glycol is safer than hexanediol and efficient in dissolving condensates, it would be interesting to make a phase diagram in the presence of propylene glycol as well. Also the coloring in Figure EV4e (LLPS: blue, mixed:red) is not consistent with Figure 4b (LLPS: red, mixed: blue). I am not sure how Figure 4EV e presents more information compared to Figure 4b.

Point-by-point responses (Additional Reviewer 4)

The authors present evidence that viroplasms formed by a rotavirus composed of the proteins Nsp5 and Nsp2 form by phase separation. The authors make a compelling case with in vitro and in cell work, showing reversible droplets and using microscopy to evaluate the importance of the proteins involved. They identify Nsp5 as the scaffold based on in vitro and in cell evidence for necessity. Interestingly, they find that both 1,6 hexanediol and propylene glycol reversibly disassemble these condensates, while high salt concentration disrupts their formation. Overall the work is well performed and described. It is also a set of exciting results with broad implications. A number of major and minor areas should be addressed.

We thank the reviewer for these overall positive comments, and for sharing their excitement about our results with us.

Abstract:
The sentence starting "Some aspects of the assembly" is too vague - what aspects?
The abstract mentions propylene glycol but not hexanediol - this is surprising.

We have removed this sentence from the abstract. Some parallels between other cytoplasmic RNP granule assembly and viroplasms are mentioned in more detail in the Discussion. We felt that our findings that propylene glycol dissolves early infection viroplasms would be an interesting and important observation for the wider readership of EMBO Journal. We have now mentioned 1,6-hexanediol in the abstract, and we provided the rationale behind testing propylene glycol in the main text.

Introduction:
"solely dependent on physical forces": membrane assembly is dependent on physical forces too. Rephrase

Done.

The first sentence of the LLPS introduction paragraph should mention that they are not membrane bound.

Done.

"are typically organized into complex, multilayered structures" - it is probably not true that condensates comprising multiple components are necessarily multilayered.

Done. We have also included additional references describing the multilayered organisation of several biomolecular condensates.

MAJOR - the introduction does not discuss the questions in the field at all. The introduction should be reworked to say something about Nsp5 and Nsp2 proposed role and then say what they may do. It should probably also introduce them as E/D/S rich and R rich, respectively, and disordered. This section of the introduction also seems like a list of results which is should not be as said by an earlier review.

We thank the reviewer for this comment, and we have re-written the Introduction section. For the revised version, we have attempted to produce a brief introduction that

would appeal to and remain accessible to both virologists/rotavirus experts, as well as those with interests in biomolecular condensates.

Results

MAJOR - it is not clear to my untrained eye from the data presented that the EM image contains for sure the viroplasm. Shouldn't immunogold labeling be done to show this structure is enriched in Nsp5/2 or something else to show what it is. Further, what else are we looking at and are there membranes here? Is the cell membrane forming vesicles and that is what is peeling off into the shapes we see as double layered RNA containing particles? For a broad audience, more information is needed.

There are many examples of published EM data that are over three decades old, including immunogold staining of NSP5 and NSP2 components of viroplasms. We have added some of these references in the new revised Introduction. We simply added this EM image to illustrate that the structures we are imaging indeed represent bona fide viral replication factories (aka viroplasms) in NSP5-EGFP cells, as these images clearly show emerging double-layered virus particles (which would not be visible through diffraction-limited microscopy). We refer the reader to a number of research papers that describe EM studies of viroplasms, which are in fact membraneless organelles, and these studies clearly show that they are rotavirus replication factories. New references from older studies (Altenburg *et al*, 1980; Petrie *et al*, 1984; Eichwald *et al*, 2018) **have now been added to refer the readership to the original immunogold identification of NSP5 and NSP2 in viroplasms.**

"As most typical properties of liquid" this sentence is not clear.

Thank you for pointing this out. We rephrased this to 'As liquid-like properties of droplets'

Figure 2c just shows an evaluation of sphericity but should show an image, or at least the main text should have a reference to the figure 1 or 2 panels where round droplets are seen in cells. Time axis should be in hours.

We have referred the reader to the panel a in the figure, where differently shaped droplets can be seen. Time axes in panels c and d are now shown in hours.

MAJOR: it is not clear why the authors used 1,2 and 1,3 propane diol instead of other diols. For example, 2,5 hexanediol is thought to be less effective. Nevertheless, their observation of propylene glycol is very interesting. I do suggest staying consistent in the figure labeling it as "PG" instead of "1,2PD" because next to 1,6HD you imagine that the authors are showing pentanediol instead of propane diol.

We have changed the labelling in the figure to avoid any confusion. The rationale behind using 1,3 and 1,2 propane diols (propylene glycol) is given in the main text. Previous studies did not explain the choice of 2,5 hexanediol either, so we cannot comment on that.

"anisotropic" is used throughout but this means "(of an object or substance) having a physical property that has a different value when measured in different directions. A simple example

is wood, which is stronger along the grain than across it." So the authors need a different word - irregularly shaped?

We have changed the wording to irregularly shaped.

MAJOR: The authors show CD data to say that the protein is 40% disordered. The authors should show the residuals/fit-line for the CD data to see if these values are really well explained by the model. Furthermore, they should show disorder prediction (DISOPRED) and secondary structure prediction (PSI-PRED) (both are available from PSI-PRED webserver) vs residue for this protein. Finally, they should show domain structure or predicted domain structure. I suggest updating their discussion of the domains and also the deltaCTD variant using something like the presentation in Perdikari et al EMBO J 2020 https://www.embopress.org/doi/full/10.15252/embj.2020106478 where they show the impact of domains on phase separation of SARS-CoV-2 nucleocapsid protein. The authors may also consider looking at alphafold to see if the structure of the structured regions can be predicted.

We thank the reviewer for these comments. We have used an improved, the most recently developed predictor of protein disorder flDPnn, whose predictions have been shown to outperform the results of the existing disorder predictors and methods that predict functions of disorder based on the recent Critical Assessment of protein Intrinsic Disorder prediction (CAID) experiment (Hu *et al*, 2021). These predictions have now been included into the new Figure 5. In Perdikari et al, the authors show a schematic of the domain organisation of a structured protein with IDPs. However, NSP5 is much smaller, and mainly disordered, with no high-resolution structural data available for any of its domains to date. Therefore, we avoided defining domains without any structural data. However, we have carried out the Aphafold predictions, and the results are also included in the new Figure 5.

MAJOR: the authors don't say anything about how the radius of hydration measurement tells them anything about the structure of the protein. Also, isn't Nsp5 dimeric? How is this considered here?

We have included the DLS data to merely demonstrate that refolded NSP5 forms higher order species, matching those described by (Martin *et al*, 2011)**. In this seminal study, the authors carried out the most detailed solution characterisation of the NSP5, in which they have convincingly demonstrated that NSP5 forms decameric species at micromolar concentrations. Higher protein concentrations led to the assembly of even larger species with poorly defined stoichiometry. Thus, in short, NSP5 is not dimeric in solution, and the hydrodynamic radius estimated from our DLS data closely matches the one for a decameric NSP5 species previously published by** (Martin *et al*, 2011)**. More importantly, The same study by Martin et al, 2011 has also demonstrated that removal of 18 C-terminal residues of NSP5 resulted in the loss of NSP5 oligomerisation in solution. Based on these results, and the results of our LLPS assays with the C-terminal NSP5 mutant, we propose that NSP5 oligomerisation is important for driving LLPS *in vitro*. Excitingly, these results are also consistent with our recently published study** (Papa *et al*, 2020)**, in which we have demonstrated that these C-terminal residues were i) critical for viral replication; ii) formation of viroplasms, thus further confirming the functional importance of this oligomerisation region in the formation of viral replication factories through LLPS of NSP5.**

The EV2a chromatogram should be labeled with the protein we are looking at as well as 280 and 260. The 260/280 ratio should be explicitly listed

Done.

"lacks a pi cloud" - I think a better wording would be "lacks sp2 hybridized groups in its sidechain". Also the difference between lys and arginine sidechain is not going to be about cation-pi as both are cations. Better to say that pi interactions are enhanced by arginine.

We have rephrased this sentence.

MAJOR: the authors mention that they use Arg-9 at the same concenetraiton as polyArg but are they counting in mass/volume equivalents (mg/ml) or in micromolar? They should do the experiment at equivalent mg/ml only.

Agreed, 150 μM Arg-9 was used instead of 5 μM polyArg, which corresponds to the same mass/volume (0.2 mg/ml) concentration. However, all concentrations are reported in μM units for consistency.

MAJOR: it is exciting to use the highthroughput method to generate the phase diagram, but the authors need to test the values at least at a single concentration of one component (or maybe in a "t" or "cross") to show what these values mean, especially as things at the edges of their data are "ragged" and look unclear. At the very least, the authors should make reference to how the results from this assay have been compared to bulk macro studies in another system.

We thank the reviewer for appreciating this approach. We would like to point out that the concentration regime we used is in low micromolar range, and using microfluidics-based approach allows us to detect condensate formation with high sensitivity. We have added more micrographs to demonstrate condensation of NSP5/NSP2 in the same concentration range, as used for PhaseScan. These images are now presented in the updated Figure EV4.

In figure 6 the authors should show the different channels separately and then show the merge. Also, some measure of colocalization in the Nsp area would be useful.

We presented RNA FISH intensity signals quantification data in Figure EV5. We avoided using any colocalization analyses as the main goal of showing these images was to demonstrate reversible dissolution of RNA-containing viroplasms. Of course, in case of complete disappearance of NSP5-EGFP-tagged viroplasms upon PG application, colocalization equals zero. Upon PG removal, multiple RNA granules emerge, and the number of EGFP granules is smaller than the number of multiple individual RNA spots, therefore colocalization techniques yield low correlation coefficients. We thus feel that adding colocalization analyses would not assist in data interpretation in this case.

"the observed effect" what effect?

Fixed this typo, thank you for pointing that out.

The labeling in Fig 6e is hard to follow.

Thank you for the suggestion to clarify the labelling, we have changed labelling to improve the clarity.

MAJOR: The authors need to show that the higher bands are phosphorylated (for example by phosphatase treatment gel) or point to the specific literature showing that this pattern has been observed before for phosphor Nsp5

Multiple studies (Fabbretti *et al*, 1999; Poncet *et al*, 1997; Sen *et al*, 2006; Sotelo *et al*, 2010; Campagna *et al*, 2007; Criglar *et al*, 2018; Eichwald *et al*, 2018; Papa *et al*, 2020) have extensively characterised NSP5 phosphorylation, including the most recent study from our group (Papa *et al*, 2020), where we directly demonstrated that the apparent higher molecular weight bands that appear on a gel are due to the hyperphosphorylation of NSP5. Older studies employed phosphatases, kinase inhibitors, siRNAs targeting casein kinases etc to demonstrate that these extra bands represent hyperphosphorylated forms of NSP5. In this work, we have used a previously validated anti-NSP5 antibody sample that had been used for the (Papa *et al*, 2020) **study, in which we demonstrated that they recognise both non-phosphorylated and hyperphosphorylated forms of NSP5.**

"less isotropic" see above - replace with "less round"

Done.

Discussion
The paragraph starting "coacervation of viral RNA chaperone" seems to go on about RNA-RNA interactions which is not probed at all in the paper and seems disconnected.

We thank the reviewer for their comment, although we would like to keep this paragraph to highlight the potential implications of the LLPS during RNA assortment in multi-segmented genomes, e.g., in rotaviruses. In our view, LLPS of viral RNA chaperones and viral transcript enrichment into these condensates presents an exciting avenue for future mechanistic studies of segmented genome packaging in dsRNA viruses.

A mention in the discussion linking to nucleocapsid protein phase separation that has been examine for SARS-CoV-2 as well as the work on measles virus nucleo and phosphoprotein LLPS by Sigrid Milles et al should be included. Are these related? Does Nsp5/2 recruit human proteins in the condensates?

All these references are mentioned throughout the Introduction and Discussion sections. We would also like to thank the reviewer for this comment, as we have also realized that most viral condensates described to date have indeed reported the involvement of structural viral proteins, i.e., those present in mature virus particles. In RVs, both drivers of LLPS are not present in mature virions. Whether NSP5/NSP2 recruit human proteins remains an open question that extends beyond the scope of this study. However, we would like to note that we expect that the protein composition of

viroplasms may significantly vary during the early/late infection stages. Careful analysis of early vs late infection stage viroplasms must be carried out *in situ*, to be able to link their liquid-like properties during the early infection stages with NSP5 phosphorylation state, RNA composition, and their further maturation during late infection stages.

Methods
Why is sodium fluoride used for the CD? The authors should mention in the methods.

We added a sentence to the Materials and Methods. Using NaF instead of NaCl is a common practice in spectroscopic measurements, since Cl^- ions have a strong UV absorbance at low wavelengths (Whitmore & Wallace, 2008), and NSP5 requires higher ionic strength to prevent its aggregation in solution (Martin *et al*, 2011). We thus chose to use fluoride rather than chloride ions.

References
Some references I noticed were messed up
Conicella et al. and Krosschwald et al ("Simon A"?) have errors. Other references likely do too. I suggest the authors check their reference library or reference strategy.

Thank you for pointing this out. These references have now been fixed.

Some additional comments from a student reviewer are below. Note, I find that the main idea that Nsp5 is the scaffold well supported, but I suggest looking at these detailed comments and possibly performing experiments suggested to clarify the matter.

Is addition of PEG a diagnostic of a "scaffold protein"? In the first submission, isolated NSP5 (the so-called "scaffold") does not undergo LLPS without NSP2. In the revised manuscript, they show that 35 uM NSP5 + PEG-20K forms tiny droplets (Fig. 4A bottom panel). In contrast, 25 uM of NSP2 + PEG-20 K does not. Why didn't they test NSP2 and NSP5 at the same concentration (35 uM) in the presence of PEG-20K?

We thank the reviewer, and their student for their comments. We didn't test NSP2 at higher concentration because it undergoes severe aggregation at concentrations above 25 μM in the presence of PEG-20K. In contrast, NSP5 remains soluble at much higher concentration in the presence of PEG-20K.

3. In Figure 5c top, they deleted the CTR oligomerization domain and they show that 25 uM delta-CTR in the presence of PEG and poly-arginine doesn't undergo LLPS. 10 uM NSP5 mixed with 10 uM NSP2 triggers phase separation but in this case, 25 uM NSP5 delta-CTR + 10 uM NSP2 does not. In Figure 5c bottom, they mixed 10 uM NSP2 + 25 uM NSP5 + 5 uM NSP5 delta-CTR to show that the deletion construct partitions into preformed NSP5/NSP2 droplets. They interpret this result by stating that delta-CTR is capable of forming heterotypic (with NSP2) and homotypic interactions (with NSP5). Does this experiment provide enough evidence for a protein to be called a "scaffold"? Does stoichiometric excess of NSP5 favor homotypic interactions?

We do not interpret this result as evidence of NSP5 being a scaffold. We intended to demonstrate that NSP5 oligomerisation is likely to be linked to its capability to undergo condensation. We also did not interpret the result as delta-CTR being capable of forming heterotypic interactions with NSP2. Note that irrespective of client, NSP5

forms droplets, either with NSP2, or with poly-arginine. Unlike for NSP5:NSP2 system, higher NSP5:poly-arginine ratios were required for efficient droplet formation, as we demonstrate in the manuscript.

Minor comments:
• Figure EV3: NSP2/NSP5 phase separates in physiological NaCl concentration but they don't mention the exact NaCl mM concentration anywhere in the text.

Physicological phosphate saline buffer, PBS, pH 7.4, corresponding to ~ 150 mM NaCl).

• Figure 1: It would be helpful to have a domain description of NSP2 and NSP5. Also, in the introduction they don't include details about the sequence composition and the biophysical characteristics of these sequences (length, charge, % disorder etc).

Please see our comments above.

• They have an interesting method for making phase diagrams using a microfluidic device. In Figure 4b, they show how hexanediol creates one phase versus two phases. Since propylene glycol is safer than hexanediol and efficient in dissolving condensates, it would be interesting to make a phase diagram in the presence of propylene glycol as well. Also the coloring in Figure EV4e (LLPS: blue, mixed:red) is not consistent with Figure 4b (LLPS: red, mixed: blue). I am not sure how Figure 4EV e presents more information compared to Figure 4b.

We have removed Figure 4EV panel e, the color schemes are not consistent throughout the manuscript.

References:

Altenburg BC, Graham DY & Estes MK (1980) Ultrastructural Study of Rotavirus Replication in Cultured Cells. *J Gen Virol* 46: 75–85

Campagna M, Budini M, Arnoldi F, Desselberger U, Allende JE & Burrone OR (2007) Impaired hyperphosphorylation of rotavirus NSP5 in cells depleted of casein kinase 1α is associated with the formation of viroplasms with altered morphology and a moderate decrease in virus replication. *J Gen Virol* 88: 2800–2810

Criglar JM, Anish R, Hu L, Crawford SE, Sankaran B, Prasad BVV & Estes MK (2018) Phosphorylation cascade regulates the formation and maturation of rotaviral replication factories. *Proc Natl Acad Sci*: 201717944

Eichwald C, De Lorenzo G, Schraner EM, Papa G, Bollati M, Swuec P, de Rosa M, Milani M, Mastrangelo E, Ackermann M, *et al* (2018) Identification of a Small Molecule That Compromises the Structural Integrity of Viroplasms and Rotavirus Double-Layered Particles. *J Virol* 92

Fabbretti E, Afrikanova I, Vascotto F & Burrone OR (1999) Two non-structural rotavirus proteins, NSP2 and NSP5, form viroplasm-like structures in vivo. *J Gen Virol* 80: 333–339

Hu G, Katuwawala A, Wang K, Wu Z, Ghadermarzi S, Gao J & Kurgan L (2021) flDPnn: Accurate intrinsic disorder prediction with putative propensities of disorder functions. *Nat Commun* 12: 4438

Martin D, Ouldali M, Ménétrey J & Poncet D (2011) Structural organisation of the rotavirus nonstructural protein NSP5. *J Mol Biol* 413: 209–221

Papa G, Venditti L, Arnoldi F, Schraner EM, Potgieter C, Borodavka A, Eichwald C & Burrone OR (2020) Recombinant rotaviruses rescued by reverse genetics reveal the role of NSP5 hyperphosphorylation in the assembly of viral factories. *J Virol* 94: 1–23

Petrie BL, Greenberg HB, Graham DY & Estes MK (1984) Ultrastructural localization of rotavirus antigens using colloidal gold. *Virus Res* 1: 133–152

Poncet D, Lindenbaum P, L'Haridon R & Cohen J (1997) In vivo and in vitro phosphorylation of rotavirus NSP5 correlates with its localization in viroplasms. *J Virol* 71: 34–41

Sen A, Agresti D & Mackow ER (2006) Hyperphosphorylation of the rotavirus NSP5 protein is independent of serine 67 or NSP2 and the intrinsic insolubility of NSP5 is regulated by cellular phosphatases. *J Virol* 80: 1807–1816

Sotelo PH, Schümann M, Krause E & Chnaiderman J (2010) Analysis of rotavirus non-structural protein NSP5 by mass spectrometry reveals a complex phosphorylation pattern. *Virus Res* 149: 104–108

Whitmore L & Wallace B a (2008) Protein secondary structure analyses from circular dichroism spectroscopy: methods and reference databases. *Biopolymers* 89: 392–400

2nd Revision - Editorial Decision 27th Aug 2021

Thank you for submitting your final revised manuscript for our consideration. I am pleased to inform you that we have now accepted it for publication in The EMBO Journal.

EMBO PRESS

YOU MUST COMPLETE ALL CELLS WITH A PINK BACKGROUND

PLEASE NOTE THAT THIS CHECKLIST WILL BE PUBLISHED ALONGSIDE YOUR PAPER

Alexander Borodavka
EMBO J
EMBOJ-2021-107711R-Q

Reporting Checklist For Life Sciences Articles (Rev. June 2017)

This checklist is used to ensure good reporting standards and to improve the reproducibility of published results. These guidelines are consistent with the Principles and Guidelines for Reporting Preclinical Research issued by the NIH in 2014. Please follow the journal's authorship guidelines in preparing your manuscript.

A- Figures
1. Data
The data shown in figures should satisfy the following conditions:

- the data were obtained and processed according to the field's best practice and are presented to reflect the results of the experiments in an accurate and unbiased manner.
- figure panels include only data points, measurements or observations that can be compared to each other in a scientifically meaningful way.
- graphs include clearly labeled error bars for independent experiments and sample sizes. Unless justified, error bars should not be shown for technical replicates.
- if n< 5, the individual data points from each experiment should be plotted and any statistical test employed should be justified
- Source Data should be included to report the data underlying graphs. Please follow the guidelines set out in the author ship guidelines on Data Presentation.

2. Captions

Each figure caption should contain the following information, for each panel where they are relevant:

- a specification of the experimental system investigated (eg cell line, species name).
- the assay(s) and method(s) used to carry out the reported observations and measurements
- an explicit mention of the biological and chemical entity(ies) that are being measured.
- an explicit mention of the biological and chemical entity(ies) that are altered/varied/perturbed in a controlled manner.

- the exact sample size (n) for each experimental group/condition, given as a number, not a range;
- a description of the sample collection allowing the reader to understand whether the samples represent technical or biological replicates (including how many animals, litters, cultures, etc.).
- a statement of how many times the experiment shown was independently replicated in the laboratory.
- definitions of statistical methods and measures:
  - common tests, such as t-test (please specify whether paired vs. unpaired), simple χ2 tests, Wilcoxon and Mann-Whitney tests, can be unambiguously identified by name only, but more complex techniques should be described in the methods section;
  - are tests one-sided or two-sided?
  - are there adjustments for multiple comparisons?
  - exact statistical test results, e.g., P values = x but not P values < x;
  - definition of 'center values' as median or average;
  - definition of error bars as s.d. or s.e.m.

Any descriptions too long for the figure legend should be included in the methods section and/or with the source data.

In the pink boxes below, please ensure that the answers to the following questions are reported in the manuscript itself. Every question should be answered. If the question is not relevant to your research, please write NA (non applicable). We encourage you to include a specific subsection in the methods section for statistics, reagents, animal models and human subjects.

B- Statistics and general methods

Please fill out these boxes (Do not worry if you cannot see all your text once you press return)

1.a. How was the sample size chosen to ensure adequate power to detect a pre-specified effect size?	Where appropriate (effects of PG treatments on RV titres), experiments were carried out independently in 3 replicates to compare PG-treated group vs the control. No additional tests to determine the sample size were required.
1.b. For animal studies, include a statement about sample size estimate even if no statistical methods were used.	N/A
2. Describe inclusion/exclusion criteria if samples or animals were excluded from the analysis. Were the criteria pre-established?	N/A
3. Were any steps taken to minimize the effects of subjective bias when allocating animals/samples to treatment (e.g. randomization procedure)? If yes, please describe.	N/A
For animal studies, include a statement about randomization even if no randomization was used.	N/A
4.a. Were any steps taken to minimize the effects of subjective bias during group allocation or/and when assessing results (e.g. blinding of the investigator)? If yes please describe.	N/A
4.b. For animal studies, include a statement about blinding even if no blinding was done	N/A
5. For every figure, are statistical tests justified as appropriate?	Where appropriate, the mean and standard deviations (SD) were calculated from repeated measurements (For biological replicates, typically n= 3-5, as specified in Figure legends). Where appropriate, statistical analyses of data were performed as follows: Fig. 6: Datasets from four independent repeats were compared by a two-tailed Mann-Whittney test. For three groups comparisons, a Kruskal-Wallis test followed by uncorrected Dunn's multiple comparison test was used. For Fig.7, a Kolmogorov-Smirnov test was applied to detect differences in the observed distributions, rather their mean values, as this test is more appropriate for detecting changes in sizes of RNA clusters that constitute a wide population of species that grow in sizes between 4-6 hpi. The two-sample K–S test was used as it is sensitive to differences in both location and shape of the cumulative distribution functions of the two samples. All Mean and SD values, with N numbers are reported in Figure legends.

USEFUL LINKS FOR COMPLETING THIS FORM

http://www.antibodypedia.com
http://1degreebio.org
http://www.equator-network.org/reporting-guidelines/improving-bioscience-research-repor

http://grants.nih.gov/grants/olaw/olaw.htm
http://www.mrc.ac.uk/Ourresearch/Ethicsresearchguidance/Useofanimals/index.htm
http://ClinicalTrials.gov
http://www.consort-statement.org
http://www.consort-statement.org/checklists/view/32-consort/66-title
http://www.equator-network.org/reporting-guidelines/reporting-recommendations-for-tumo
http://datadryad.org
http://figshare.com
http://www.ncbi.nlm.nih.gov/gap
http://www.ebi.ac.uk/ega
http://biomodels.net/
http://biomodels.net/miriam/
http://jjj.biochem.sun.ac.za
http://oba.od.nih.gov/biosecurity/biosecurity_documents.html
http://www.selectagents.gov/

Do the data meet the assumptions of the tests (e.g., normal distribution)? Describe any methods used to assess it.	See comments above.
Is there an estimate of variation within each group of data?	Yes
Is the variance similar between the groups that are being statistically compared?	Yes

C- Reagents

6. To show that antibodies were profiled for use in the system under study (assay and species), provide a citation, catalog number and/or clone number, supplementary information or reference to an antibody validation profile. e.g., Antibodypedia (see link list at top right), 1DegreeBio (see link list at top right).	1. Polyclonal NSP5-specific antibodies described in Papa, G. et al. Recombinant rotaviruses rescued by reverse genetics reveal the role of NSP5 hyperphosphorylation in the assembly of viral factories. J. Virol. 94, 1–23 (2019). 2. Anti-guinea pig IgG (H+L) cross-adsorbed secondary antibody - Invitrogen, #SA5-10100. 3. anti-actin hFAB rhodamine antibody (Biorad, cat. #12004164).
7. Identify the source of cell lines and report if they were recently authenticated (e.g., by STR profiling) and tested for mycoplasma contamination.	MA104 cells (ATCC CRL-2378.1), and their derivatives were routinely tested for Mycoplasma sp. Contamination (Mycospy Biontex).

* for all hyperlinks, please see the table at the top right of the document

D- Animal Models

8. Report species, strain, gender, age of animals and genetic modification status where applicable. Please detail housing and husbandry conditions and the source of animals.	N/A
9. For experiments involving live vertebrates, include a statement of compliance with ethical regulations and identify the committee(s) approving the experiments.	N/A
10. We recommend consulting the ARRIVE guidelines (see link list at top right) (PLoS Biol. 8(6), e1000412, 2010) to ensure that other relevant aspects of animal studies are adequately reported. See author guidelines, under 'Reporting Guidelines'. See also: NIH (see link list at top right) and MRC (see link list at top right) recommendations. Please confirm compliance.	N/A

E- Human Subjects

11. Identify the committee(s) approving the study protocol.	N/A
12. Include a statement confirming that informed consent was obtained from all subjects and that the experiments conformed to the principles set out in the WMA Declaration of Helsinki and the Department of Health and Human Services Belmont Report.	N/A
13. For publication of patient photos, include a statement confirming that consent to publish was obtained.	N/A
14. Report any restrictions on the availability (and/or on the use) of human data or samples.	N/A
15. Report the clinical trial registration number (at ClinicalTrials.gov or equivalent), where applicable.	N/A
16. For phase II and III randomized controlled trials, please refer to the CONSORT flow diagram (see link list at top right) and submit the CONSORT checklist (see link list at top right) with your submission. See author guidelines, under 'Reporting Guidelines'. Please confirm you have submitted this list.	N/A
17. For tumor marker prognostic studies, we recommend that you follow the REMARK reporting guidelines (see link list at top right). See author guidelines, under 'Reporting Guidelines'. Please confirm you have followed these guidelines.	N/A

F- Data Accessibility

18: Provide a "Data Availability" section at the end of the Materials & Methods, listing the accession codes for data generated in this study and deposited in a public database (e.g. RNA-Seq data: Gene Expression Omnibus GSE39462, Proteomics data: PRIDE PXD000208 etc.) Please refer to our author guidelines for 'Data Deposition'.<br><br>Data deposition in a public repository is mandatory for:<br>a. Protein, DNA and RNA sequences<br>b. Macromolecular structures<br>c. Crystallographic data for small molecules<br>d. Functional genomics data<br>e. Proteomics and molecular interactions	Primary image datasets are available at http://doi.org/10.5281/zenodo.5009222.
19. Deposition is strongly recommended for any datasets that are central and integral to the study; please consider the journal's data policy. If no structured public repository exists for a given data type, we encourage the provision of datasets in the manuscript as a Supplementary Document (see author guidelines under 'Expanded View' or in unstructured repositories such as Dryad (see link list at top right) or Figshare (see link list at top right).	Primary image datasets are available at http://doi.org/10.5281/zenodo.5009222.
20. Access to human clinical and genomic datasets should be provided with as few restrictions as possible while respecting ethical obligations to the patients and relevant medical and legal issues. If practically possible and compatible with the individual consent agreement used in the study, such data should be deposited in one of the major public access-controlled repositories such as dbGAP (see link list at top right) or EGA (see link list at top right).	N/A
21. Computational models that are central and integral to a study should be shared without restrictions and provided in a machine-readable form. The relevant accession numbers or links should be provided. When possible, standardized format (SBML, CellML) should be used instead of scripts (e.g. MATLAB). Authors are strongly encouraged to follow the MIRIAM guidelines (see link list at top right) and deposit their model in a public database such as Biomodels (see link list at top right) or JWS Online (see link list at top right). If computer source code is provided with the paper, it should be deposited in a public repository or included in supplementary information.	N/A

G- Dual use research of concern

22. Could your study fall under dual use research restrictions? Please check biosecurity documents (see link list at top right) and list of select agents and toxins (APHIS/CDC) (see link list at top right). According to our biosecurity guidelines, provide a statement only if it could.	N/A
